# Revision of *Potamobates* Champion, 1898 (Hemiptera: Heteroptera: Gerridae) and description of a new genus for *P*. *thomasi* Hungerford, 1937

**DOI:** 10.1371/journal.pone.0280405

**Published:** 2023-03-08

**Authors:** Carla Fernanda Burguez Floriano, Felipe Ferraz Figueiredo Moreira, Pitágoras da Conceição Bispo

**Affiliations:** 1 Laboratório de Biologia Aquática, Departamento de Ciências Biológicas, Universidade Estadual Paulista, Assis, SP, Brazil; 2 Laboratório de Biodiversidade Entomológica, Instituto Oswaldo Cruz, Fundação Oswaldo Cruz, Rio de Janeiro, RJ, Brazil; Universita degli Studi di Roma La Sapienza, ITALY

## Abstract

*Potamobates* Champion, 1898 (Hemiptera: Heteroptera: Gerridae) heretofore included 18 species distributed from southern Mexico to Peru. They display a distinct morphology, especially regarding the projections of abdominal segment VIII. Specific identification and delimitation are difficult, and the genus lacks a thorough revision and evaluation of inter- and intraspecific variation. Here, we revise *Potamobates*, redescribe and/or illustrate known species, and describe *P*. *molanoi* Floriano and Moreira, **sp. nov.** and *Brailovskybates* Floriano and Moreira, **gen. nov.** The new genus is erected for *P*. *thomasi* Hungerford, 1937 and is characterized by the following features: (1) abdomen elongated, longer than the mesothorax; (2) abdominal spiracles positioned at the center of the segments; (3) male abdominal segment VIII without projections; (4) male pygophore and proctiger not rotated in relation to the longitudinal axis of the body; (5) female abdominal tergum VIII subequal in length and width; (6) and posterior margin of female abdominal sternum VII not produced medially, with a pair of lateral projections.

## Introduction

The subfamily Cylindrostethinae (Hemiptera: Heteroptera: Gerridae) comprises the genera *Cylindrostethus* Mayr, 1865; *Platygerris* White, 1883; and *Potamobates* Champion, 1898. *Cylindrostethus* is Pantropical, with 18 species (nine Neotropical and nine Paleotropical) [1, 2]. *Platygerris* occurs in Central America and northern South America, and includes five species [2, 3]. *Potamobates* is distributed from southern Mexico to Peru and holds 18 species [4–7].

Representatives of *Potamobates* are gregarious and occupy the surface of slow flowing rivers and streams [7]. The genus is characterized by the abdominal spiracles located closer to the posterior margins than to the anterior margins of the segments; and the male with the posterior projection of the last abdominal laterotergite (= connexival spine) reduced or absent, the posterior margin of abdominal segment VIII and the proctiger asymmetrical, and the pygophore and proctiger sinistrally rotated [2, 6].

*Potamobates* was described by Champion (1898) [8] to include his two new species *Po*. *bidentatus* Champion, 1898 and *Po*. *unidentatus* Champion, 1898. Subsequently, several other species were described in isolated papers, such as *Po*. *horvathi* Esaki, 1926; *Po*. *osborni* Drake and Harris, 1928; *Po*. *peruvianus* Hungerford, 1936; *Po*. *spiculus* Polhemus and Polhemus, 1983; *Po*. *thomasi* Hungerford, 1937; *Po*. *tridentatus* Esaki, 1926; *Po*. *variabilis* Hungerford, 1938; *Po*. *vivatus* Drake and Roze, 1954; *Po*. *williamsi* Hungerford 1932; and *Po*. *woytkowskii* Hungerford, 1937 [9–16].

Both Kuitert (1942) [17] and Matsuda (1960) [2] redescribed the genus, and later Polhemus and Polhemus (1995) [6] described three new species (*Po*. *anchicaya* Polhemus and Polhemus, 1995; *Po*. *carvalhoi* Polhemus and Polhemus 1995; and *Po*. *manzanoae* Polhemus and Polhemus, 1995). The last authors also performed a phylogenetic analysis based on 15 terminal taxa and 10 ordered morphological characters. Based on the resulting topology, they proposed four groups of species, as follows: group *thomasi* (*Po*. *thomasi*); group *unidentatus* (*Po*. *bidentatus*, *Po*. *horvathi*, *Po*. *manzonoae*, *Po*. *osborni*, and *Po*. *unidentatus*); group *carvalhoi* (*Po*. *carvalhoi*); and group *tridentatus* (*Po*. *peruvianus*, *Po*. *spiculus*, *Po*. *tridentatus*, *Po*. *variabilis*, *Po*. *vivatus*, *Po*. *williamsi*, and *Po*. *woytkowskii*).

In turn, Cognato (1998) [18] described *Po*. *sumaco* Cognato, 1998 and proposed an additional step to the identification key originally published by Polhemus and Polhemus (1995) [6]. Furthermore, he added his new species and one new character to their matrix, and performed further analyses. More recently, Buzzetti (2006) [19] described *Po*. *shuar* Buzzetti, 2006, including his new species in Cognato’s matrix, and performed new analyses. Then, Padilla-Gil and Damgaard (2011) [7] did the same process when describing *Po*. *tumaquensis* Padilla-Gil and Damgaard, 2011 [7]. Subsequently, Morales et al. (2013) [5] described *Po*. *bilobulatus* Morales, Molano and Castro, 2013 from Colombia.

Despite the several phylogenetic analyses published so far based on modified versions of the matrix provided by Polhemus and Polhemus (1995), the relationships among *Potamobates* species are not yet clear due to the polytomies obtained and inconsistency of the proposed species groups with the resulting topologies. Additionally, in a recent analysis of Cylindrostethinae based on 114 morphological characters and 23 terminal taxa, the species groups defined in the literature have again not been recovered, and *Potamobates* resulted as paraphyletic and sister to *Platygerris*, while *Po*. *thomasi* was sister to both genera [20]. This species indeed displays several differences in relation to its current congeners and also does not fit into the other genera of the subfamily.

Finally, there is a high degree of intraspecific morphological variation that is only assessable when studying large series of specimens, which was not the case in many of the species descriptions mentioned above. These factors make delimiting some species of *Potamobates* confusing, even for researchers that are very experienced in dealing with gerromorphans. Therefore, we present here a revision of *Potamobates* and the contained species, including detailed descriptions based on many individuals whenever possible, illustrations, an identification key, and maps. We also describe a new genus for *Po*. *thomasi*, considering the disparity between this species and other Cylindrostethinae, and provide a key to the genera of the subfamily within this new context.

## Material and methods

The material examined is deposited in the following institutions: **AMNH**: American Museum of Natural History, New York, USA. **EQ:** eQual Consultoría y Servicios Ambientales, Bogotá, Colombia; **ICN:** Instituto de Ciencias Naturales, Universidad Nacional de Colombia, Bogotá, Colombia; **INVERTUN:** Colección de Invertebrados Acuáticos, Universidad Nacional de Colombia, Bogotá, Colombia; **LACM:** Natural History Museum of Los Angeles County, Los Angeles, USA; **MUSENUV:** Museo de Entomología, Universidad del Valle, Cali, Colombia; **MZUCR**: Colección de Entomología Acuática, Museo de Zoología, Universidad de Costa Rica, San Pedro de Montes de Oca, Costa Rica; **NHRS**: Naturhistoriska Riksmuseet, Stockholm, Sweden; **UCMC**: University of Colorado Museum of Natural History, Boulder, USA; **UPTC:** Museo de História Natural “Luiz Gonzalo Andrade”, Universidad Pedagógica y Tecnológica de Colombia, Tunja, Colombia; **USNM:** National Museum of Natural History, Smithsonian Institution, Washington D.C., USA.

Specimens were identified based on keys provided by Polhemus and Polhemus (1995) [6], Cognato (1998) [18], Buzzetti (2006) [19], and Padilla-Gil and Damgaard (2011) [7]. Identifications were confirmed by comparison with the original descriptions and type specimens. Species groups defined by Polhemus and Polhemus (1995) are not used in this study, because they are not consistent with the topologies obtained in subsequent analyses [7, 18–20]. Drawings were prepared using a camera lucida attached to a Leica M205A stereomicroscope. Then, the illustrations were scanned and vectorized using Adobe Illustrator CS5. Photographs were taken on the same stereomicroscope and edited using Adobe Photoshop CS5. Additionaly, at the USNM, types were photographed using a Canon EOS 5D camera at different focal lengths and the images combined using the software Visionary Digital. For the scanning electron micrographs, specimens were positioned in stubs, metallized, and analyzed using a Zeiss EVO/MA15. All measurements are presented in mm. Geographic distribution data were obtained from specimen labels and from the literature. When not originally indicated, approximate geographic coordinates of collecting localities were taken from Google Earth Pro. Maps were created using the software QGIS. Localities that are imprecise (e.g., only the country or state is known) are displayed on the maps with question marks.

### Nomenclatural acts

The electronic edition of this article conforms to the requirements of the amended International Code of Zoological Nomenclature, and hence the new names contained herein are available under that Code from the electronic edition of this article. This published work and the nomenclatural acts it contains have been registered in ZooBank, the online registration system for the ICZN. The ZooBank LSIDs (Life Science Identifiers) can be resolved and the associated information viewed through any standard web browser by appending the LSID to the prefix "http://zoobank.org/". The LSID for this publication is: urn:lsid:zoobank.org:pub: 7944B47D-5D81-442E-9362-483CC00951B0. The electronic edition of this work was published in a journal with an ISSN, and has been archived and is available from the following digital repositories: PubMed Central and LOCKSS.

## Results and discussion

### Key to the genera of Cylindrostethinae

**Fig 1:**
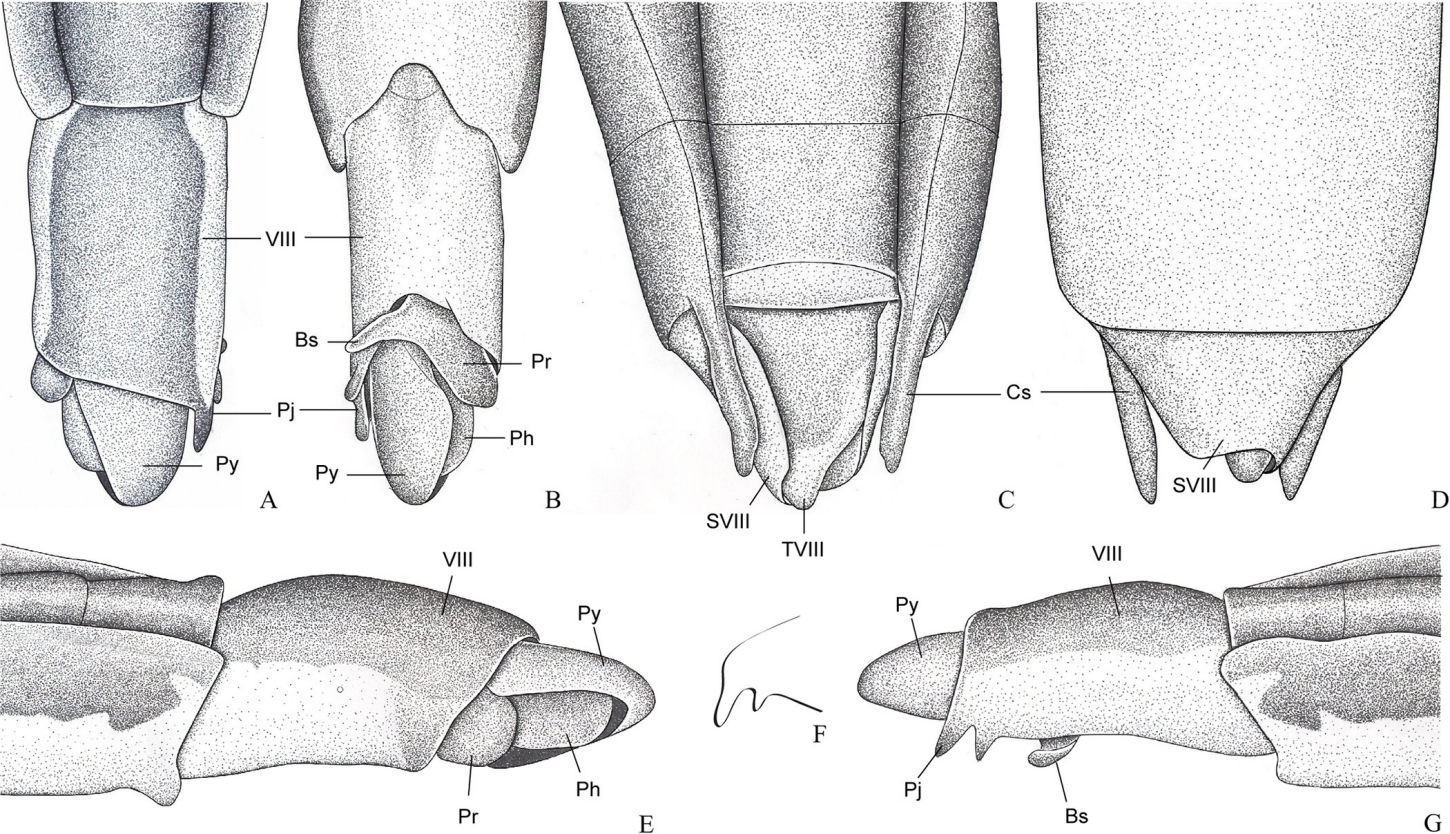
*Potamobates anchicaya*. (A) Male, terminalia, dorsal view; (B) male, terminalia, ventral view; (C) female, terminalia, dorsal view; (D) female, terminalia, vista ventral; (E) male, terminalia, left lateral view; (F) variation of projection VIII; (G) male, terminalia, right lateral view. **VIII**: segment VIII; **BS**: Processo basolateral of proctiger; **PR:** Proctiger; **PH**: Phallus; **PY:** Pigophore; **PJ:** Projection of segment VIII; **CS**: Connexival spine; **SVIII**: Sternun VIII.

**Fig 2:**
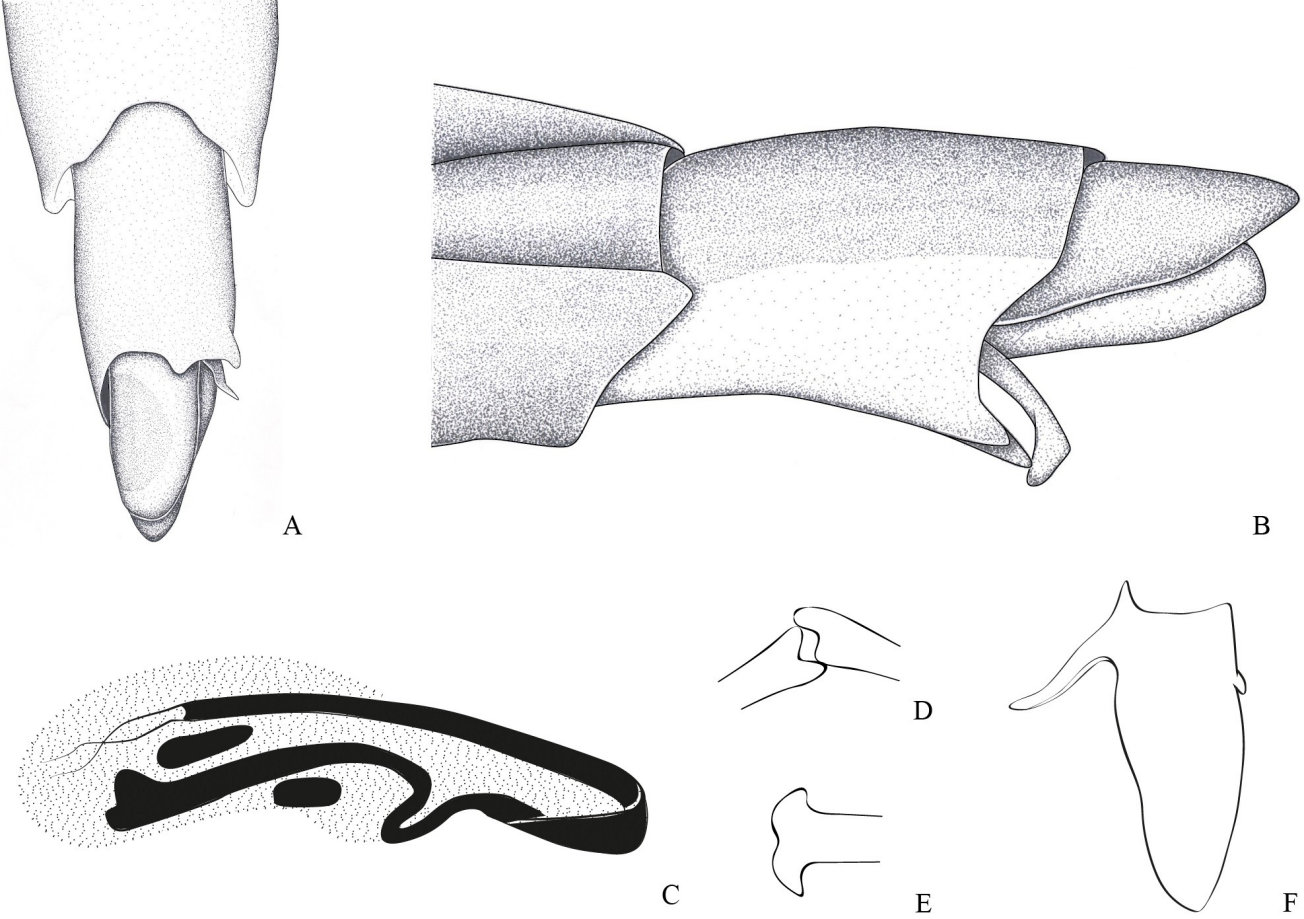
*Potamobates bidentatus*. (A) Male, terminalia, ventral view; (B) male, terminalia, vista left lateral view; (C) Phallus, lateral view; (D) junction between dorsal and ventral sclerite; (E) ventral sclerite base; (F) proctiger, dorsal view.

**Fig 3:**
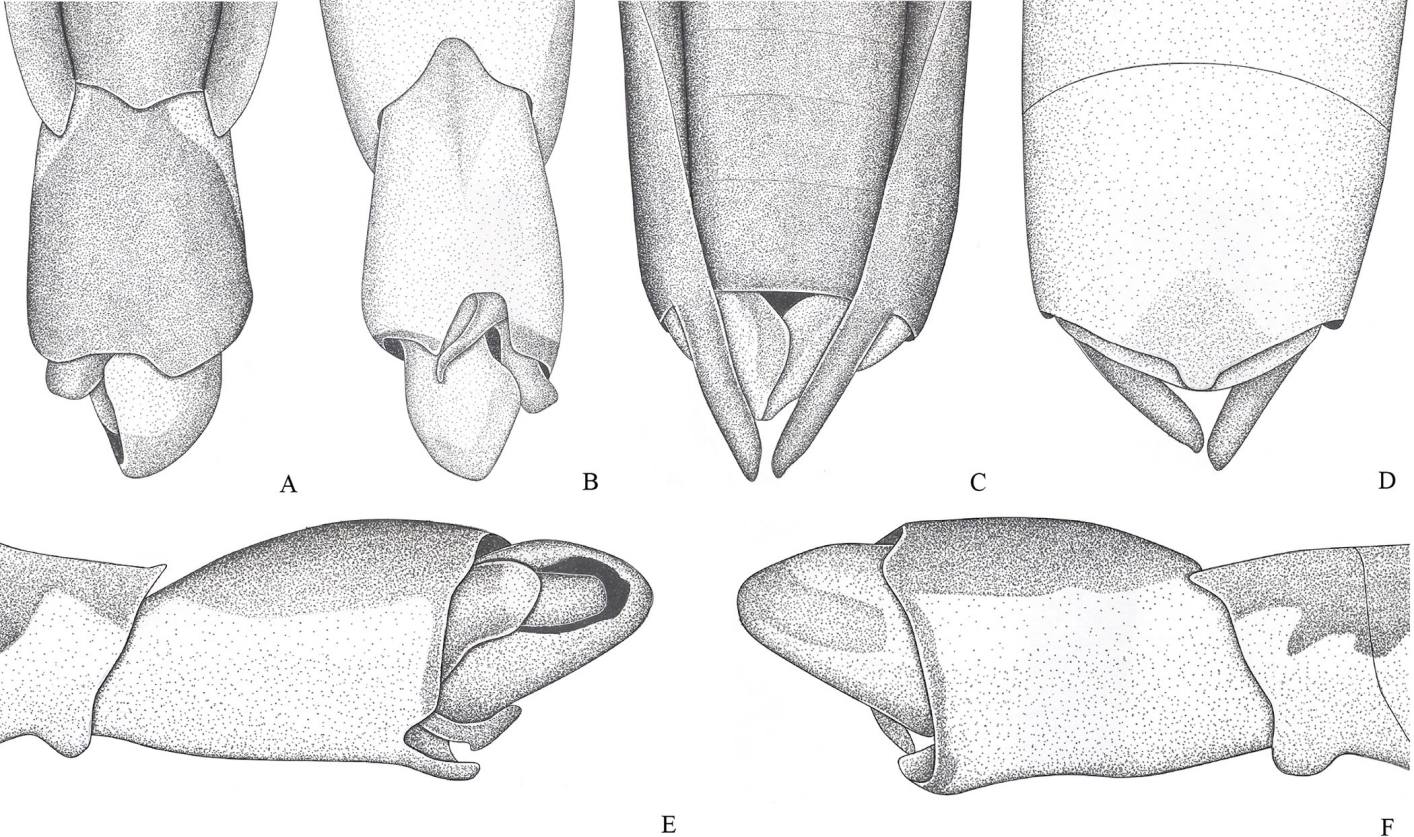
*Potamobates carvalhoi*. (A) Male, terminalia, dorsal view; (B) male, terminalia, ventral view; (C) female, terminalia, dorsal view; (D) female, terminalia, ventral view; (E) male, terminalia, left lateral view; (F) male, terminalia, right lateral view.

1– Male pygophore and proctiger sinistrally rotated (Figs 1B, 2B and 3A) ………………… 2

**Fig 4:**
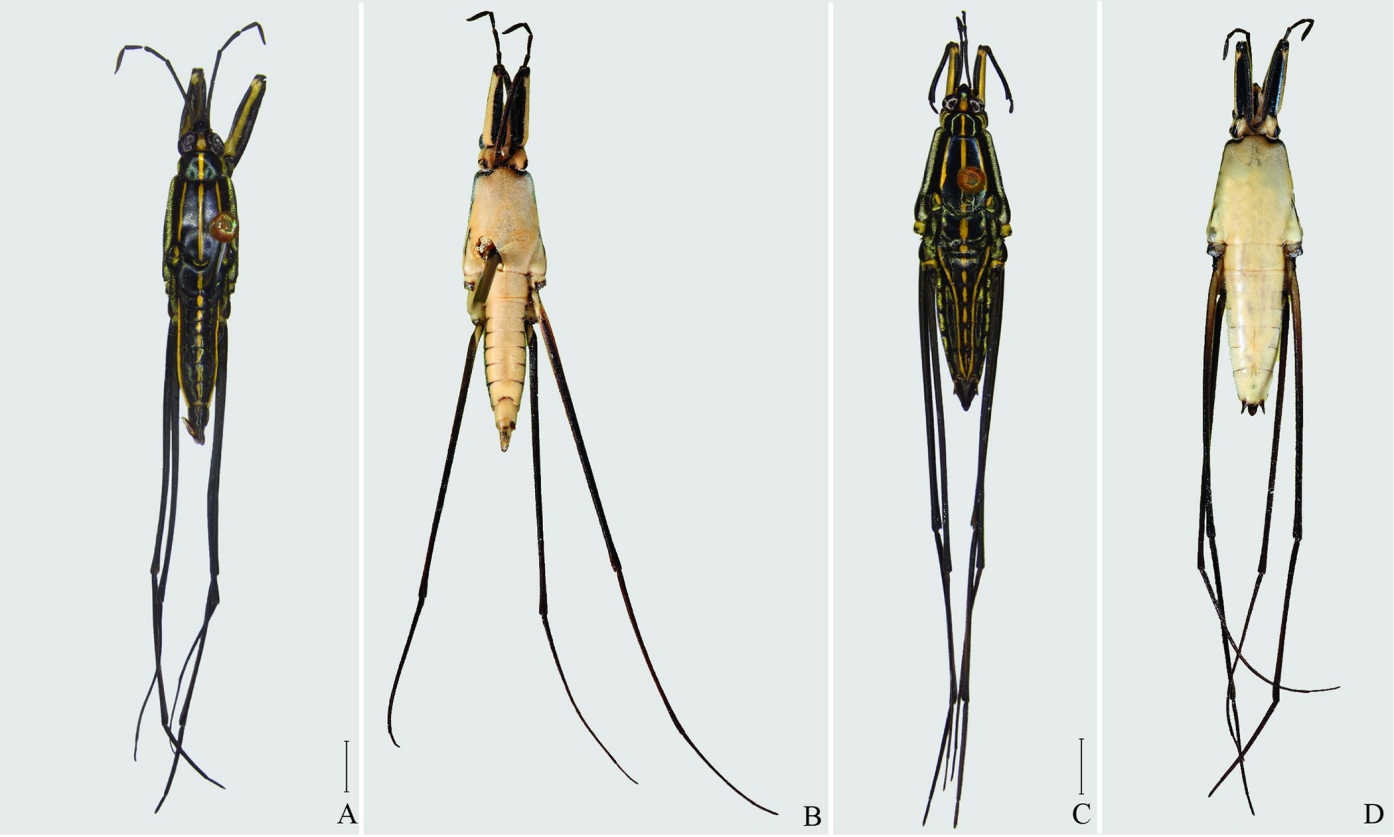
*Brailovskybates thomasi* new comb. (A) Male, dorsal view; (B) male, ventral view; (C) female, dorsal view; (D) female, ventral view.

1’–Male pygophore and proctiger not rotated (Fig 4A) ……………………………. 3

2– Mesonotum with a transversal C-shaped stripe formed by silvery setae; omphalium, lateral groove, and lateral evaporatorium of scent apparatus inconspicuous …… *Platygerris*

**Fig 5:**
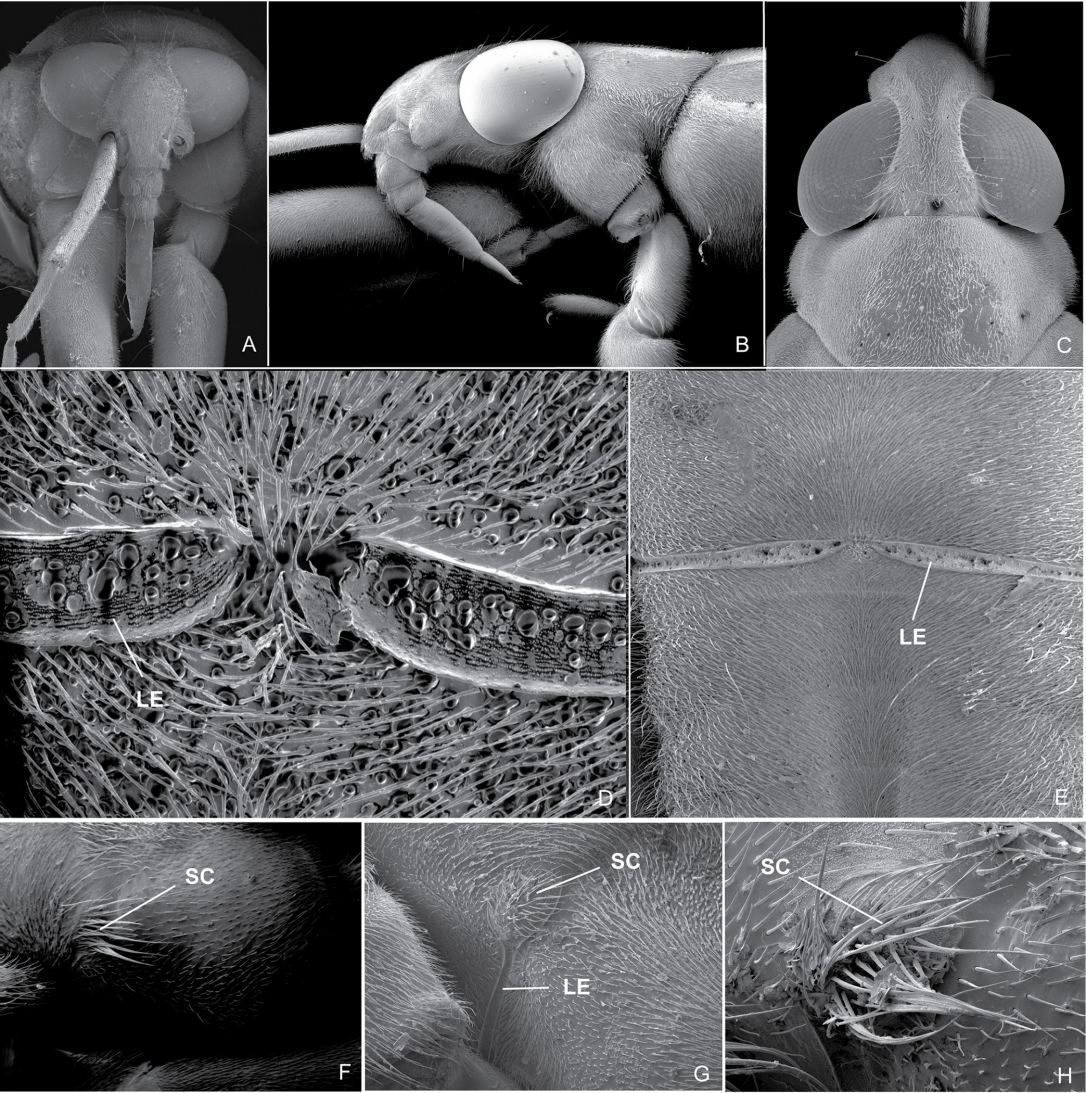
Scanning electron micrographs. (A) *P*. *sumaco*, head, frontal view; (B) *B*. *thomasi* **comb. Nov.**, head, lateral view; (C) *P*. *unidentatus*, head, dorsal; (D) *P*. *shuar*, scent groove, ventral view; (E) *P*. *osborni*, scent groove, ventral view; (F) *B*. *thomasi* **comb. Nov.**, lateral evaporatorium of metathoracic scent apparatus, lateral view; (G) *P*. *sumaco*, lateral evaporatorium of metathoracic scent apparatus, lateral view; (H) *P*. *osborni*, lateral evaporatorium of metathoracic scent apparatus, lateral view. **SC:** scent groove; **LE:** lateral evaporatorium of metathoracic scent apparatus.

2’–Mesonotum without transversal C-shaped stripe formed by silvery setae; omphalium, lateral groove, and lateral evaporatorium of scent apparatus conspicuous (Fig 5D–5H) …… *Potamobates*

3– Projection of male last abdominal laterotergite (= connexival spine) with acute apex; posterior margin of female abdominal sternum VII without projections … *Cylindrostethus*

**Fig 6:**
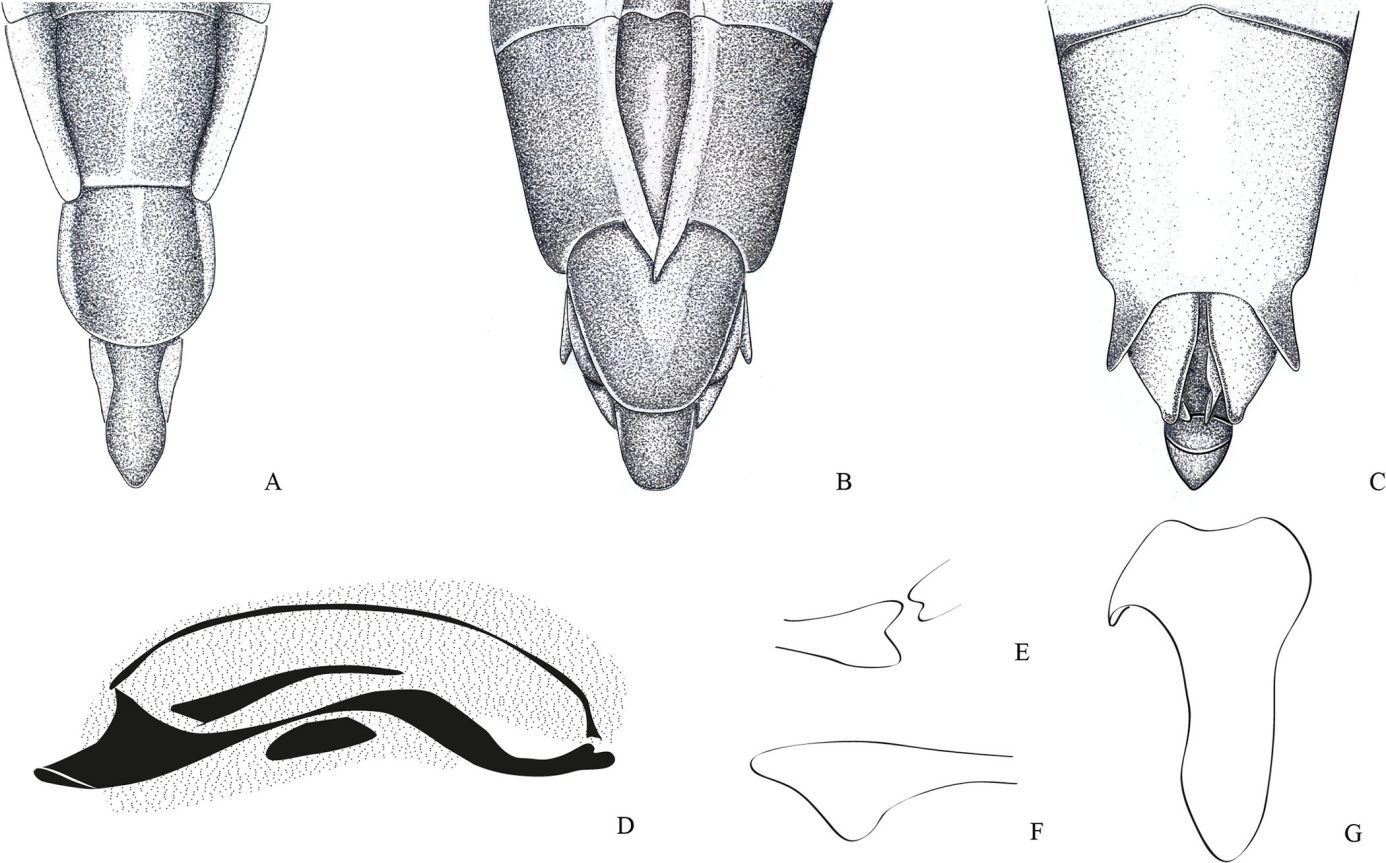
*Brailovskybates thomasi*. new comb. (A) Male, terminalia, ventral view; (B) female, terminalia, ventral view; (C) female, terminalia, ventral view; (D) phallus, lateral view; (E) junction between dorsal and ventral sclerite; (F) base ventral sclerite; (G) proctiger, dorsal view.

3’–Projection of male last abdominal laterotergite (= connexival spine) with rounded apex (Fig 6A); posterior margin of female abdominal sternum VII with a pair of lateral projections (Fig 6A) …. *Brailovskybates* Floriano and Moreira, **gen. nov.**

### *Potamobates* Champion 1898

*Potamobates* Champion, 1898: 154 (description). Drake and Harris (1934) [21]: 223–225 (key, redescription). Kuitert (1942) [17]: 139, 140 (key, redescription). Matsuda (1960) [2]: 228–231 (redescription). Polhemus and Polhemus (1995) [6]: 364–368, 370 (key, phylogeny). Cognato (1998) [18]: 21, 22 (phylogeny). Buzzetti (2006) [19]: 55, 56 (phylogeny). Padilla-Gil and Damgaard (2011) [7]: 44–49 (key, phylogeny).

**Fig 7:**
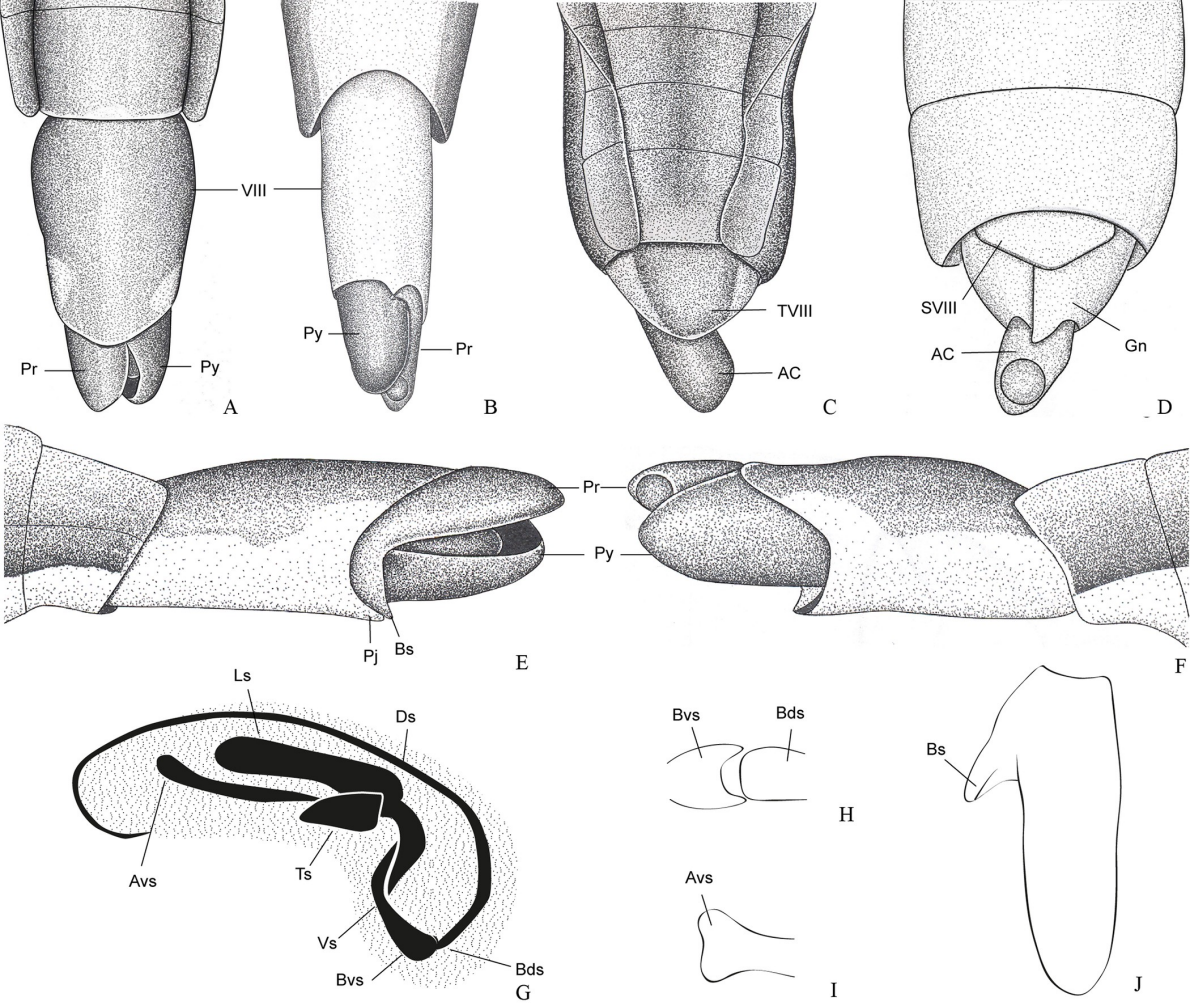
*Potamobates horvathi*. (A) Male, terminalia, dorsal view; (B) male, terminalia, ventral view; (C) female, terminalia, dorsal view; (D) female, terminalia, ventral view (E) male, terminalia, left lateral view; (F) male, terminalia, right lateral view; (G) phallus, lateral view; (H) junction between dorsal and ventral sclerite; (I) base of ventral sclarite; (J) proctiger, dorsal view.

**Fig 8:**
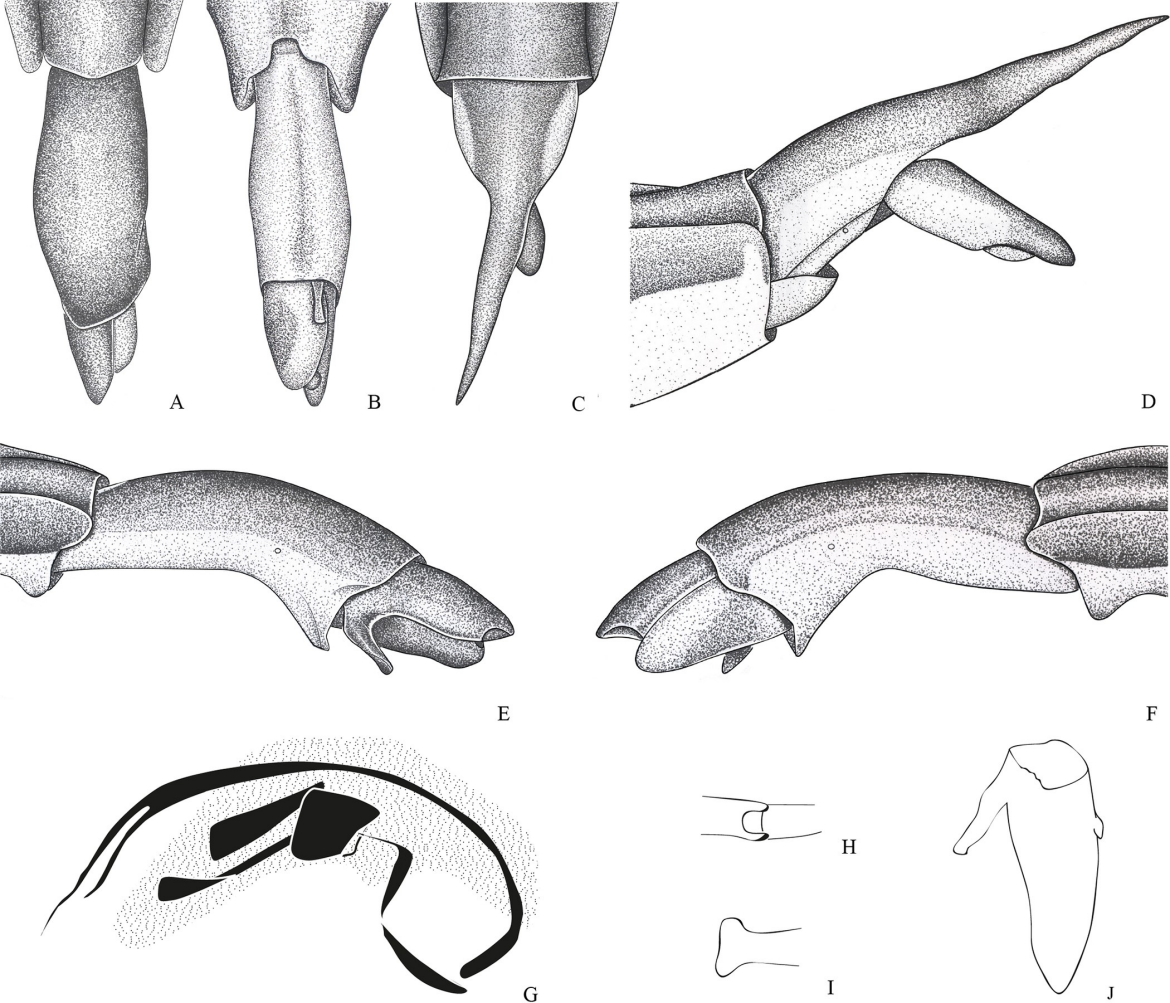
*Potamobates osborni*. (A) Male, terminalia, dorsal view; (B) male, terminalia, ventral view; (C) female, terminalia, dorsal view; (D) female, terminalia, left lateral view; (E) male, terminalia, left lateral view; (F) male, terminalia, right lateral vire; (G) phallus, lateral view (H) junction between dorsal and ventral sclerite; (I) base of ventral sclerite; (J) proctiger, dorsal view.

**Fig 9:**
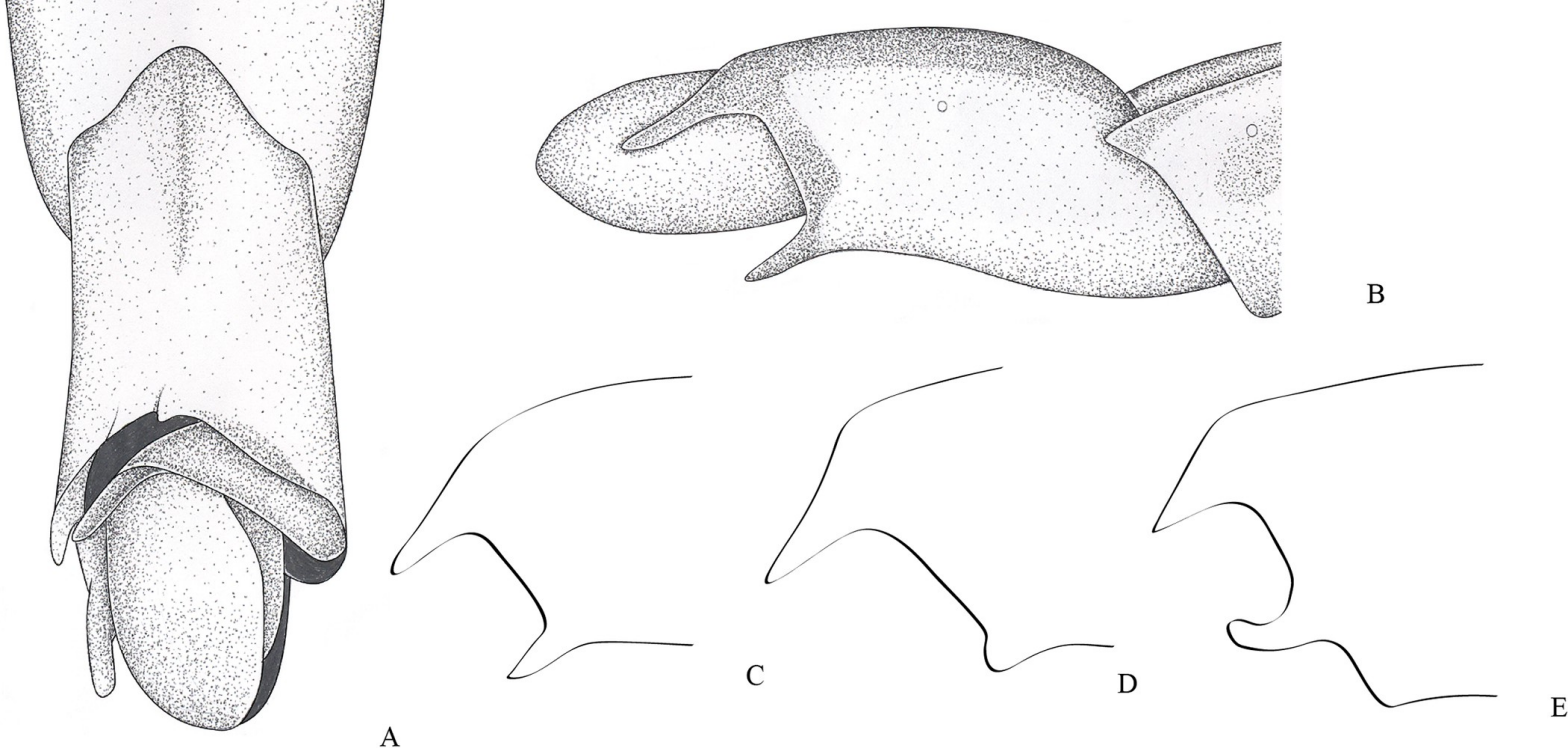
*Potamobates sumaco*. (A) Male, terminalia, dorsal view; (B) male, terminalia, right lateral view; (C-E) Variation of projections on the right posterolateral margin of male abdominal segment.

**Fig 10:**
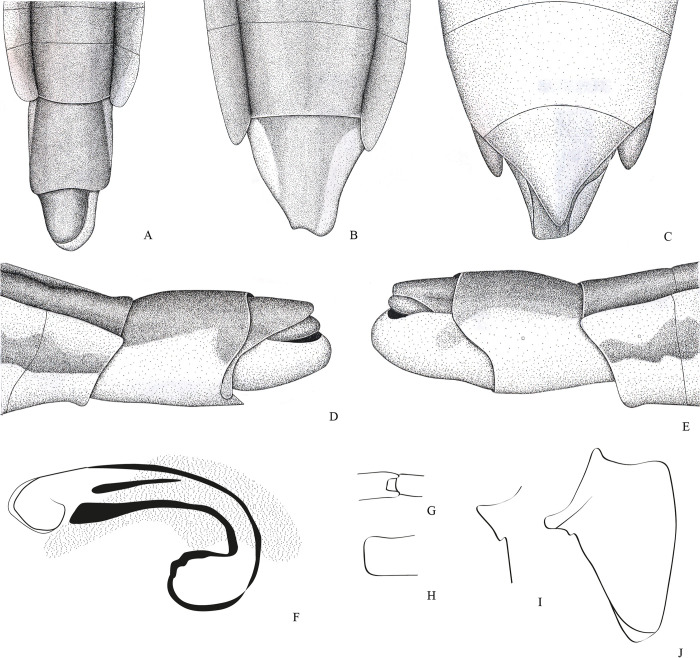
*Potamobates unidentatus*. (A) Male, terminalia, dorsal view; (B) female, terminalia, dorsal view; (C) female, terminalia, ventral view; (D) male, terminalia, left lateral view; (E) male, terminalia, right lateral view; (F) phallus, lateral view; (G) junction between dorsal and ventral sclerite; (H) base of ventral sclerite; (I) proctiger, left basolateral process; (J) proctiger, dorsal view.

**Fig 11:**
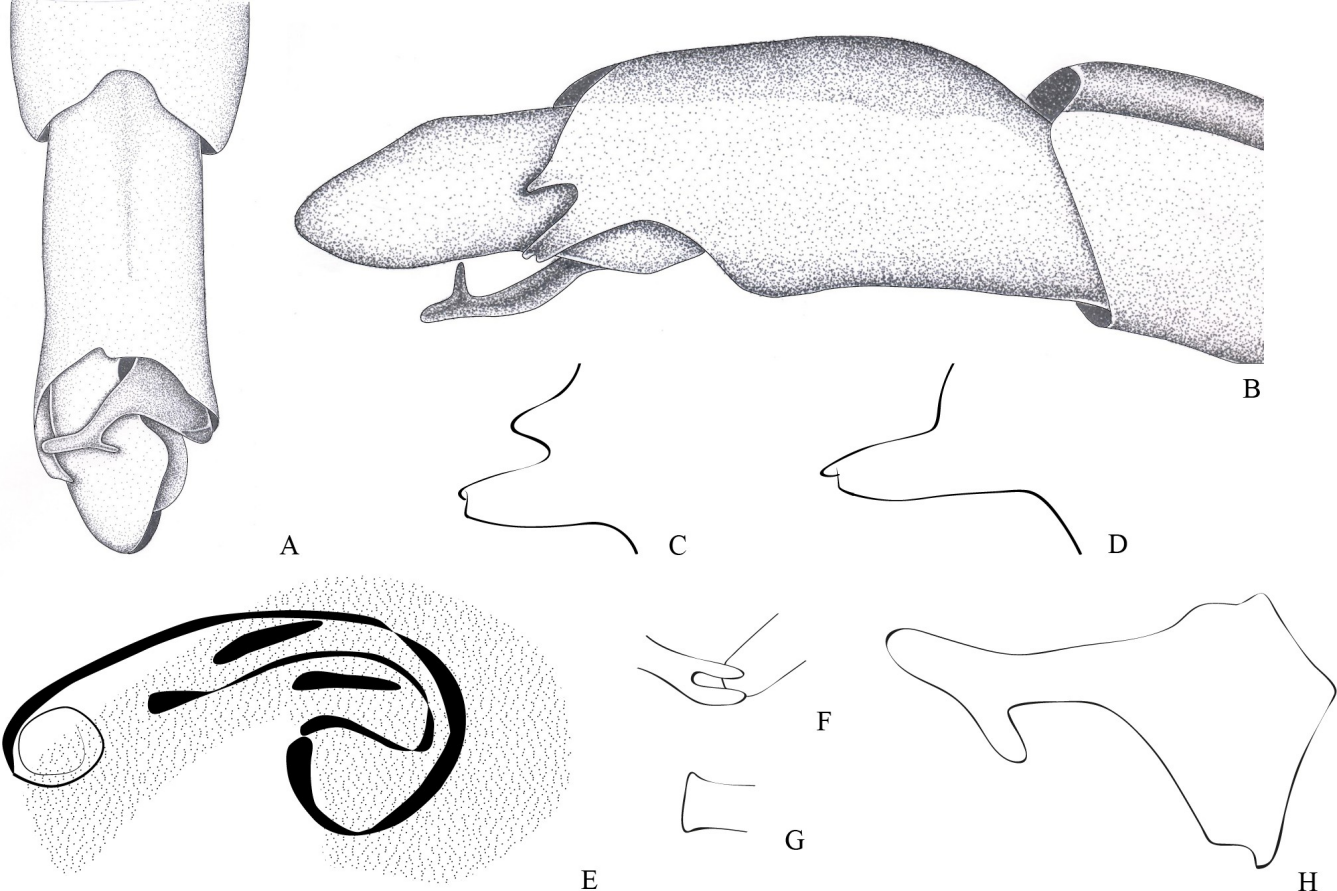
*Potamobates variabilis*. (A) Male, terminalia, ventral view; (B) male, terminalia, right lateral view; (C-D) Variation of projections on the right posterolateral margin of male abdominal segment; (E) phallus, lateral view; (F) junction between dorsal and ventral sclerite; (G) base ventral sclerite; (H) proctiger, dorsal view.

**Fig 12:**
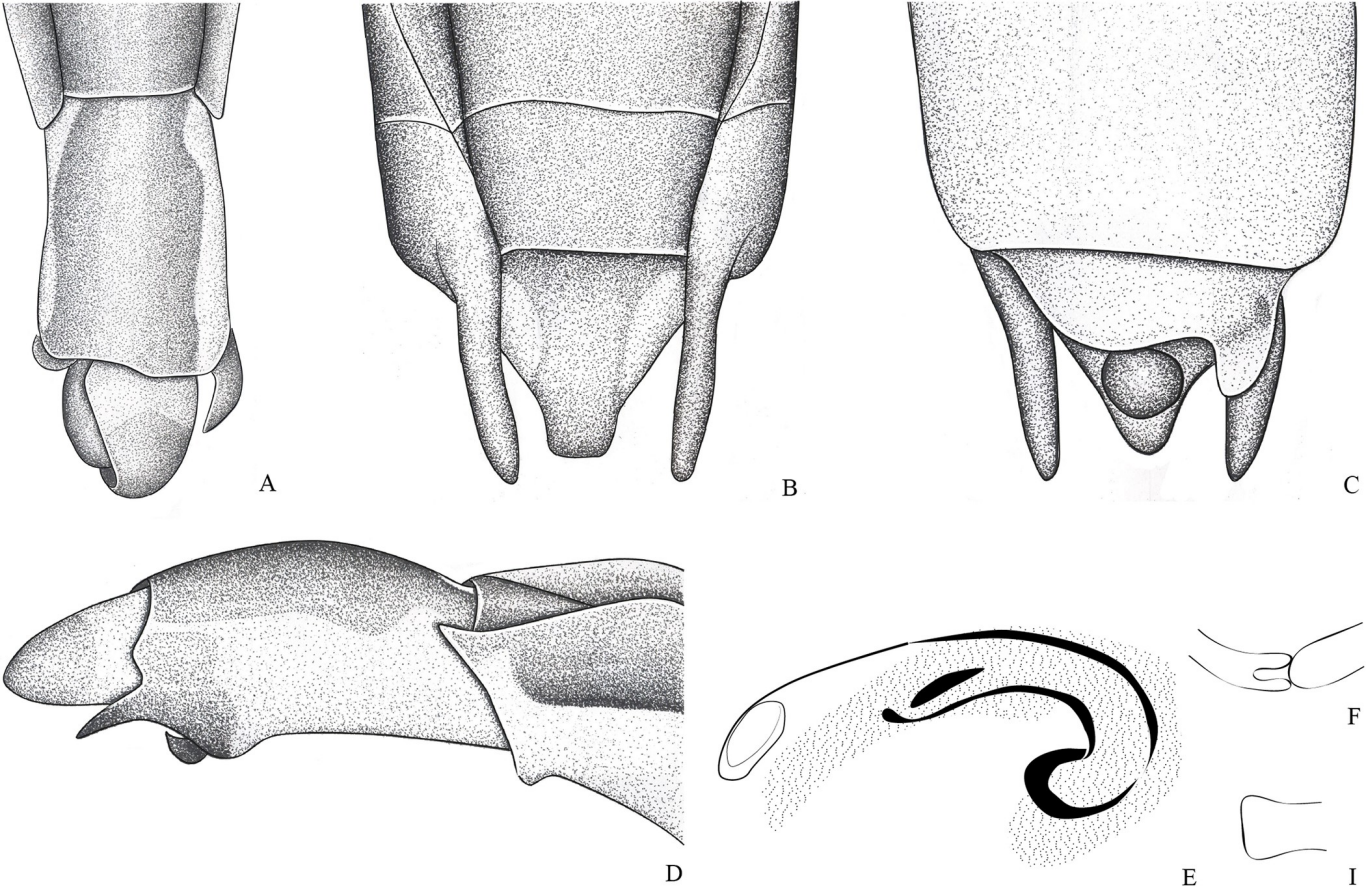
*Potamobates peruvianus*. (A) Male, terminalia, dorsal view; (B) female, terminalia, dorsal; (C) female, terminalia ventral view; (D) male, terminalia, right lateral view; (E) phallus, lateral view; (F) junction between dorsal and ventral sclerite; (G) base of ventral sclerite.

**Diagnosis.** Mandibular and maxillary plates fused (Fig 5B); epistomal suture absent (Fig 5C); labium short, reaching at most anterior portion of mesosternum (Fig 5B); middle and hind pretarsal claws absent; abdomen short. *Male*: posterior projection of last abdominal laterotergite (= connexival spine) with at most 1/4 of length of abdominal tergum VIII (Figs 1A, 3A, 7A and 8A); abdominal segment VIII usually with one or more projections; pygophore and proctiger sinistrally rotated (Figs 1A, 1B, 3A, 3B, 3E, 7A, 7B and 8A, 8E and 9A); right basolateral process of proctiger reduced or absent; left basolateral process elongated or triangular (Figs 2F, 7J, 8J, 10J and 11H). *Female*: posterior margin of abdominal sternum VII produced posteriorly with varying lengths and shapes, without pair of lateral projections (Figs 1D, 3D, 7D and 12C).

#### Redescription

**Fig 13:**
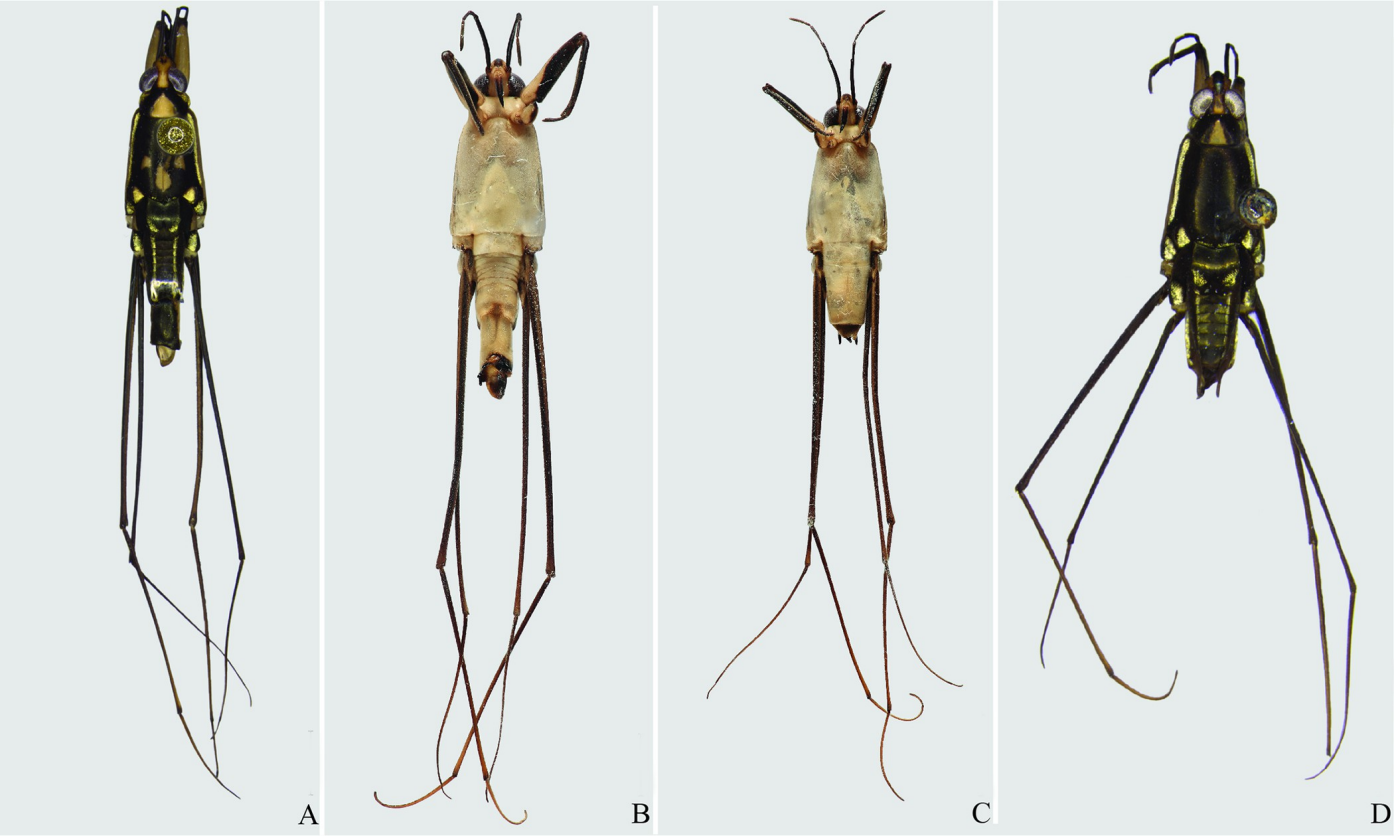
*Potamobates anchicaya*. (A) Male, dorsal view; (B) male, ventral view; (C) female, dorsal view; (D) female, ventral view.

**Fig 14:**
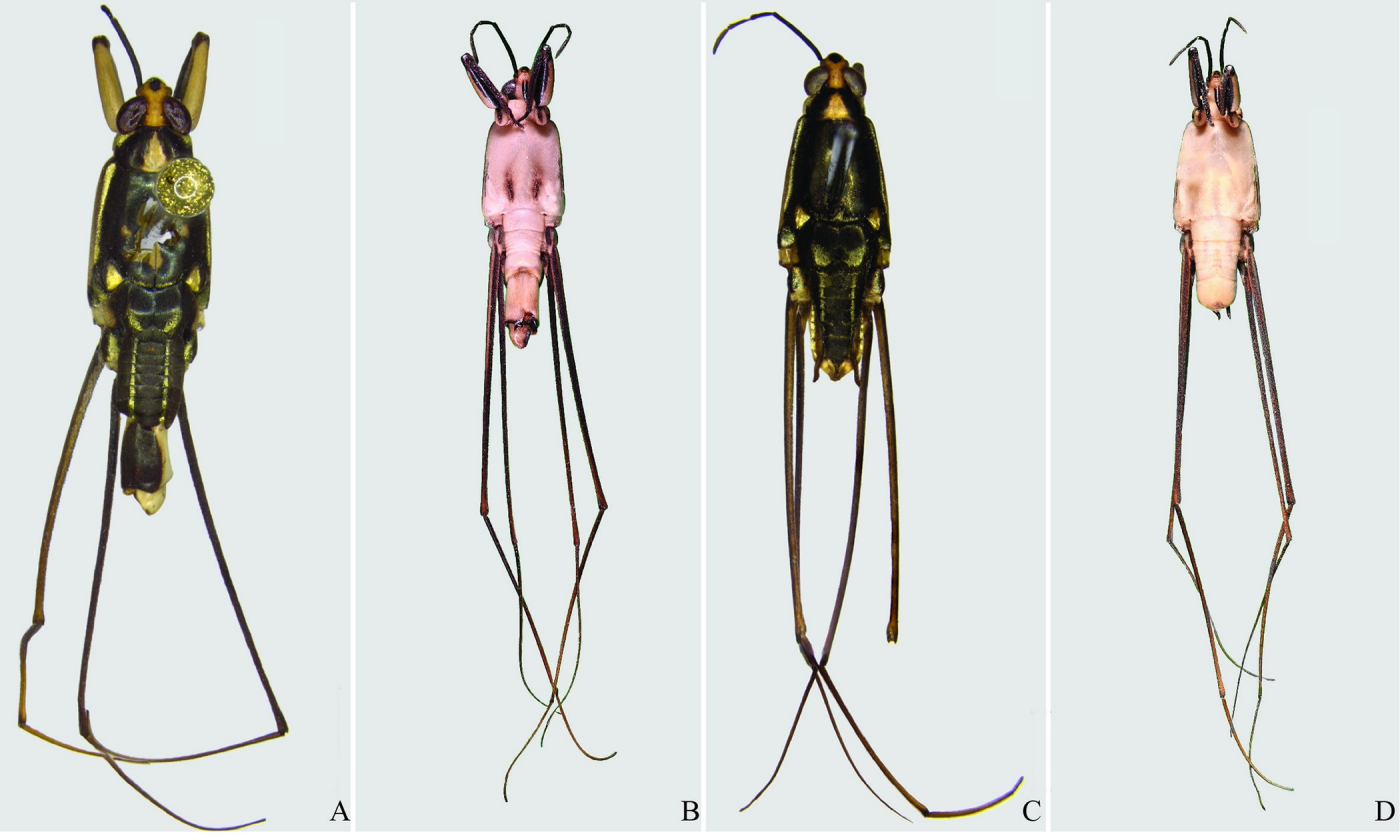
*Potamobates carvalhoi*. (A) Male, dorsal view; (B) male, ventral view; (C) female, dorsal view; (D) female, ventral view.

**Fig 15:**
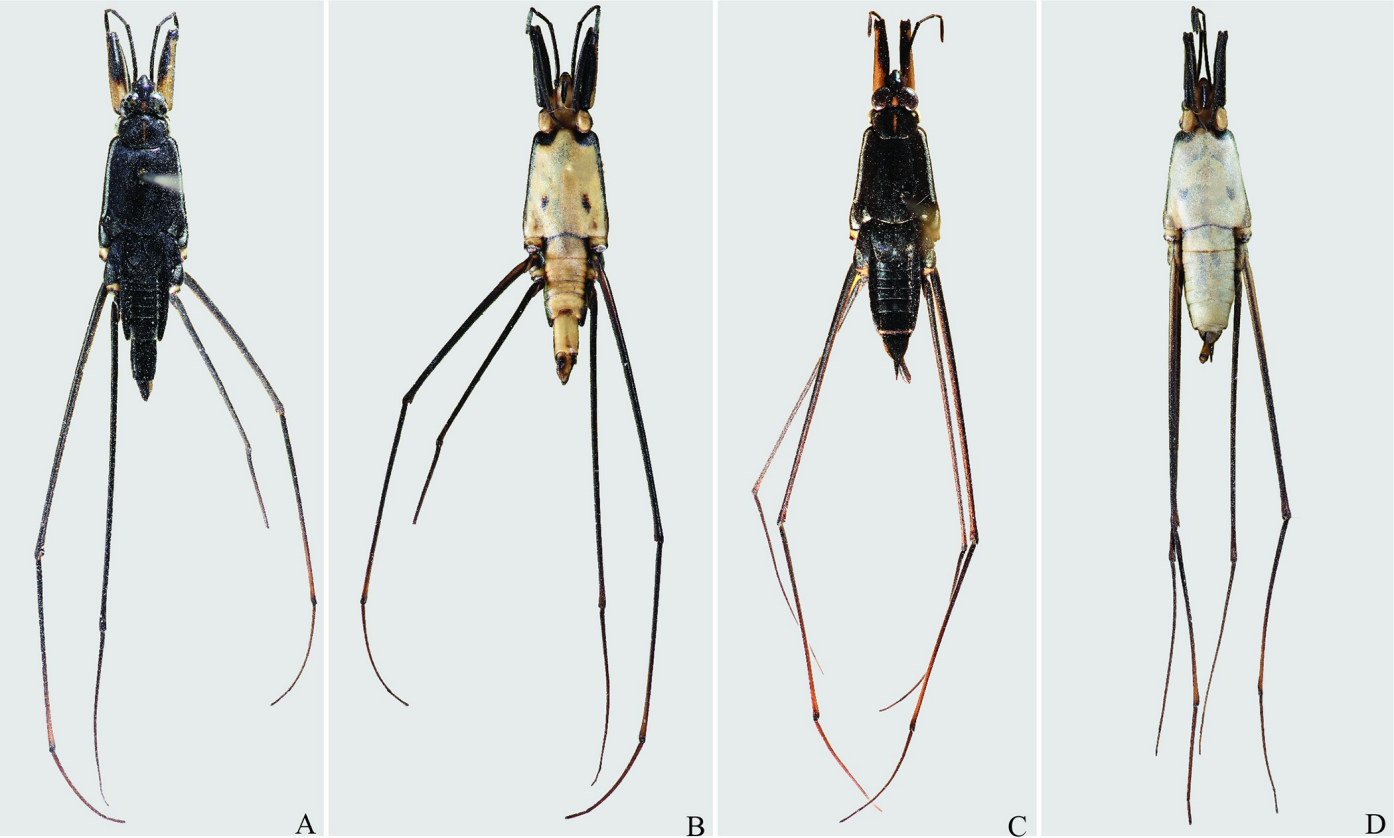
*Potamobates bidentatus*. (A) Male, dorsal view; (B) male, ventral view; (C) female, dorsal view; (D) female, ventral view.

**Fig 16:**
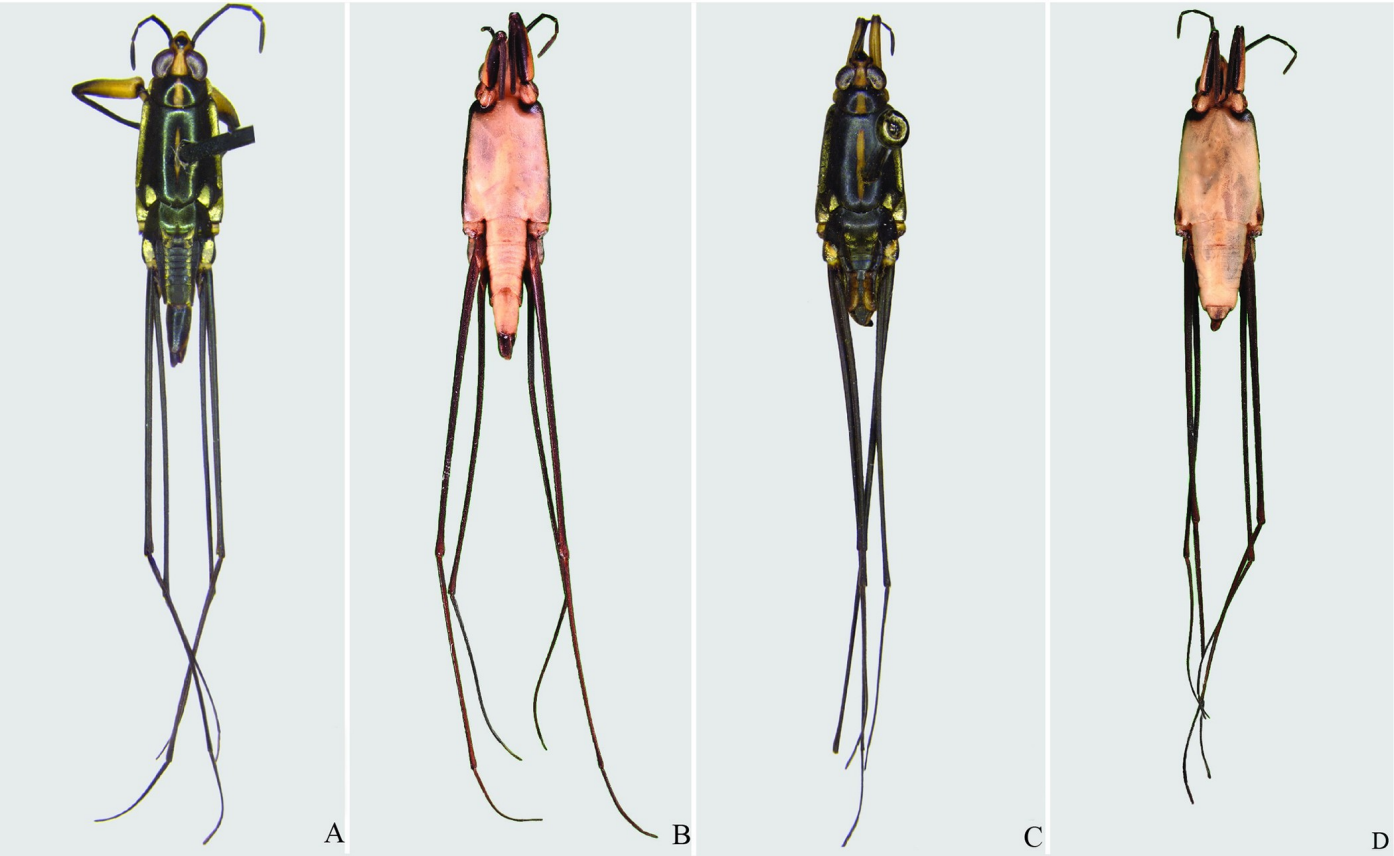
*Potamobates horvathi*. (A) Male, dorsal view; (B) male, ventral view; (C) female, dorsal view; (D) female, ventral view.

**Fig 17:**
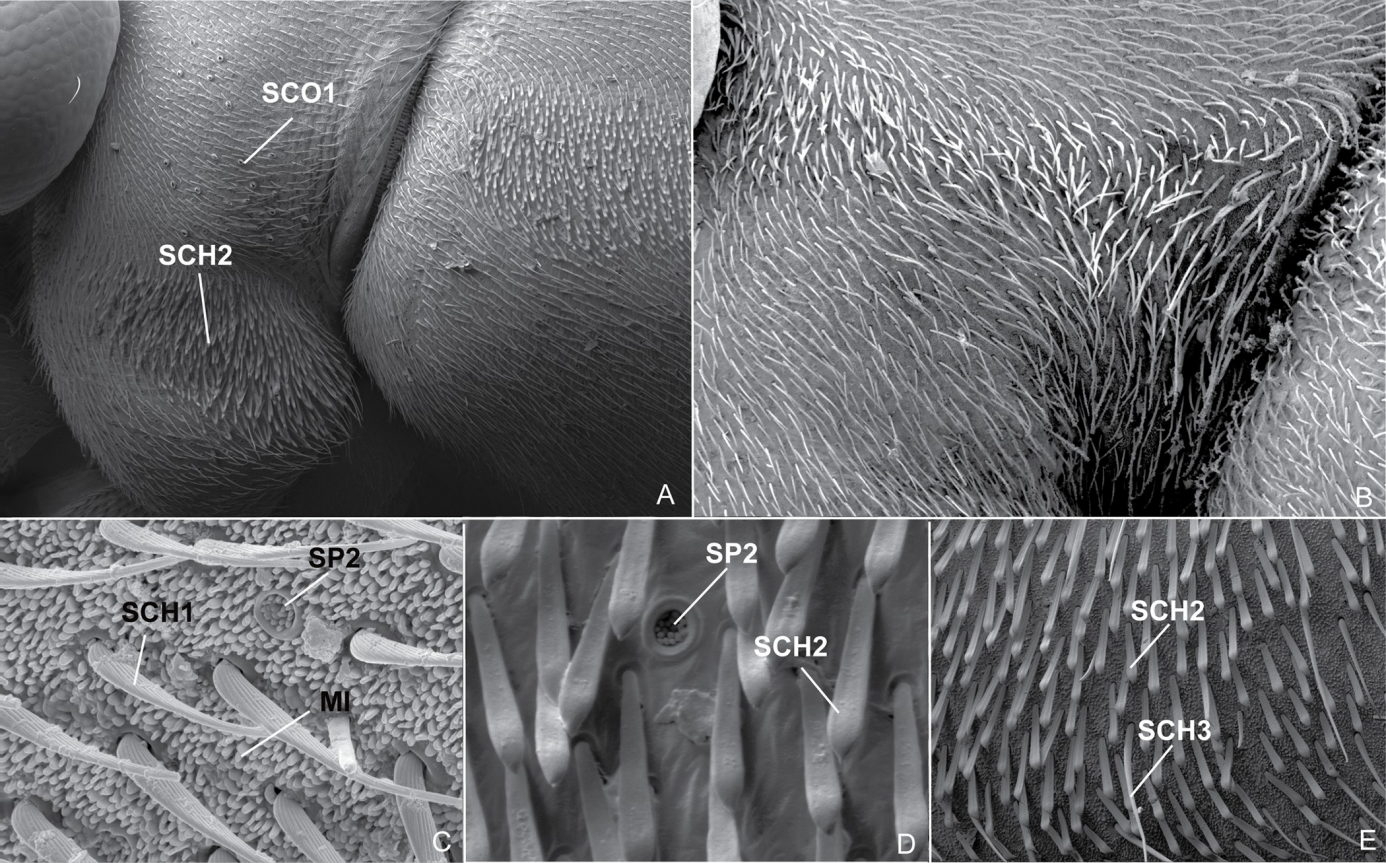
Scanning electron micrographs. (A) *P*. *horvathi*, propleurum, vista lateral; (B) *B*. *thomasi* **comb. Nov.**, propleuron, lateral view; (C) *P*. *unidentatus*, head, dorsal view; (D) *P*. *horvathi*, mesopleuron, lateral view; (E) *P*. *shuar*, propleuron, lateral view. **MI:** Microtrichia; **SCH1:** Sensilla chaetica 1; **SCH2:** Sensilla chaetica 2; **SCH3**: Sensilla chaetica 3; **SCO1:** Sensilla coeloconica 1; **SP2** Sensilla placodea 2.

**Fig 18:**
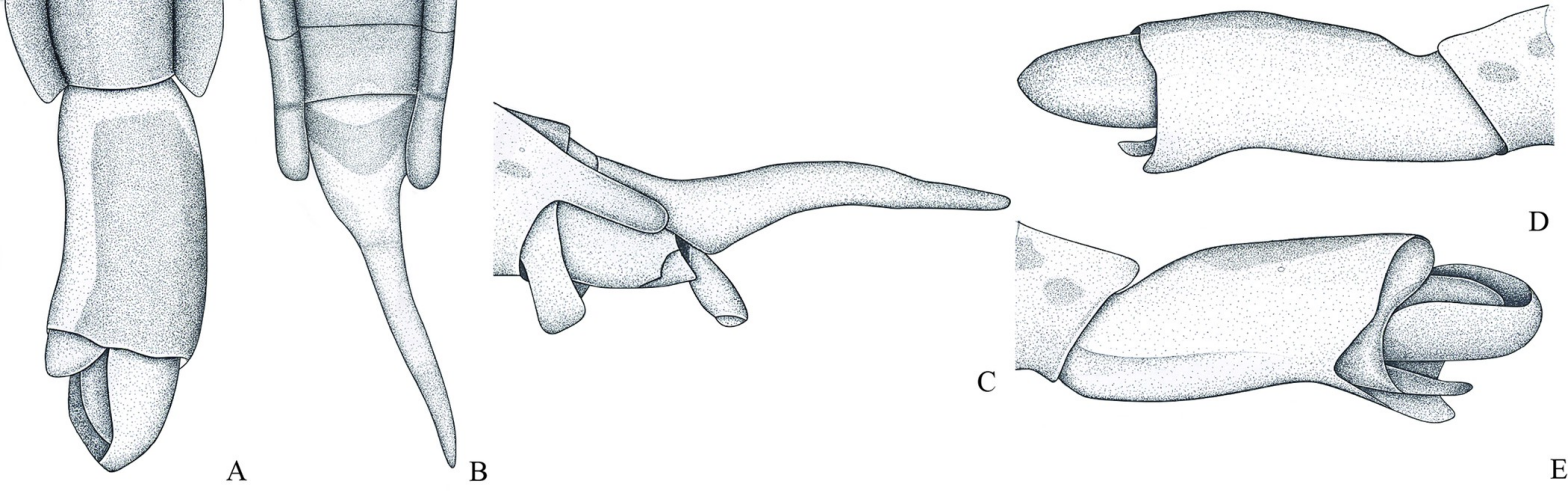
*Potamobates molanoi* sp. new. (A) Male, terminalia, dorsal view; (B) female, terminalia, dorsal view; (C) female, terminalia, lateral view; (D) male, terminalia, right lateral view; (E) male, terminaia, left lateral view.

**Fig 19:**
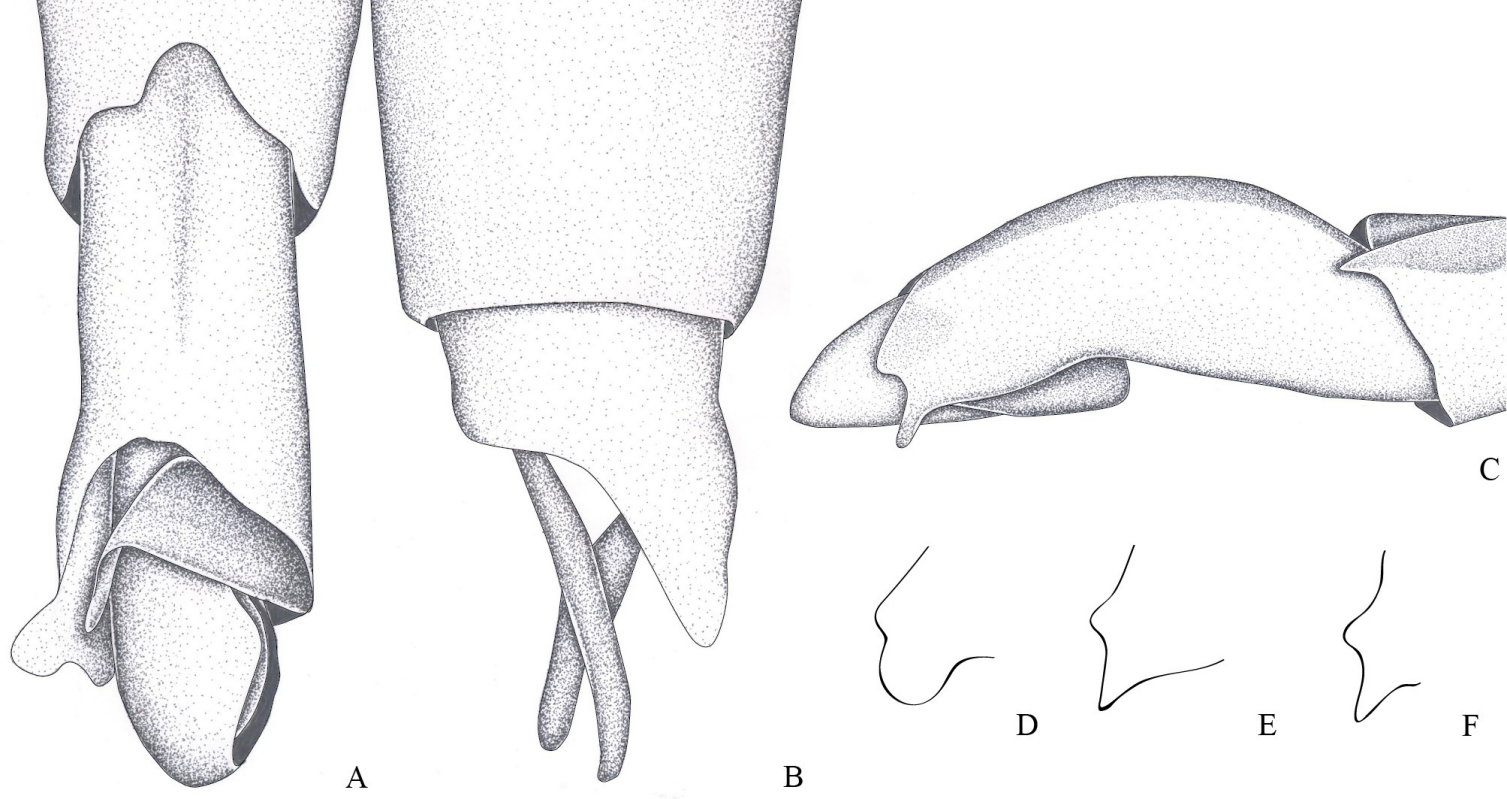
*Potamboates woytkowskii*. (A) Male, terminalia, ventral view; (B) female, terminalia, ventral view; (D) male, terminalia, left lateral view; (F) male, terminalia, right lateral virw; (G) phallus, lateral view; (H) junction between dorsal and ventral sclerite; (I) base of ventral sclerite; (J) proctiger, dorsal view.

**Fig 20:**
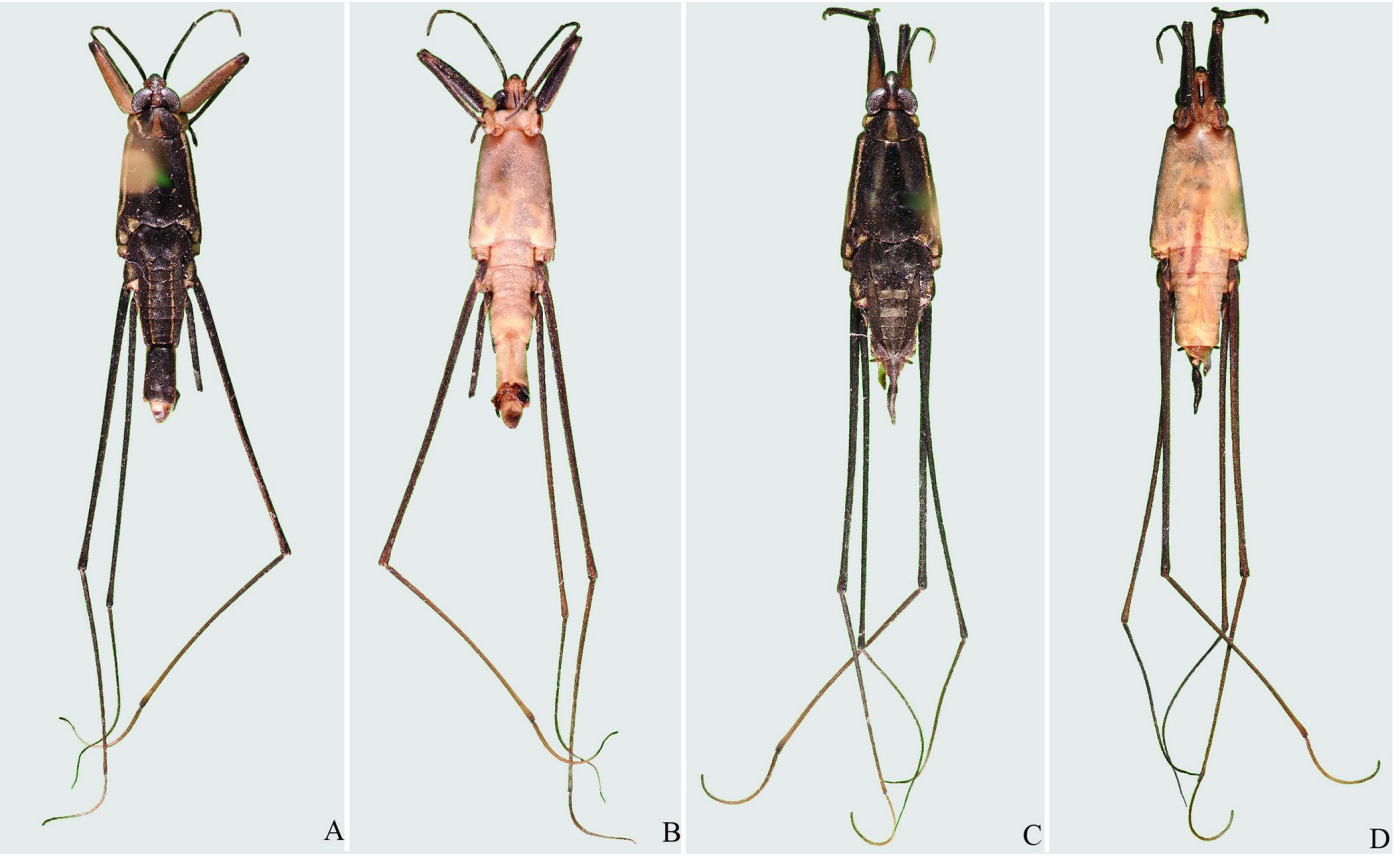
*Potamobates spiculus*. (A) Male, dorsal view; (B) male, ventral view; (C) female, dorsal view; (D) female, ventral view.

Body length 3.3–4.8 times larger than width through mesoacetabula. *Head*: width through eyes 1.0–1.6 times larger than head length. Eye reniform, greatest width 1.3–2.0 times larger than minimum interocular distance. Mandibular and maxillary plates fused (Fig 5B). Clypeus rounded anteriorly (Fig 5A); epistomal suture absent (Fig 5C). Antenniferous tubercle protuberant. Antenna short, subequal in length to fore leg; antennomere I 2.3–3.1 times longer than II, 0.7 times of length of II + III; II from subequal in length to 1.7 times longer than III; IV subequal to 1.5 times longer than III. Labium short, reaching at most anterior portion of mesosternum (Fig 5B); articles I and II short; III 1.5–2.3 times longer than IV (Fig 5B). *Thorax*: pronotum with a median stripe (Figs 13A, 13D, 14A and 14C) or wedge-shaped mark (Figs 15A, 15C, 16A and 16C); propleuron with an anterior patch of golden setae (except in *P*. *horvathi*); proacetabulum with a patch or wide stripe of golden setae (Fig 17A and 17B). Fore femur subequal in length to fore tibia, proximal region with denticles; fore tarsomere II 2.2–5.0 times longer than I. Mesonotum with median length subequal to width through mesoacetabula, uniformly black or with yellowish longitudinal stripe(s); mesopleuron with a continuous or interrupted stripe of golden setae. Middle femur with 0.9–1.0 of length of hind femur, 1.4–1.7 times longer than middle tibia; middle tarsomere I 3.0–6.0 times longer than II; middle pretarsal claws absent. Metanotum uniformly black or with a median yellowish stripe, width through acetabula 2.5–3.5 times median length; metapleuron with a patch or longitudinal stripe of golden setae. Hind femur 1.6–2.1 times longer than hind tibia; hind tarsomere I 1.3–2.1 times longer than II; hind pretarsal claws absent. Metathoracic scent apparatus well developed, with lateral channels and hair tuft (Fig 5D–5H). *Abdomen*: subequal in length or shorter than mesothorax, dorsum with or without yellowish median stripe; lateral margins of mediotergites with stripe of golden setae; spiracles located closer to posterior than to anterior margins of segments. Mediotergite I cordiform, 1.0–1.6 times longer than II; II with 1.1–1.5 times longer than III; III–V subequal in length. *Male*: posterior projection of last abdominal laterotergite (= connexival spine) with at most 1/4 of length of abdominal tergum VIII (Figs 1A, 3A, 7A and 8A). Posterior margin of abdominal sternum VII with a central notch (Figs 1B, 3B, 8B and 9A). Abdominal segment VIII dorsally at least about two times longer than wide; ventral or right margin usually with one or more projections (Figs 1G, 2A, 3E, 3F, 7B, 8E and 12D). Pygophore and proctiger sinistrally rotated (Figs 1A, 1B, 3A, 3B, 3E, 7A, 7B, 8A, 8E and 9A). Proctiger asymmetrical; right basolateral process reduced or absent (Figs 2F, 7J, 8J and 10J); left basolateral process elongated or triangular (Figs 2F, 7J, 8J, 10J and 11H). Parameres reduced. Phallus with dorsal sclerite sclerotized and wide (Figs 2A, 7G, 8G, 10F, 11E and 12E); base of ventral sclerite bifid, with projections (Figs 2D, 7H, 8H, 10G, 11H and 12F). *Female*: posterior projection of last abdominal laterotergite (= connexival spine) present, except in *P*. *bidentatus*, *P*. *horvathi*, *P*. *manzanoae*, and *P*. *osborni* (Figs 1C, 3C, 7C, 8C and 18B); posterior margin of abdominal sternum VII produced posteriorly with varying lengths and shapes, without pair of lateral projections (Figs 1D, 3D, 7D, 10C, 12C and 19B). Abdominal tergum VIII triangular, sometimes elongated posteriorly (Figs 8C, 15C, 18B and 20C).

#### Comments

*Potamobates* differs from *Cylindrostethus* by the shorter body, 7–12 mm vs. 8–26 mm, at most five times longer than wide; the abdominal spiracles located closer to the posterior margins of the segments than to the anterior margins; the abdomen at most subequal to the mesonotum in length; the projection of the last abdominal laterotergite (= connexival spine) of the male reduced or absent; the male abdominal segment VIII with projections, dorsally about two times longer than wide; the male pygophore and proctiger sinistrally rotated; the left margin of the male proctiger folded ventrally; the male phallus elongated; the female with the posterior margin of the abdominal tergum VIII acute; and the posterior margin of female abdominal sternum VII produced posteriorly with varying lengths and shapes. *Potamobates* can be easily separated from *Platygerris* based on the tubular body, the longer abdomen, and the presence of the opening of the metathoracic scent apparatus.

Of all species currently included in *Potamobates*, only *Po*. *thomasi* does not display the diagnostic features of the genus, neither does it fit into the other two genera of Cylindrostethinae. Furthermore, it resulted as sister to (*Platygerris* + other *Potamobates*) in a recent phylogenetic analysis of Cylidrostethinae [20]. In earlier studies of the relationships within *Potamobates* [6, 7, 18, 19], this species was always recovered as sister to all other congeners, but only *Cylindrostethus* had been used as outgroup. Floriano (2017) [20], in turn, was the first to include a full set of Cylindrostethinae genera in a phylogenetic analysis, using other taxa of Gerromorpha as outgroups. Considering this information, we describe a new genus in the subfamily for *Po*. *thomasi* and further discuss it at the end of this revision.

Andersen (1982) [22] recorded a series of reductions in the evaporatory apparatus of the scent gland in Cylindrostethinae. The lateral channels and tufts of setae were present in *Cylindrostethus*, while in *Potamobates* the lateral channels would disappear before reaching the metepisternum and there would be not tufts of setae. However, our own study of *Potamobates* revealed, in the species examined, that the lateral channels reach the metepisternum and that there are tufts of setae, although not as developed as in *Cylindrostethus* (Fig 5D–5H).

The sensory system of insects consists of a large number of highly diverse organs called sensilla [23], which are small cuticular sensory organs consisting of three structural elements: sensory cells, enveloping cells, and a cuticular structure [24]. According to Shields (2010) [25], the sensillum types have been classified on the basis of the morphology of their cuticular parts, as well as the location on the insect. In *Potamobates*, we found the following types: sensilla trichoidea, sensilla chaetica, sensilla basiconica, sensilla coeloconica, sensilla ampullacea, sensilla campaniformia, and sensilla placodea.

**Fig 21:**
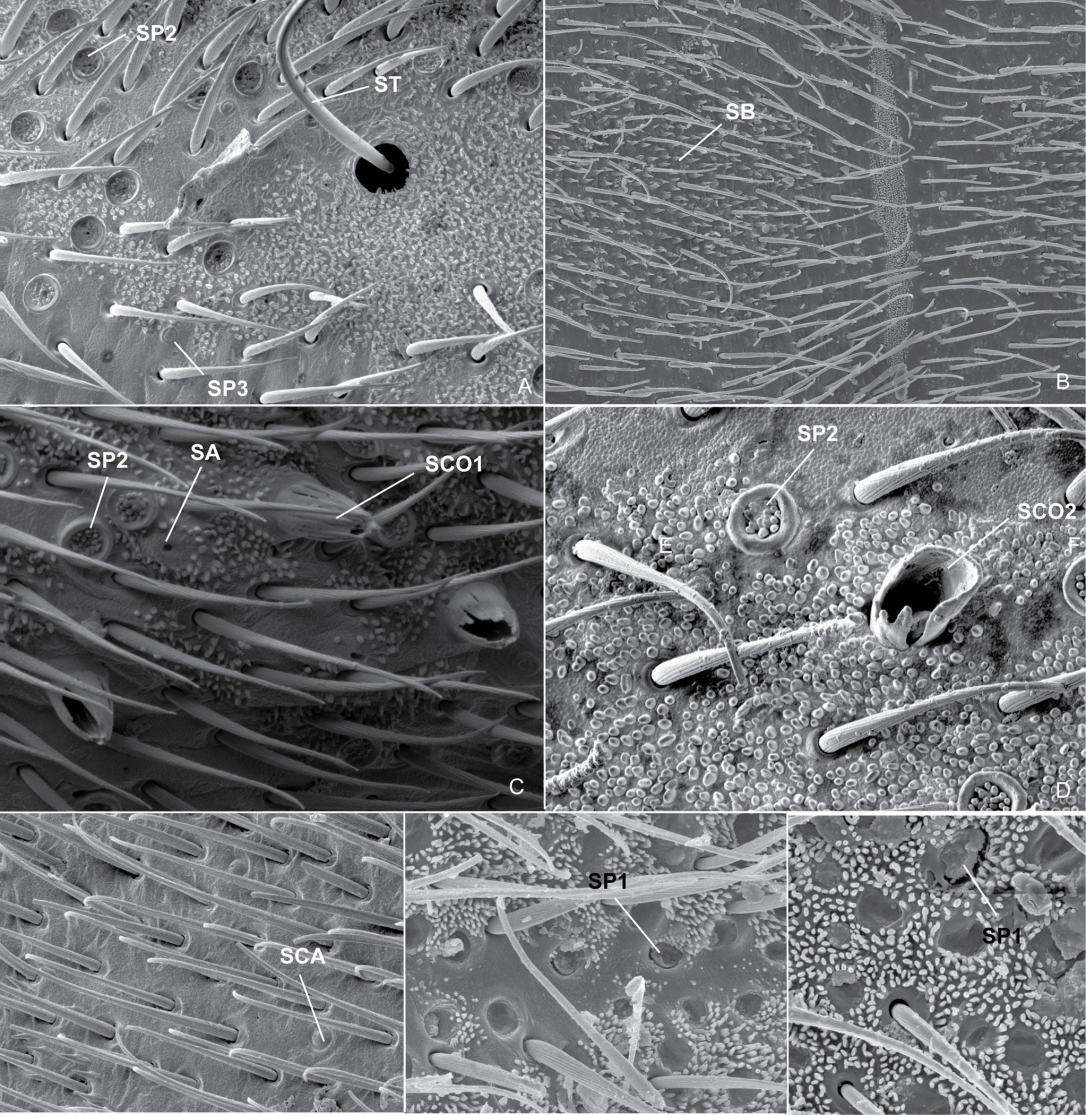
Scanning electron micrographs. (A) *P*. *horvathi*, head, dorsal view; (B) *P*. *unidentatus*, abdominal mesotergites II-III, dorsal view; (C) *P*. *horvathi*, propleuron, lateral view; (D) *P*. *unidentatus*, propleuron, lateral view; (E) *P*. *shuar*, mesotergite VII, dorsal view; (F) *P*. *unidentatus*, mesonotum, dorsal view; (G) *P*. *unidentatus*, head, dorsal view. **SA**: Sensilla ampullacea; **SB**: Sensilla basiconica; **SCA**: Sensilla campaniformia; **SCO1**: Sensilla coeloconica 1; **SCO2:** Sensilla coeloconica 2; **SP1**: Sensilla placodea 1; **SP2**: Sensilla placodea 2; **SP3**: Sensilla placodea 1; **ST:** Sensilla trichoidea.

Sensilla trichoidea (ST) vary greatly in length and are freely moveable [25]. They are usually called trichobothria in Heteroptera literature and are found in pairs on the head of gerromorphans, including *Potamobates* (Fig 21A). Sensilla chaetica (SCH) are similar to ST, but have thicker cuticular shafts and are not freely moveable [25]. We found three types of SCH in *Potamobates*: SCH1, dispersed through all the body, with the base rounded and the lateral margins converging distally, sometimes curved at the apex (Fig 17); SCH2, present mainly in the acetabula, with the base rounded and the apex elongated (Fig 17E); and SCH3, found in the same spots as SCH2, flattened and with a slight central sulcus, with the lateral margins diverging distally and the apex apruptly convergent (Fig 17A and 17D).

Sensilla basiconica (SB) are short setae (pegs) [25]. We found them on the abdominal mediotergites of *Potamobates*, and they are distinctly shorter than other sensilla (Fig 21B). Sensilla campaniformia (SCA) are dome or bell-shaped, positioned at or below the cuticular surface [25]. We found them only on the abdominal mediotergites of *Po*. *shuar* (Fig 21E), but Nowińska and Brożek (2017) [23] reported this type in different species of Gerridae and Veliidae. Sensilla ampullacea (SA) are pegs similar to coeloconic sensilla in structure and function, but are positioned in deep pits with narrow openings [25]. We found them on the propleuron and the dorsum of the head in *Potamobates* (Fig 21C), where they resemble small holes from where setae fell off.

Sensilla placodea (SP) are flat, plate-like sensilla positioned at, above, or below the cuticular surface [25]. We found three types in *Potamobates*: SP1, in depressions, with a lateral slit (Fig 21F and 21G); SP2, oval or circular depressions with minute subconical pegs within (Figs 17C, 17D, 21A, 21C and 21D); and SP3, similar to SP2, but smaller and without the central pegs (Figs 17C, 17D, 21A, 21C, 21D, 21F and 21G). Andersen (1982) [22] had already reported structures similar to SP1 in Cylindrostethinae, which according to him were restricted to areas of the body surface above the points of attachment of major muscles. Our SP2 seems to be the same structure named as peg-plates by Andersen (1977, 1982) [22, 26]. According to Cobben (1978) [27], these would be found in all Gerromorpha, except for Hermatobatidae, but Andersen (1982) [22] did not find them in gerrids. Floriano et al. (2020) [20], in turn, found them in *Platygerris*, and here we observed them both in *Potamobates* and in *Brailovskybates* Floriano and Moreira, **gen. nov.**

Finally, sensilla coeloconica (SCO) are basiconic pegs or cones that are positioned in shallow pits [25]. We found two types in *Potamobates*: SCO1, cone-shaped, with the lateral margins formed by structures similar to setae (Fig 21C); and SCO2, with the lateral margins cone-shaped (Fig 21D). Andersen (1982) [22] recorded SCO1 on the ventral surface of *Cylindrostethus*, and we found it in *Po*. *horvathi*. This type of structure is probably widespread in the subfamily. The type SCO2 was found by us only on the propleuron of *Po*. *unidentatus*.

#### Geographic distribution

This genus occurs from Mexico to Peru. In South America, it is restricted to the western portion of the continent.

**Type species.** *Potamobates unidentatus* Champion, 1898; by subsequent designation (Kirkaldy 1906: 155) [28].

### Key to the species of *Potamobates*

Adapted from Drake and Harris (1934) [21], Kuitert (1942) [17], and Polhemus and Polhemus (1995) [6]. Both male and female specimens are needed to properly run this key.

**1**– Male pygophore and proctiger sinistrally rotated up to 45° (Figs 2A, 7A, 8A, 8E and 10A) … 2

**1**’–Male pygophore and proctiger sinistrally rotated about 90° (Figs 1A, 3B, 9A, 12D and 18A)… 6

**Fig 22:**
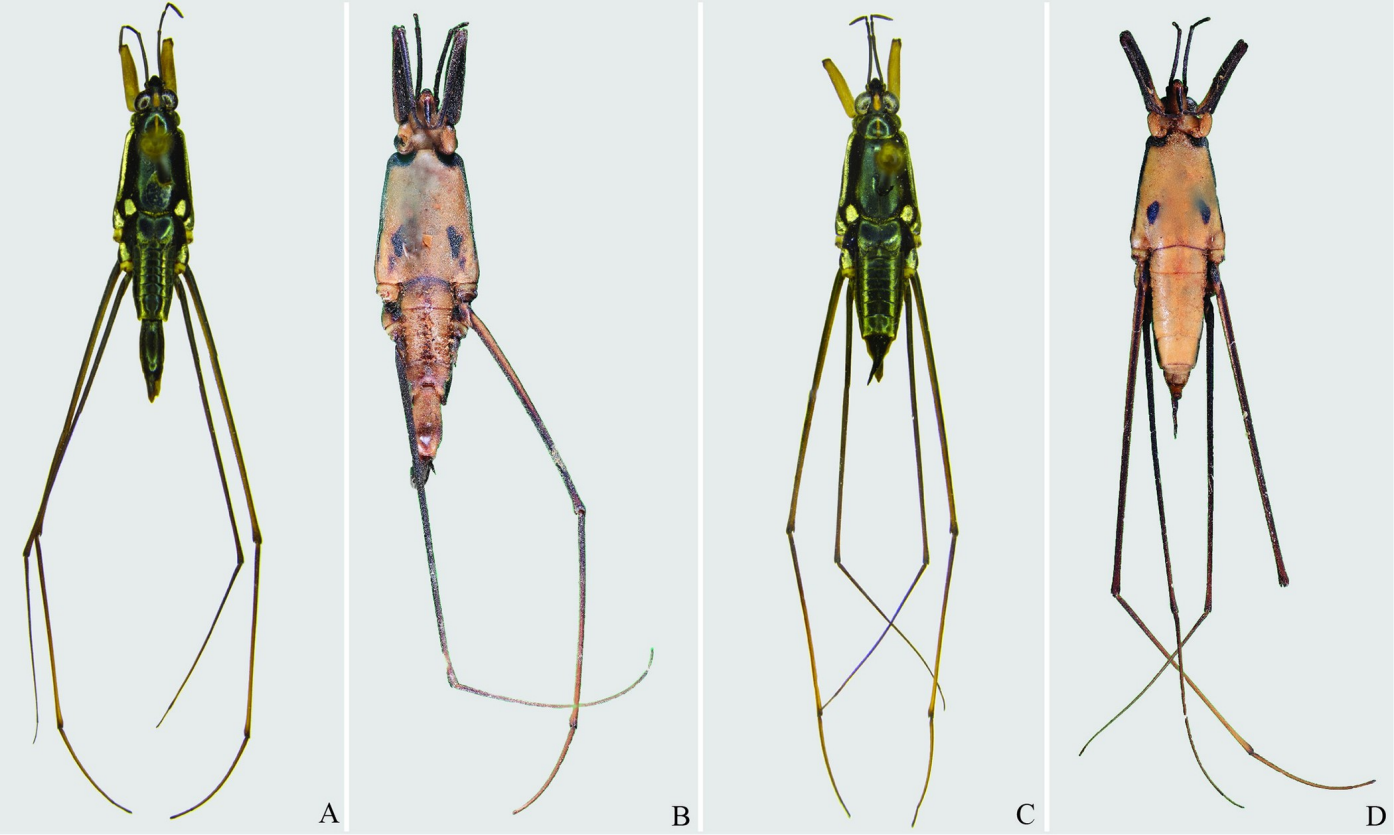
*Potamobates osborni*. (A) Male, dorsal view; (B) male, ventral view; (C) female, dorsal view; (D) female, ventral view.

**2**– Pronotum with a median stripe (Figs 15A, 15C, 16A, 16C, 22A and 22C) … 3

**Fig 23:**
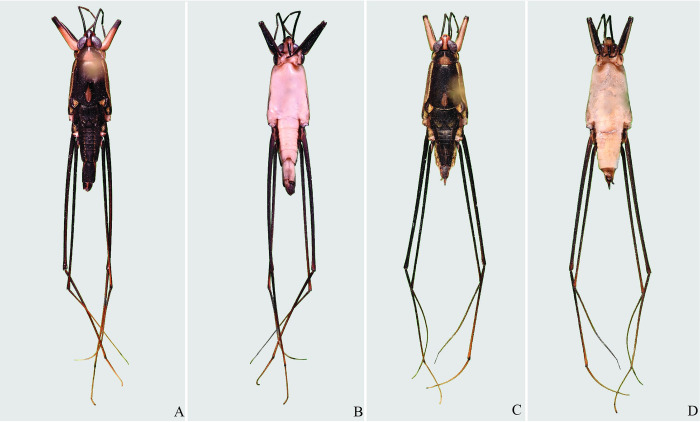
*Potamobates manzanoae*. (A) Male, dorsal view; (B) male, ventral view; (C) female, dorsal view; (D) female, ventral view. **VIII**: segment VIII; **BS**: basolateral process of proctiger; **PR:** Proctiger; **PH**: Phallus; **PY:** Pigophore; **PJ:** Projection of segment VIII; **CS**: Connexival spine; **SVIII**: Sternum VIII; **AC**: Anal cone; **GN**: Gonocoxae; **DS**: Dorsal sclerite of phallus; **LS:** lateral sclerite of phallus; **TS:** Transversal sclerite of phallus; **AVS:** Apex of ventral sclerite; **BVS**: Base of ventral sclerite; **BDS**: Base of ventral sclerite.

**Fig 24:**
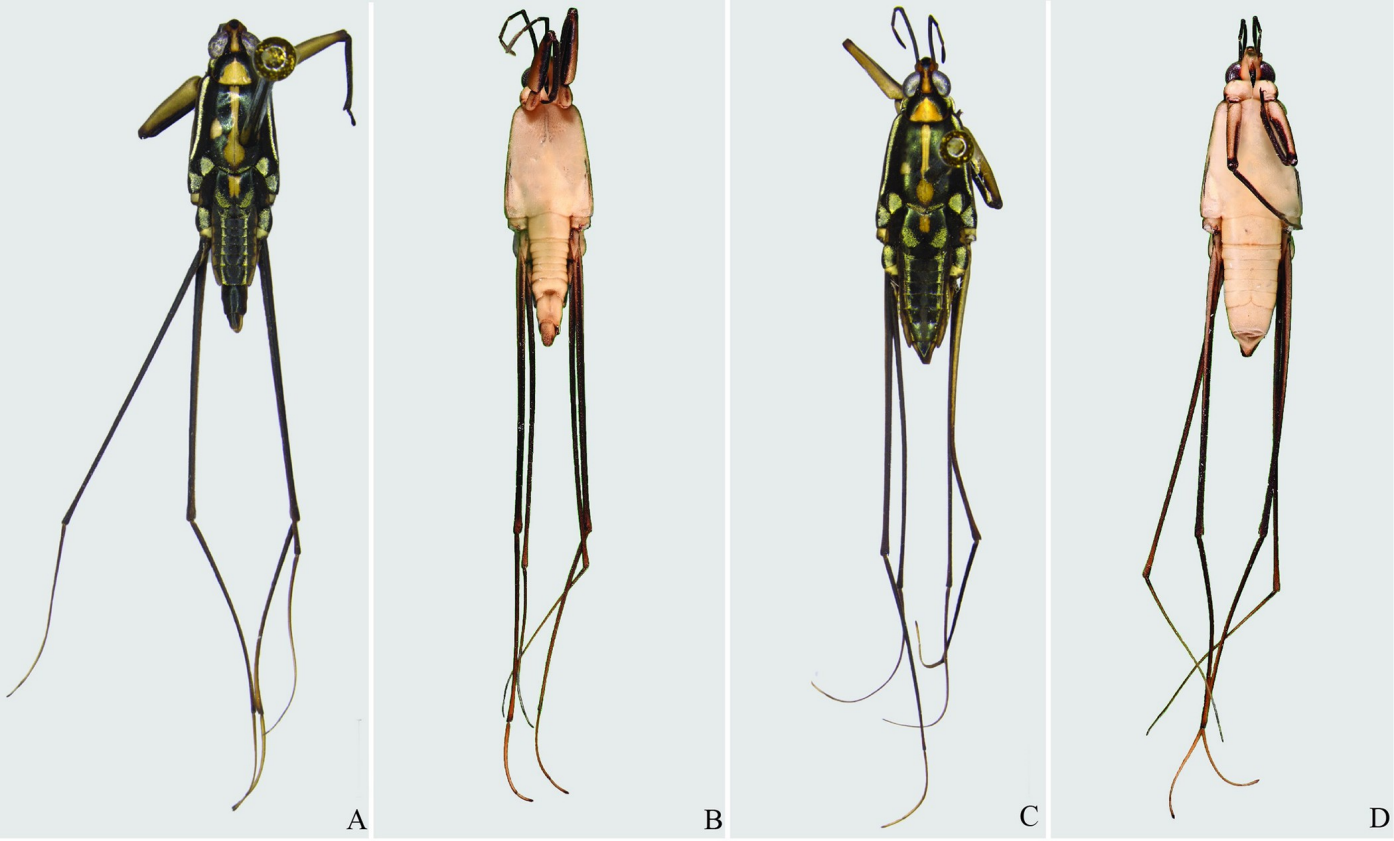
*Potamobates unidentatus*. (A) Male, dorsal view; (B) male, ventral view; (C) female, dorsal view; (D) female, ventral view.

**2**’–Pronotum with a wedge-shaped mark (Figs 23A, 23C, 24A and 24C) … 5

**3**– Posterior margin of male abdominal sternum VIII with 2 projections (Fig 2A and 2B)… *P*. *bidentatus*

**3**’–Posterior margin of male abdominal sternum VIII with at most 1 projection (Figs 8E and 16B)… 4

**4**– Male abdominal tergum VIII about 3 times longer than wide, curved ventrally; male abdominal sternum VIII with a median sulcus throughout length (Fig 8B, 8E and 8F); female abdominal tergum VIII about 3 times longer than wide, with acute apex (Fig 8C and 8D)… *P*. *osborni*

**4**’–Male abdominal tergum VIII 2–3 times longer than wide, not curved ventrally; male abdominal sternum VIII with a median sulcus only on anterior third (Fig 7A, 7B, 7E and 7F); female abdominal tergum VIII subequal in length and width, with rounded apex (Fig 7C) … *P*. *horvathi*

**Fig 25:**
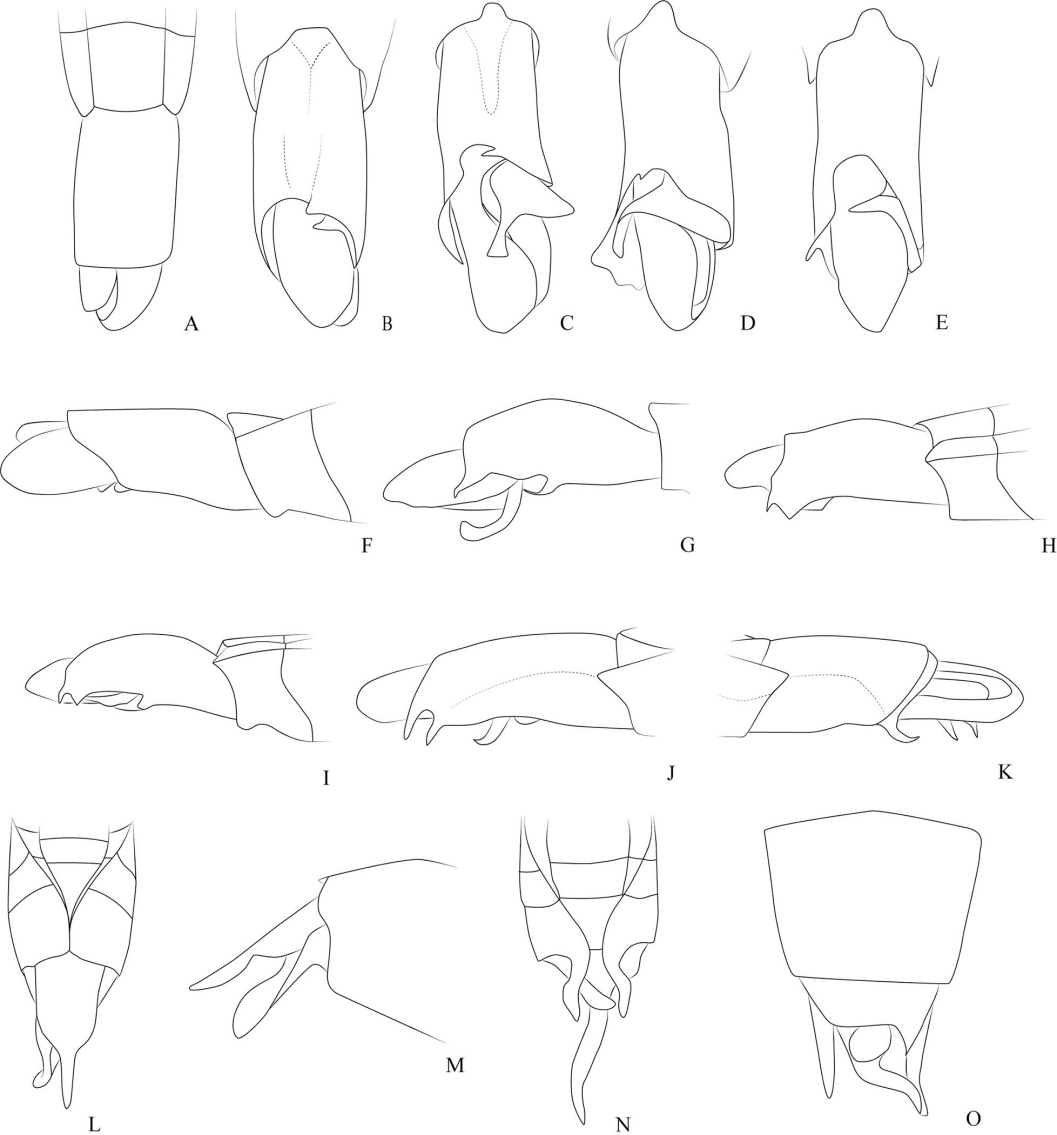
*P*. *bilobulatus* (B, G, O); *P*. *manzoneae* (A, C, H, M, Q); *P*. *shuar* (D, I); *P*. *spiculus* (E, J, K, N); *P*. *tridentatus* (F, L, P). (A) Male, terminalia, dorsal view; (B-F) Male, terminalia, ventral view; (G-L) Male, terminaria, lateral view; (M, N) Female, terminalia, dorsal view; (O, P) Female, terminalia, ventral view; (Q) Female, terminalia, lateral view.

**5**– Male pygophore and proctiger sinistrally rotated about 30° (Fig 25A and 25C); female abdominal tergum VIII about twice as long as mediotergite VII, with lateral margins abruptly converging on posterior 2/3 and not curved ventrally (Fig 25M); ratio between projection of female abdominal sternum VII and total length of sternum VII 0.7:1.0… *P*. *manzanoae*

**5**’–Male pygophore and proctiger sinistrally rotated about 20° (Fig 10A); female abdominal tergum VIII subequal in length to mediotergite VII, with lateral margins uniformly converging posteriorly and curved ventrally (Fig 10B); ratio between projection of female abdominal sternum VII and total length of sternum VII 0.5:1.0 (Fig 10C)… *P*. *unidentatus*

**6–** Left basolateral process of male proctiger bifid or with divergent lateral margins (Fig 11H)… 7

**6**’–Left basolateral process of male proctiger not bifid, with convergent lateral margins… 9

**7**– Male mesosternum with a posterolateral patch of black setae (Fig 14B); posterior lateroventral margin of male abdominal segment VIII without distinct projections, only weakly prolonged (Fig 3B, 3E and 3F); extension of female abdominal sternum VII almost completely covering tergum VIII (Fig 3C and 3D)… *P*. *carvalhoi*

**Fig 26:**
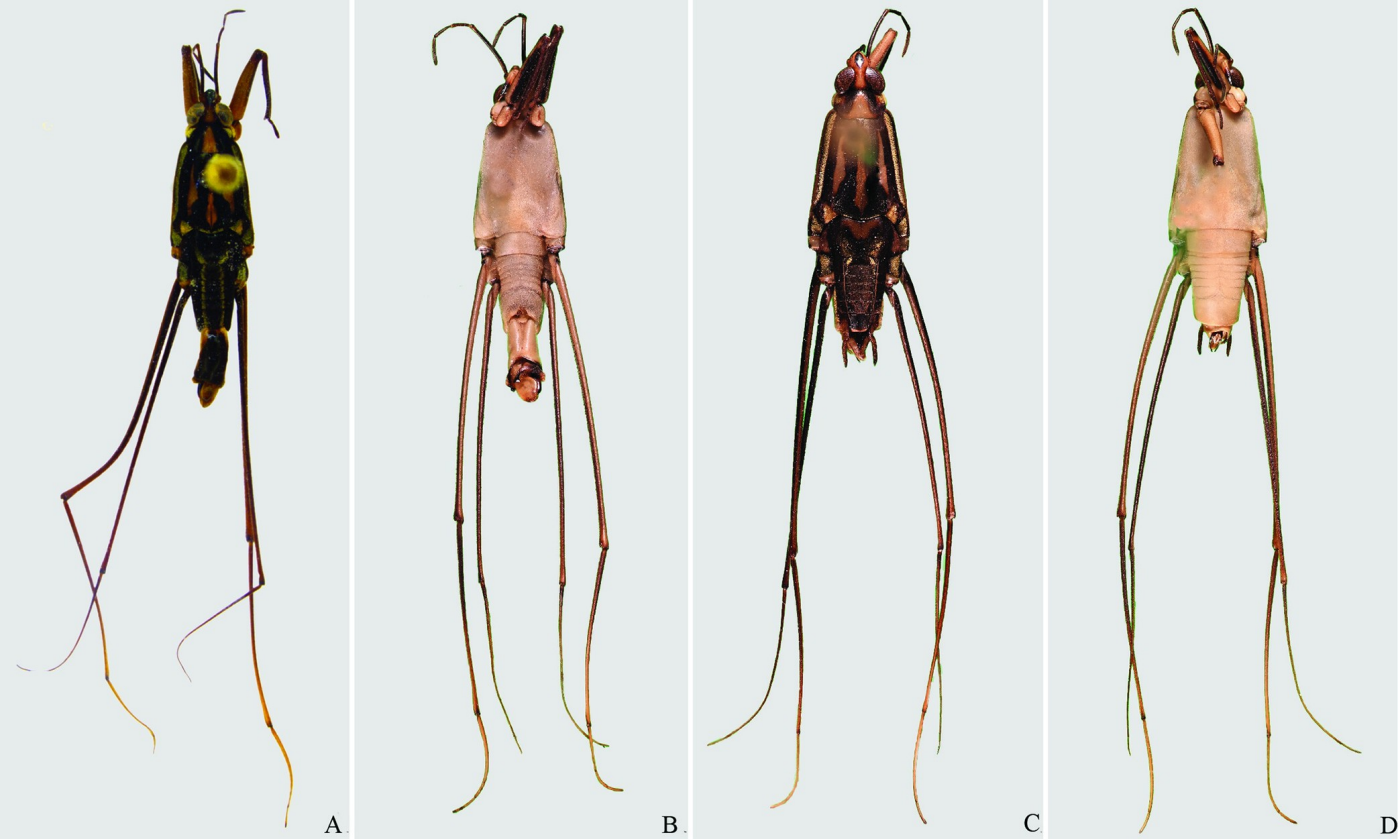
*Potamobates shuar*. (A) Male, dorsal view; (B) male, ventral view; (C) female, dorsal view; (D) female, ventral view.

**7**’–Male mesosternum without posterolateral patch of black setae (Fig 26B); posterior lateroventral margin of male abdominal segment VIII with one or two projections (Fig 11B–11D); extension of female abdominal sternum VII covering at most half of tergum VIII (as in Figs 1C, 1D, 12C and 18C) … 8

**8**– Left basolateral process of male proctiger with apex bifid (Fig 11H) … *P*. *variabilis*

**8**’–Left basolateral process of male proctiger with lateral margins divergent, but not bifid at the apex (Fig 25D and 25I) … *P*. *shuar*

**9**– Left basolateral process of male proctiger about as long as wide; apex of female abdominal tergum VIII ventrally curved (Fig 25I)… *P*. *bilobulatus*

**Fig 27:**
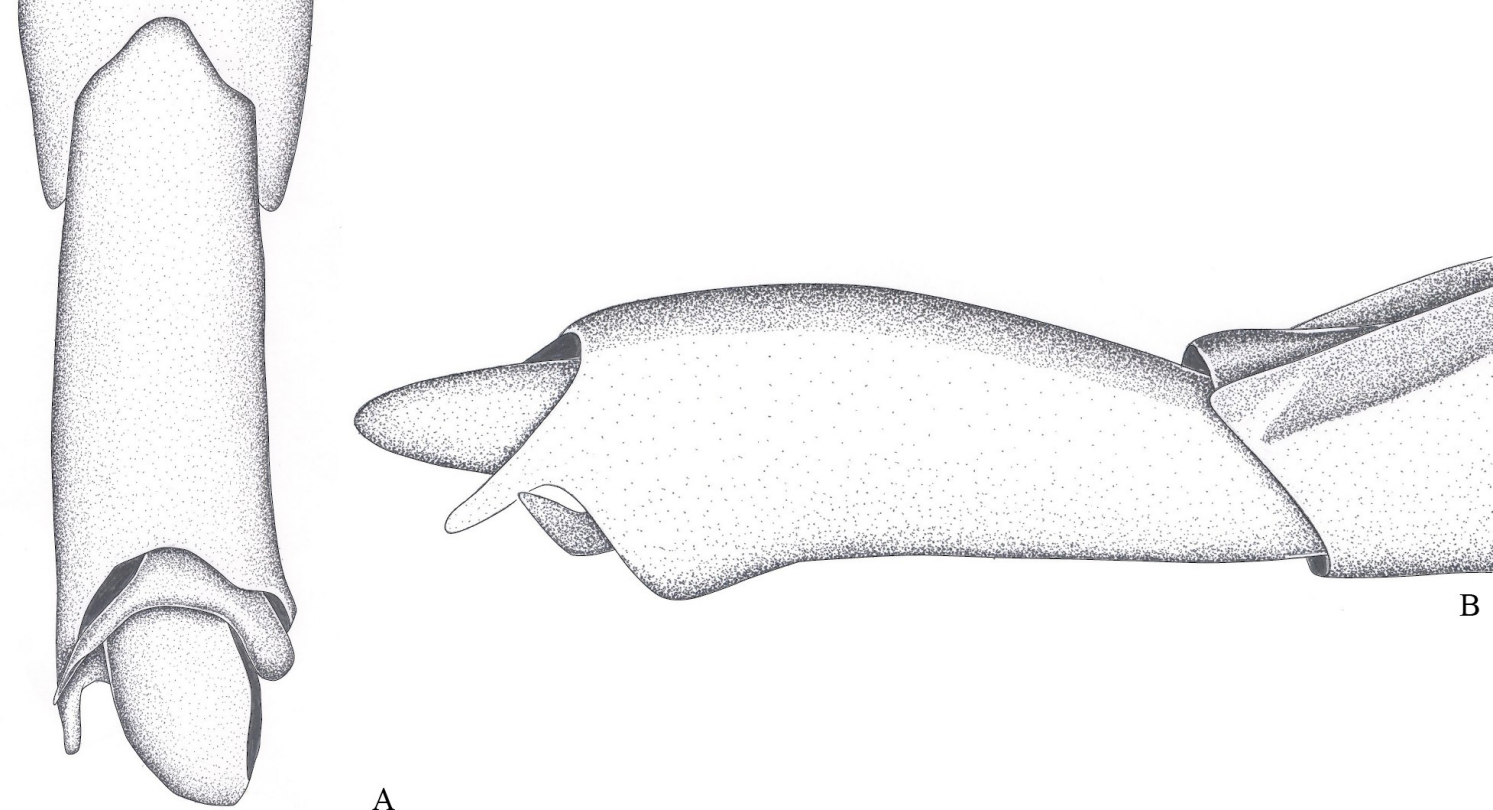
*Potamobates vivatus*. (A) Male, terminalia, ventral view; (B) male, terminalia, right lateral view.

**Fig 28:**
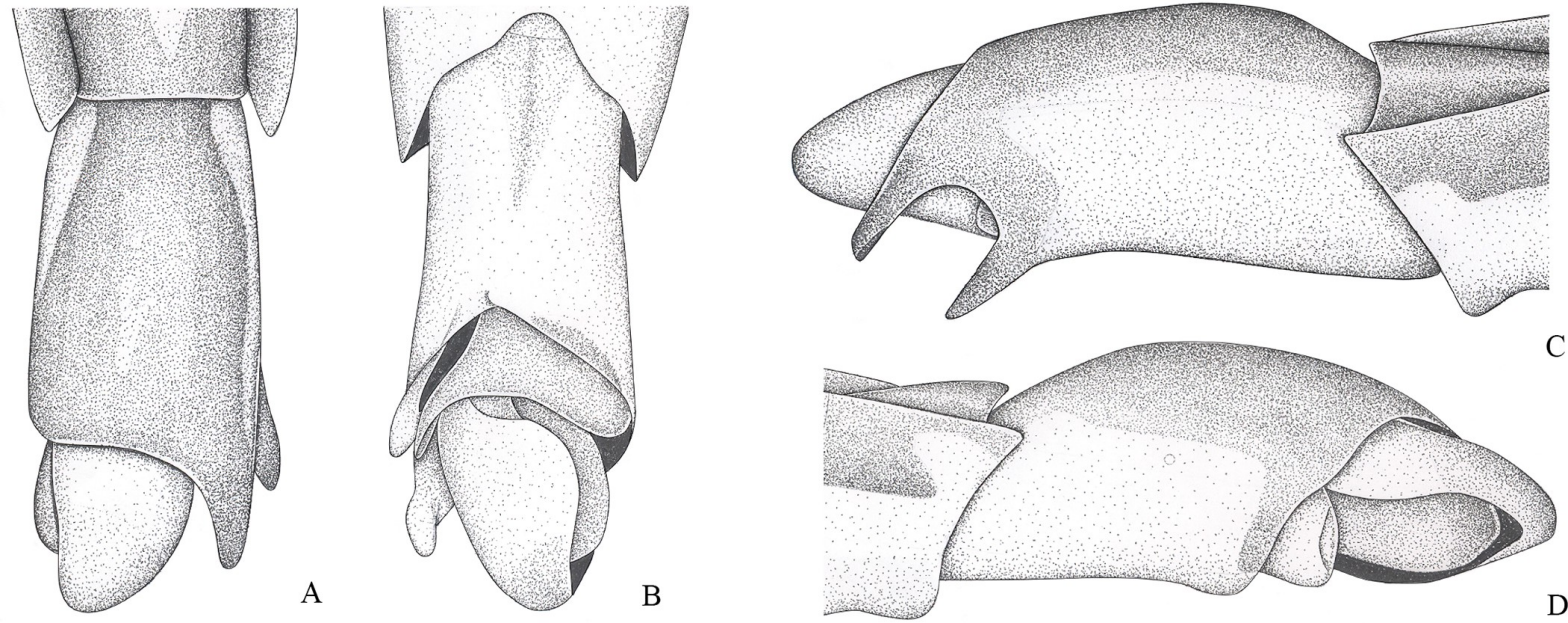
*Potamobates williamsi*. (A) Male, terminalia, dorsal view; (B) male, terminalia, ventral view; (C) male, terminalia, right lateral view; (D) male, terminalia, left lateral view.

**9**’–Left basolateral process of male proctiger at least twice as long as wide (Figs 1A, 9A, 18E, 19A, 27A and 28B); female abdominal tergum VIII variable … 10

**10**– Lobule of extension of female abdominal sternum VII subequal to or longer than median length of sternum (Figs 19B and 20D) … 11

**10**’–Lobule of extension of female abdominal sternum VII shorter than median length of sternum (Figs 1D, 13C, 29D, 30D, 31D, 32D, 33D and 34D) … 12

**11**– Female abdominal tergum VIII about 3 times longer than wide; (Figs 20C and 25N); posterior projection of last female abdominal laterotergite subequal to median length of mediotergite VII (Figs 20C and 25N)… *P*. *spiculus*

**11**’–Female abdominal tergum VIII subequal in length and width (Fig 35C); posterior projection of last female abdominal laterotergite about 3 times longer than mediotergite VII (Figs 19B and 35C) *… P*. *woytkowskii*

**12**– Right margin of male abdominal segment VIII with 1 projection (Figs 12D, 18E and 27B) … 13

**12’–**Right margin of male abdominal segment VIII with 2 projections (Figs 1G, 9B, 9C, 9D, 9E and 28C) … 15

**13**– Lateral margins of male proctiger folded ventrally; female abdominal tergum VIII subequal in length and width (Fig 12B) … *P*. *peruvianus*

**13’**–Lateral margins of male proctiger not folded ventrally; female abdominal tergum VIII twice as long as wide (Fig 18B and 18C) … 14

**14**– Right margin of male abdominal segment VIII with a long, narrow, 2.0–3.3 times as long as wide projection on the dorsal angle (Fig 27B) … *P*. *vivatus*

**14**’–Right margin of male abdominal segment VIII with a short, broad, about as long as wide projection on the ventral angle (Fig 18E) … *Potamobates molanoi* Floriano and Moreira, **sp. nov.**

**15**– Right posterolateral margin of male abdominal segment VIII with a dorsal and a ventral projection, the distance between them subequal to their length (Figs 9B–9E and 28C) … 16

**15’**–Right posterolateral margin of male abdominal segment VIII with two ventral projections connected by the base (Fig 1G) … 17

**16–** Left basolateral process of male proctiger abruptly twisted by 90° at the base (Fig 9A) … *P*. *sumaco*

**16**’–Left basolateral process of male proctiger twisted throughout its length (Fig 28B)… *P*. *williamsi*

**17**– Posterior margin of abdominal sternum VIII with a short notch; left basolateral process of male proctiger positioned above or beneath the projections of the right margin of segment VIII (Fig 1B) … *P*. *anchicaya*

**17**’–Posterior margin of abdominal sternum VIII with a distinct, laterally directed, notch (Fig 25F); male proctiger dislocated to the left, the left basolateral process not reaching the projections of the right margin of segment VIII (Fig 25F) … *P*. *tridentatus*

**Fig 29:**
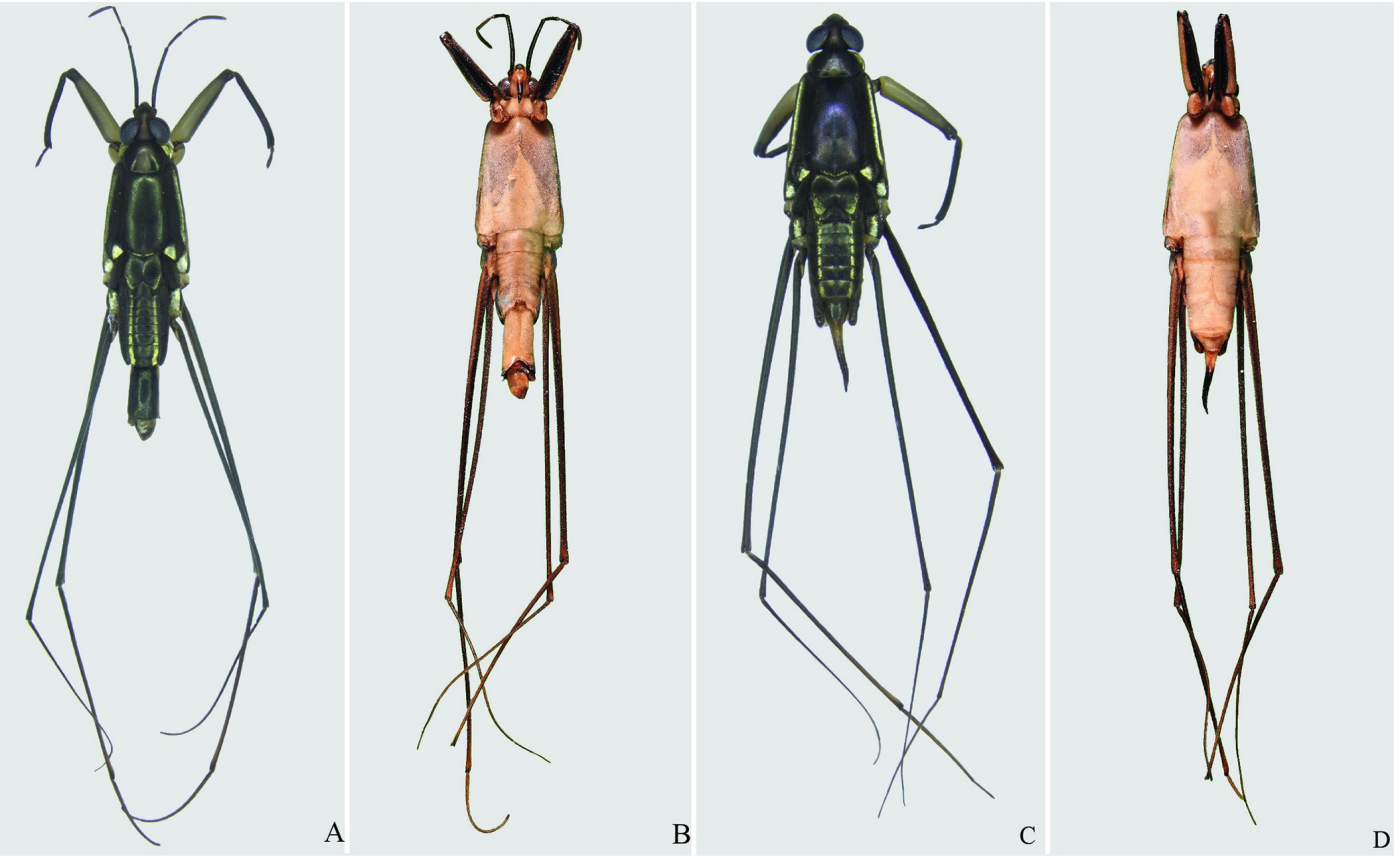
*Potamobates molanoi* sp new. (A) Male, dorsal view; (B) male, ventral view; (C) female, dorsal view; (D) female, ventral view.

**Fig 30:**
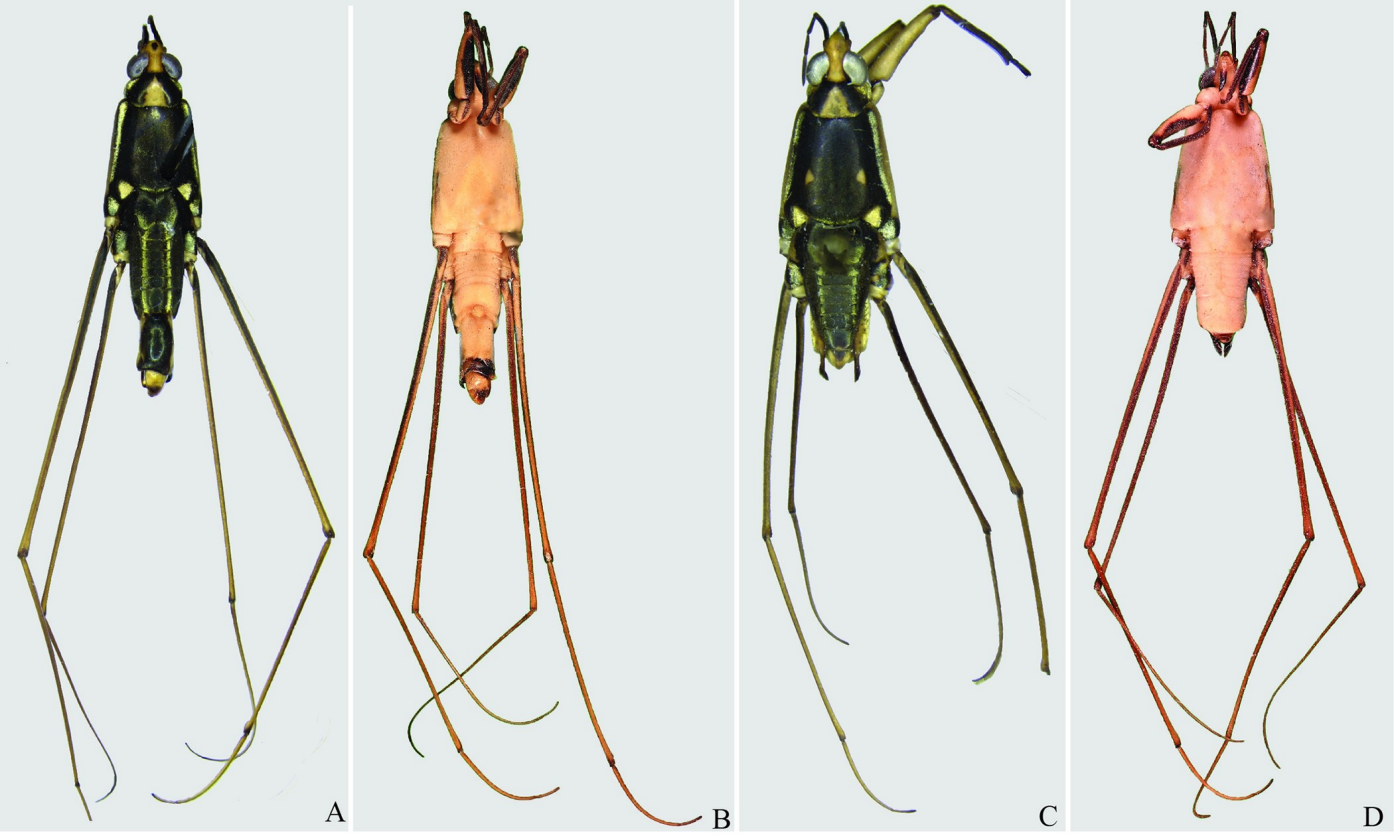
*Potamobates peruvianus*. (A) Male, dorsal view; (B) male, ventral view; (C) female, dorsal view; (D) female, ventral view.

**Fig 31:**
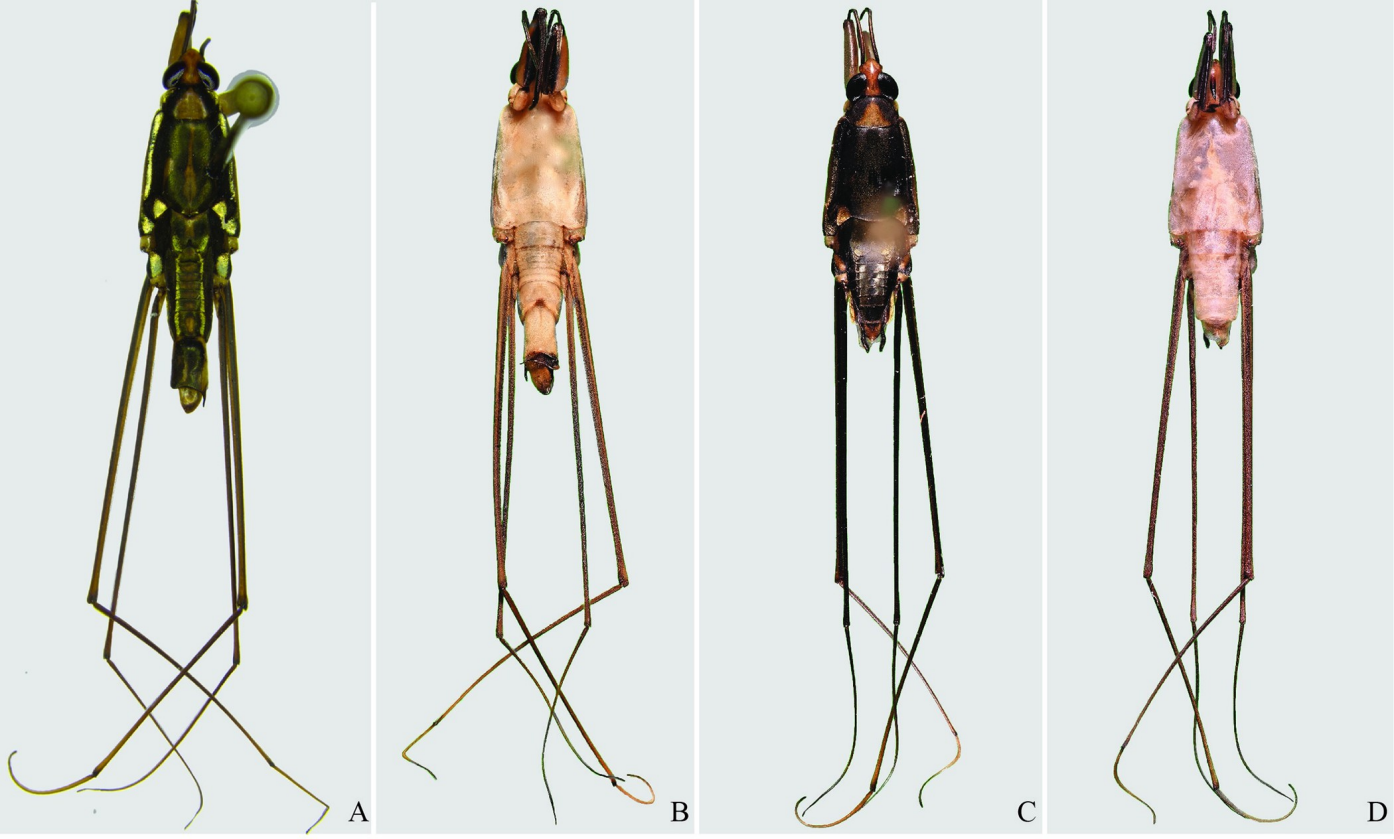
*Potamobates sumaco*. (A) Male, dorsal view; (B) male, ventral view; (C) female, dorsal view; (D) female, ventral view.

**Fig 32:**
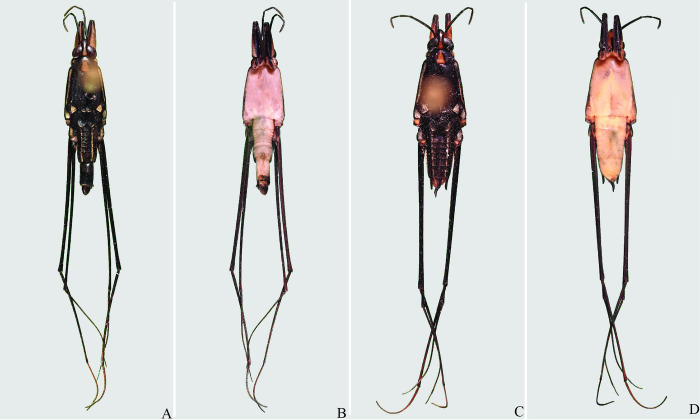
*Potamobates tridentatus*. (A) Male, dorsal view; (B) male, ventral view; (C) female, dorsal view; (D) female, ventral view.

**Fig 33:**
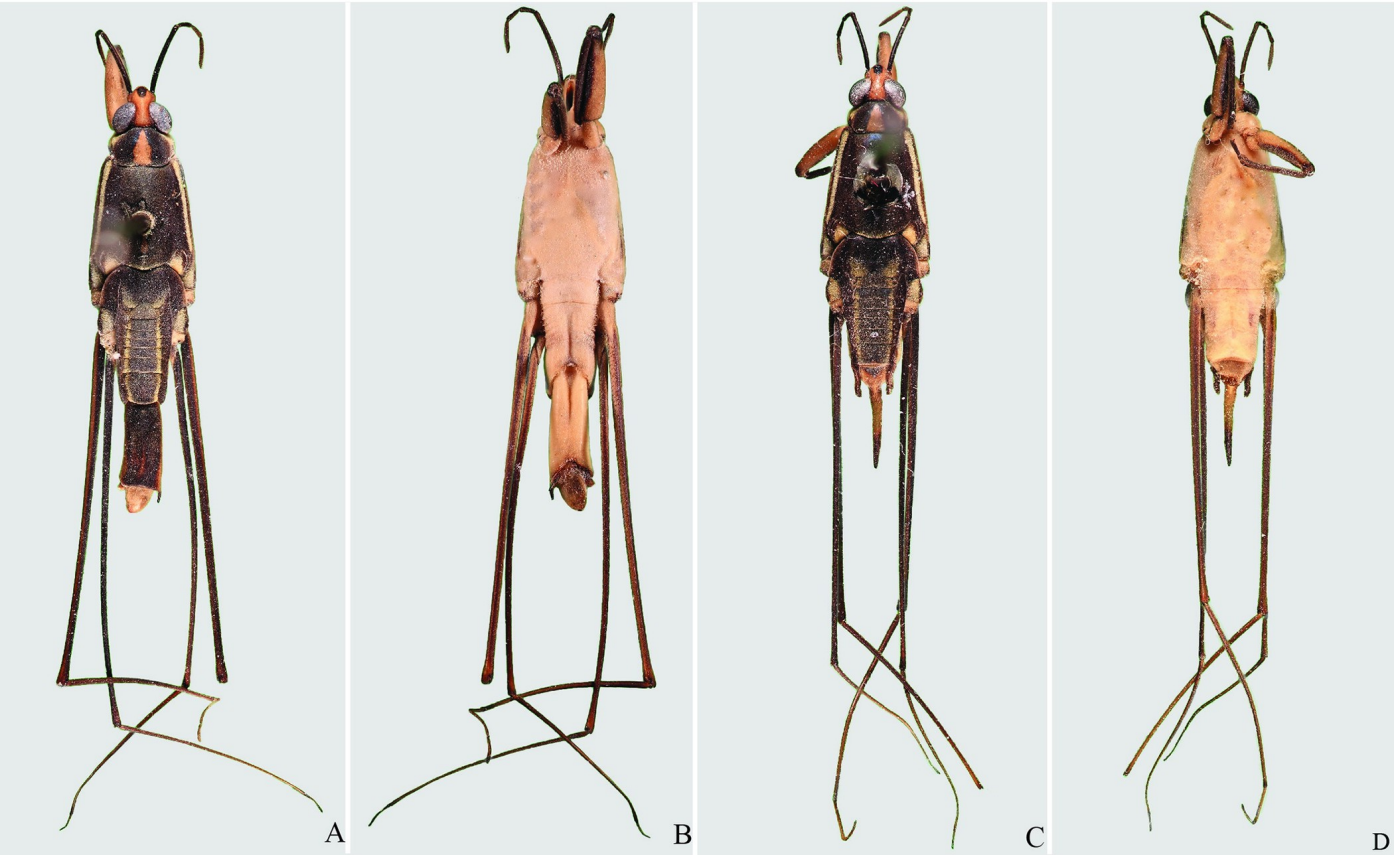
*Potamobates vivatus*. (A) Male, dorsal view; (B) male, ventral view; (C) female, dorsal view; (D) female, ventral view.

**Fig 34:**
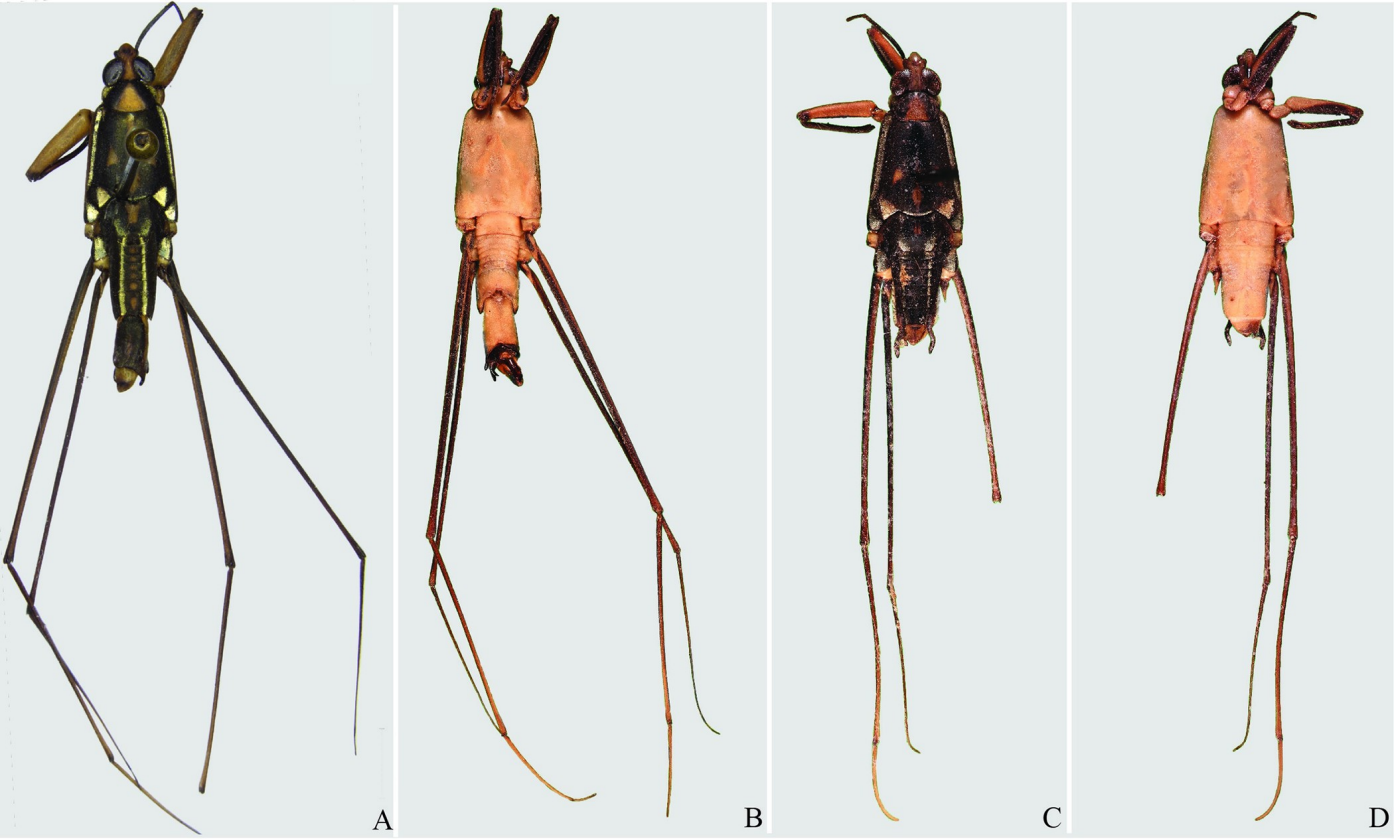
*Potamobates williamsi*. (A) Male, dorsal view; (B) male, ventral view; (C) female, dorsal view; (D) female, ventral view.

**Fig 35:**
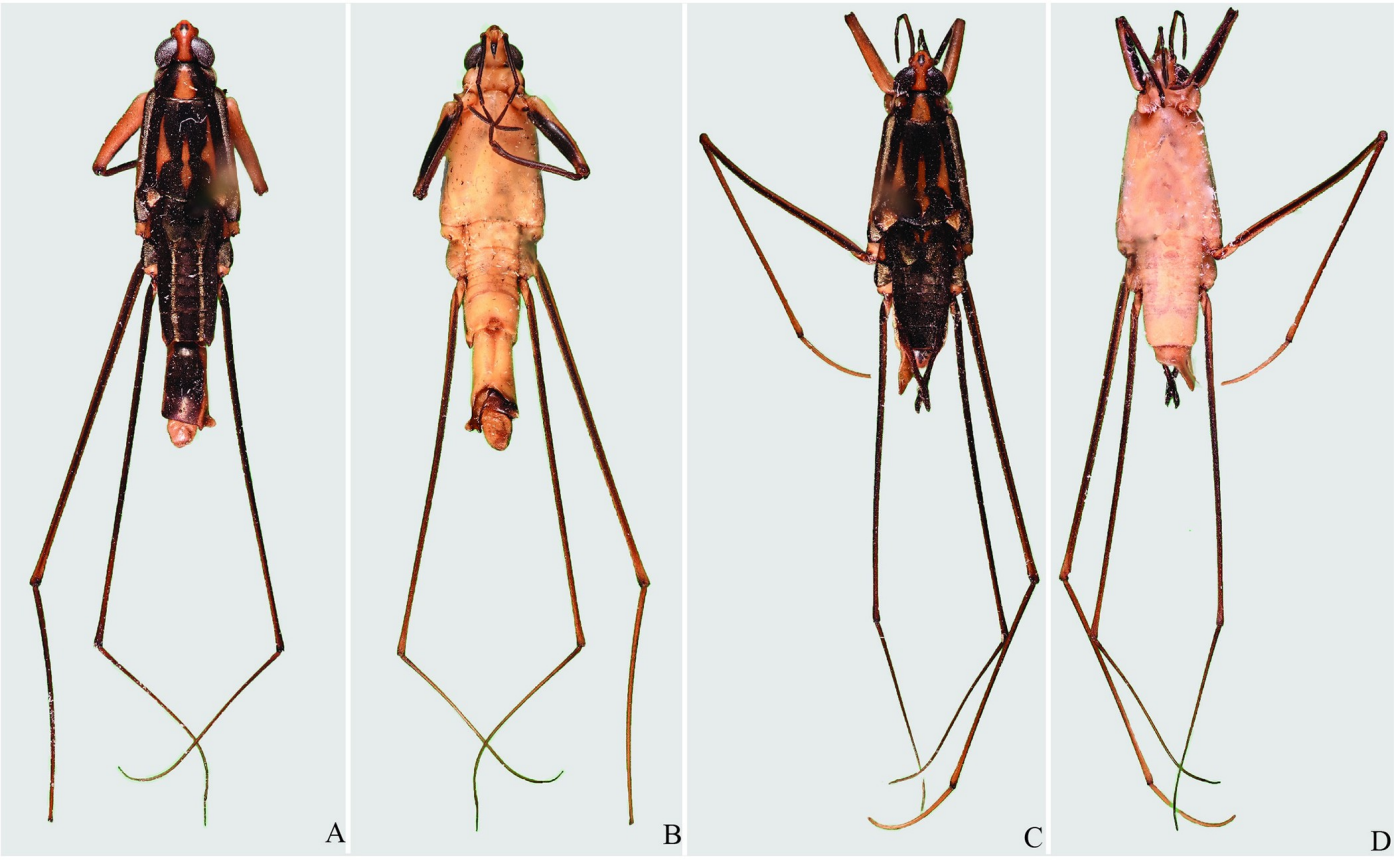
*Potamobates woytkowskii*. (A) Male, dorsal view; (B) male, ventral view; (C) female, dorsal view; (D) female, ventral view.

### *Potamobates anchicaya* Polhemus and Polhemus, 1995

(Figs 1 and 13)

*Potamobates anchicaya* Polhemus and Polhemus, 1995 [6]: 353–356, 361, 365, 367–369, 372 (description, figures, key, phylogeny). Cognato (1998) [8]: 21, 22 (phylogeny). Buzzetti (2006) [19]: 52, 53, 55 (figure, note, phylogeny). Padilla-Gil and Damgaard (2011) [7]: 45, 46 (key, phylogeny). Morales et al. (2013) [5]: 194 (figure).

*Potamobates tridentatus*; Manzano et al. (1995) [29]: 53 (records). Molano et al. (2008) [30]: 45, 55 (habitat, record). Posso and González (2008) [31]: 232 (records); *non* Esaki, 1926 [10] (misidentifications).

#### Diagnosis

Male pygophore and proctiger sinistrally rotated about 90° (Fig 1A, 1B, 1E and 1G); right margin of male abdominal segment VIII with a pair of projections connected by the base (Fig 1G); female abdominal tergum VIII subequal in length to mediotergite VII (Fig 1C); extension of female abdominal sternum VII subequal in length to mediotergite VII and partially folded over tergum VIII (Fig 1D).

#### Comments

Males of *Potamobates anchicaya* have the pygophore and proctiger sinistrally rotated about 90° (Fig 1A, 1B, 1E and 1G), similarly to *P*. *bilobulatus*, *P*. *carvalhoi*, *P*. *molanoi* Floriano and Moreira, **sp. nov.**, *P*. *peruvianus*, *P*. *shuar*, *P*. *spiculus*, *P*. *sumaco*, *P*. *tridentatus*, *P*. *variabilis*, *P*. *vivatus*, *P*. *williamsi*, and *P*. *woytkowskii*. They can be distinguished from most of these species by the pair of projections on the right margin of abdominal segment VIII (Fig 1G), which is also found in *P*. *sumaco*, *P*. *tridentatus*, and *P*. *williamsi*. *Potamobates anchicaya* differs from *P*. *sumaco* and *P*. *williamsi* because the projections are connected by the base, while they are not in the other two species (Fig 9B). It can be separated from *P*. *tridentatus* based on the following features: notch on the posterior margin of male abdominal sternum VIII short (Fig 1B), while it is much more distinct and laterally directed in *P*. *tridentatus*; and left basolateral process of male proctiger positioned above or beneath the projections of the left margin of segment VIII (Fig 1B), whereas the proctiger is dislocated to the left and the left basolateral process does not reach the projections of the left margin of segment VIII in *P*. *tridentatus*. This last characteristic must be used with caution, because the proctiger is mobile, which can affect the positioning of the left basolateral process in relation to the projections of segment VIII.

**Fig 36:**
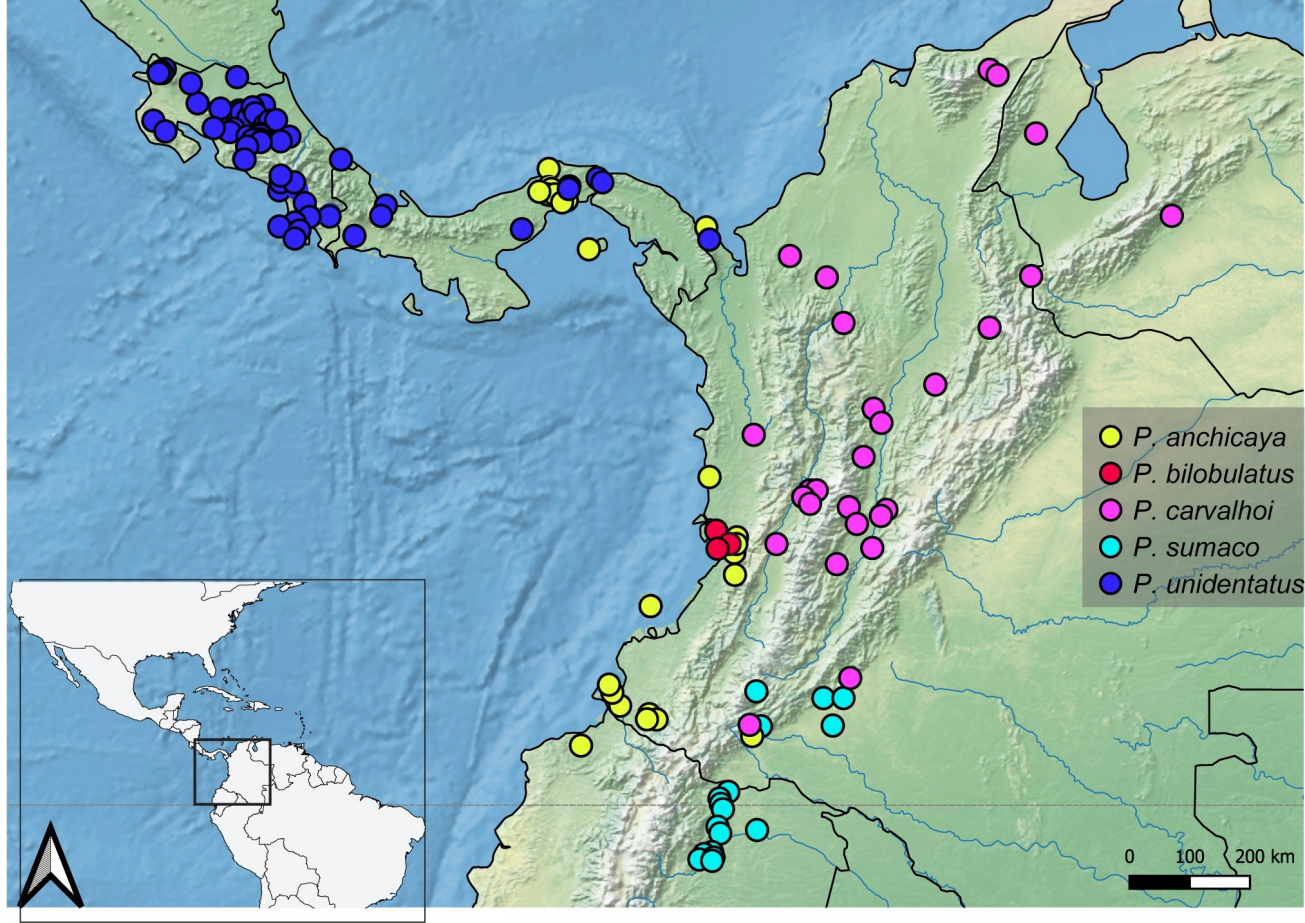
Map showing the geographical distribution of species of *P*. *anchicaya*, *P*. *bilobulatus*, *P*. *carvalhoi*, *P*. *sumaco*, *P. unidentatus*. Spatial data from Natural Earth (http://www.naturalearthdata.com/).

**Geographic distribution (Fig 36).** COLOMBIA: **Cauca** (Gorgona Island) [6, 17, 32 this work], **Chocó** [6, 32 this work], **Nariño** [33–37 this work], **Valle del Cauca** [6, 29–31, 38 this work]. ECUADOR: **Esmeraldas** [19]. PANAMA: **Colón** [6, 39 this work], **Kuna Yala** [6], **Panamá** [6]; [this work]. Records from East of the Andes (Mocoa, Putumayo, Colombia; [40, 41]) need verification.

#### Type material examined

4♂, 7♀ paratypes (USNM): ‘Colombia, Valle de Cauca\ swift rocky tributary\ to Rio Anchicaya, W. of\ Sabaletas, 100 m., 24°C.\ 30 July 1989 CL 2435\ D. A. & J. T. Polhemus’ ‘Paratype\ *Potamobates*\ *anchicaya*\ J. & D. Polhemus’ ‘J. T. Polhemus\ Collection 2014\ C. J. Drake Accession’. 4♂, 5♀ paratypes (USNM): ‘Panama, Canal Zone\ stream in culvert at Km.\ 10.6 on Pipeline Road\ 6 January 1993 CL 2790\ J. Polhemus & A. Gillogly’ ‘Paratype\ *Potamobates*\ *anchicaya*\ J. & D. Polhemus’ ‘J. T. Polhemus\ Collection 2014\ C. J. Drake Accession’. 1♂, 1♀ paratypes (USNM): ‘Panama\ Pearl Is\ San Jose’ ‘Morrison\ JPE July\ 30, 1944’ ‘Paratype\ *Potamobates*\ *anchicaya*\ J. & D. Polhemus’ ‘J. T. Polhemus\ Collection 2014\ C. J. Drake Accession’. 1♂ paratype (USNM): ‘Canal Zone\ Pan. 2-10-39\ C.J. Drake’ ‘Paratype\ *Potamobates*\ *anchicaya* J. & D. Polhemus’ ‘J. T. Polhemus\ Collection 2014\ C. J. Drake Accession’.

#### Additional material examined

1♀, 1♂ (USNM): ‘Barro Colorado\ C. Z. Panama\ Feb. 6–8, 1939\ Carl J. Drake’ ‘C J Drake\ Coll. 1956’. 1♀, 1♂ (USNM): ‘Panama\ Pearl Is\ San Jose’ Morrison\ JPE July\ 30, 1944’. 1♀ (USNM): ‘Juan Diaz\ Panama\ II-2-1935’ ‘C J Drake\ Coll. 1956’. 1♀ (USNM): ‘Panama\ Pearl Is\ San Jose’ ‘Morrison\ JPE July\ 30, 1944’. 1♀, 2♂ (USNM): ‘Barro Colorado\ C. Z. Panama\ Feb. 6–8 1939\ Carl J. Drake’ ‘C J Drake\ Coll. 1956’. 4♀ (USNM): ‘Canal Zone\ Pan. 2-10-39\ C. J. Drake’ ‘C J Drake\ Coll. 1956’. 1♀, 1♂ (UPTC): ‘Colombia Choco Acandí\ Capurgana 8°37’37.89’’N\ 77°20’53.16’’O 15m Qda. Jardín\ Botánico. Jama acuática\ 16.I.2008 López, Estupiñan y Molano’. 6♀, 3♂ (UPTC): ‘Colombia Choco Acandí Capurgana\ 8°37’37.89”N 77°20’53.16”O 15m\ Quebrada Jardín Botanico. Colecta\ Jama acuática. 16 I 2008. Lopez\ Estupiñan & Molano’ ‘Ordem Hemiptera\ Família Gerridae\ Género *Potamobates*\ Espécie: *Potamobates anchicaya*\ Determinó: Estupiñan A.’ ‘P. an. 0027’. 1♀ (UPTC): Colombia Cauca\ Lugar: Gorgona\ Camaronera\ Altura:\ Fecha: 26/11/88\ Col: H. González’ ‘Gen: *Potamobates*\ Esp: *anchicaya*\ Det. F. Molano’ ‘P. an. 0025’. 1♀, 1♂ (UPTC): ‘Colombia Choco Acandí Capurgana. 8°3737,89”N 77°2’53,16”O 15m\ Quebrada Jardim Botanico, colecion\ Jama acuatica. 16-I-2008. ‘Molano\ Lopez & Estupiñan.’ Ordem Hemiptera\ Familia Gerridae\ Genero *Potamobates*\ Espécie *Potamobates anchicaya*\ Determinó Estupiñan A.’ ‘P. an. 0023’. 3♀, 1♂ (UPTC): ‘Colombia–Vale B.ventu\ Lugar: San Cipriano\ Altura: 100 msnm\ Fecha: 15/05/05\ Col. F. Molano’ ‘Gen: *Potamobates*\ Esp: *anchicaya*\ Det: F. Molano’ ‘P. an. 0024. 4♀ (MUSENUV): ‘Colombia–Valle\ Buenventura\ Quebrada después\ de R. água clara\ via a B. ventura\ M.R. Manzano\ Julio 30 de 1989’ ‘MRN Agua Clara 018’ ‘F2409’ ‘Gerridae\ *Potamobates*\ *tridentatus*\ F. molano’. 6♀, 3♂, 2 nymphs (MUSENUV): ‘Colombia Valle\ Rio tatabro\ Feb 29–87\ R. González’ ‘Gerridae\ *Potamobates*\ *tridentatus*\ F. Molano’ ‘F 2408’ ‘EIS 87004’. 1♀, 1 nymph (MUSENUV): ‘Colombia–Valle\ Rio Tatabro\ 150 msnm\ Feb 280 87\ R. González’ ‘Gerridae\ *Potamobates*\ *tridentatus*\ R. González E-15-87005’ ‘F2407’. 1♀, 1♂ (MUSENUV): ‘Colombia Valle\ Quebrada Narcisa\ 95 msnm\ Charco superfície\ Col: R. González’ ‘Gerridae\ *Potamobates*\ *tridentatus*\ N. Nieser’ ‘E17-J3-87016’ ‘F2404’. 4♀, 3♂, 9 nymphs (MUSENUV): ‘Colombia\ Rio Tatabro\ Julio 30/89\ M.R. Manzano’ ‘Gerridae\ *Potamobates*\ *tridentatus*\ F. Molano’ ‘F2413’. 2♀, 1♂, 3 nymphs (MUSENUV): ‘Colombia–Valle\ Rio tatabro\ Feb 28/87\ R. González’ ‘Gerridae\ *Potamobates*\ *tridentatus*\ F. Molano’ ‘F2412’. 1♂, 2♀ (MUSENUV): ‘Colombia–Valle\ Rio tatabro\ 150 msnm\ Feb 28/87\ R. González’ ‘Gerridae\ *Potamobates*\ *tridentatus*\ F. Molano’ ‘E15-51-87001’ ‘F2420’. 1♀, 1♂ (MUSENUV): ‘Colombia–Cauca\ Isla de Gorgona\ Acueducto\ Sept 9 1987\ Col: C. Murillo’ ‘Gerridae\ *Potamobates*\ *tridentatus*’ ‘840185CAM’ ‘81–0003’ ‘FL402’. 1♀ (MUSENUV): Colombia–Valle\ Bajo calima\ Marzo 22/87\ 70 msnm\ N. Nieser’ ‘Gerridae*\ Potamobates*\ *tridentatus*\ Es.’ ‘F2515’ ‘E17-5187013’. 1♀ (MUSENUV): ‘Colombia–Valle\ Rio tatabro\ Feb. 28/87\ 150 msnm\ R. González’ ‘Gerridae\ *Potamobates*\ *tridentatus* ES.\ N. Nieser’ ‘F2403’ ‘E15-51-87001’. 1♀ (MUSENUV): ‘Colombia Valle\ Rio tatabro\ 150 msnm\ Riachuelo (± 155 m)\ Coll R. González’ ‘Gerridae\ *Potamobates*\ *tridentatus*\ N. Nieser’ ‘F2405’ ‘E15-87004’. 1♀, 2♂ (MUSENUV): ‘Colombia–Valle\ Bajo Calima\ Bosque sombra\ Marzo 3/87\ M.R. Manzano’ ‘Gerridae\ *Potamobates*\ *tridentatus*\ M.R. Manzano’ F2401’. 3♀, 1♂, 4 nymphs (MUSENUV): ‘Q. Bartolino\ (Superfície)\ abril/6/92\ F. Gerridae\ *Trepobates*’. 1♀, 1♂ (MUSENUV): ‘La Ecopera\ (Superfície)\ abril 13/92\ F. Gerridae\ *Trepobates*’. 1♀, 1♂ (MUSENUV): ‘Colombia. Cauca\ Isla Gorgona. Parque Natural Nacional\ Isla Gorgona. Quebrada Chorro del Cura N 02° 5821.5”; W 078°10’\ 43.4”. 90 metros. Febrero 26–2011. Col: M.del.Zuñiga, W. Cordona., R.\ J. Cordozo-Zuñiga. Microhábitat\ Superfície del água’ ‘*Potamobates* sp’.

### *Potamobates bidentatus* Champion, 1898

(Figs 2 and 15)

*Potamobates bidentatus* Champion, 1898 [8]: 154, 155, 453 (description, figure). Drake and Harris (1934) [21]: 224, 227, 228, 240, 241 (figure, key, redescription). Kuitert (1942) [17]: 140, 142 (key, note). Polhemus and Polhemus (1995) [6]: 364, 363, 366–368, 372 (figure, key, phylogeny, redescription). Cognato (1998) [18]: 21, 22 (phylogeny). Buzzetti (2006) [19]: 55 (phylogeny). Padilla-Gil and Damgaard (2011) [7]: 44, 46 (key, phylogeny).

#### Diagnosis

Male pygophore and proctiger sinistrally rotated about 20°; male abdominal segment VIII with a pair of projections on the left posteroventral margin, each projection with length subequal to width (Fig 2A); female abdominal tergum VIII about twice as long as mediotergite VII (Fig 15C); extension of female abdominal sternum VII about three times as wide as long, first pair of gonocoxae partially exposed, anal cone long (Fig 15D).

#### Redescription

Length (**♂**: 10.9–11.1; ♀: 11.1–11.4); width (**♂**: 3.1; ♀: 3.3–3.5) (Fig 15). *Head*: antennomere I about 1.3 times longer than head width, about twice as long as antennomere II; II about 1.5 times longer than III; IV subequal in length to III. Eye width 1.2–1.5 times interocular width. Article III of labium twice as long as IV. Antenna and dorsum of head black; vertex with yellowish mark; mandibular and maxillary plates yellowish; labium with articles I and II yellowish, III and IV black; venter of head yellowish. *Thorax*: Pronotum with posterior margin covered by golden setae and narrow yellowish median stripe, stripe with half of width of yellowish mark of head vertex; propleuron with anterior patch of golden setae; proacetabulum dorsally black, with stripe of golden setae laterally, ventrally yellowish; fore coxa and trochanter yellowish, trochanter with dorsum and distal margin darkened; fore femur yellow, venter and apex black; fore tibia and tarsus black. Mesonotum without longitudinal stripes, posterior margin with golden setae; dorsal 3/4 of spiracle covered by golden setae. Mesopleuron with longitudinal stripe of golden setae, stripe sigmoid, slightly curved, posteriorly discontinue; mesosternum with blackish mark anteriorly below proacetabula and a pair of blackish marks on posterior third; limit between meso- and metasterna darkened; middle and hind coxae yellowish; middle and hind trochanters blackish; middle and hind femora entirely blackish or with venter yellowish; apices of middle and hind tibia, and entire middle and hind tarsi brown. Metanotum without stripes, posterior margin with golden setae; metacetabulum with stripe of golden setae laterally. *Abdomen*: medio- and laterotergites I–VII black, lateral margins of mediotergites with longitudinal stripe of golden setae; side of abdomen black, dorsally with longitudinal stripe of golden setae. *Male*: sides of abdominal sterna II–IV conspicuously longitudinally depressed; sternum VII posteriorly with a shallow, weak depression; VIII conspicuously longitudinally depressed. Abdominal mediotergite VII with posterior margin rounded. Posterior projection of last abdominal laterotergite (= connexival spine) with apex rounded. Abdominal sternum VII with oval notch on posterior margin, twice as wide as long (Fig 2A). Abdominal segment VIII tubular, 1.6–1.7 times longer than wide; lateral margins slightly convergent anteriorly and posteriorly; posterodorsal margin rounded; venter without depression; posteroventral margin rounded, with conspicuous projection on the left, the projection with pair of small teeth (Fig 2A). Pygophore and proctiger sinistrally rotated about 20° (Fig 2A and 2B). Pygophore 1.9 times longer than abdominal segment VII. Proctiger 1.5 times longer than abdominal segment VII; anterior margin with short notch, which has about 1/5 of proctiger length; right margin folded ventrally; left margin folded on basolateral process and part of proctiger; apex with many black denticles; right basolateral process developed, triangular, about as long as wide; left basolateral process 5 times longer than wide, with about 2/5 of proctiger length, longitudinal axis about 45° divergent from longitudinal axis of proctiger, lateral margins slightly tapering, apex not bifid (Fig 2F). Phallus short, smallest width about 3.8 times length (Fig 2C); dorsal sclerite slightly longer and narrower than ventral sclerite, with basal margin notched (Fig 2D); lateral sclerite triangular; base of ventral sclerite not widened, notched centrally, apex almost twice as wide as center and notched centrally (Fig 2E); transverse sclerite smaller, not folded over ventral sclerite (Fig 2C). Abdominal tergum VIII and proctiger black; pygophore yellow to brown, ventrally blackish. *Female*: Abdominal laterotergites not folded over mediotergites; last segment without posterior projection (= connexival spine). Posterior margin of abdominal sternum VII produced posteriorly, extension oval, about three times as wide as long, partially covering first pair of gonocoxae, not folded over tergum VIII. Abdominal tergum VIII triangular, 1.6–1.8 times longer than wide, lateral margins strongly converging posteriorly, apex acute. Anal cone long, about as long as abdominal tergum VIII. Extension of abdominal sternum VII yellowish. Dorsum of abdominal tergum VIII and anal cone black, lateral margins of tergum VIII, venter of anal cone and first pair of gonocoxae brown. *Macropterous*: posterolateral margin of pronotum brownish.

#### Variation

Base of antenniferous tubercle might have a yellowish mark. Distal half of fore femur might be blackish.

#### Comments

The male of *P*. *bidentatus* has the pygophore and proctiger weakly rotated, never reaching 45° (Fig 2A) in relation to the longitudinal axis of the body. A similar condition is found in *P*. *horvathi*, *P*. *manzanoae*, *P*. *osborni*, and *P*. *unidentatus*. However, while male *P*. *bidentatus* have two projections on the posteroventral margin of abdominal segment VIII (Fig 2A), the other species have only one projection.

**Fig 37:**
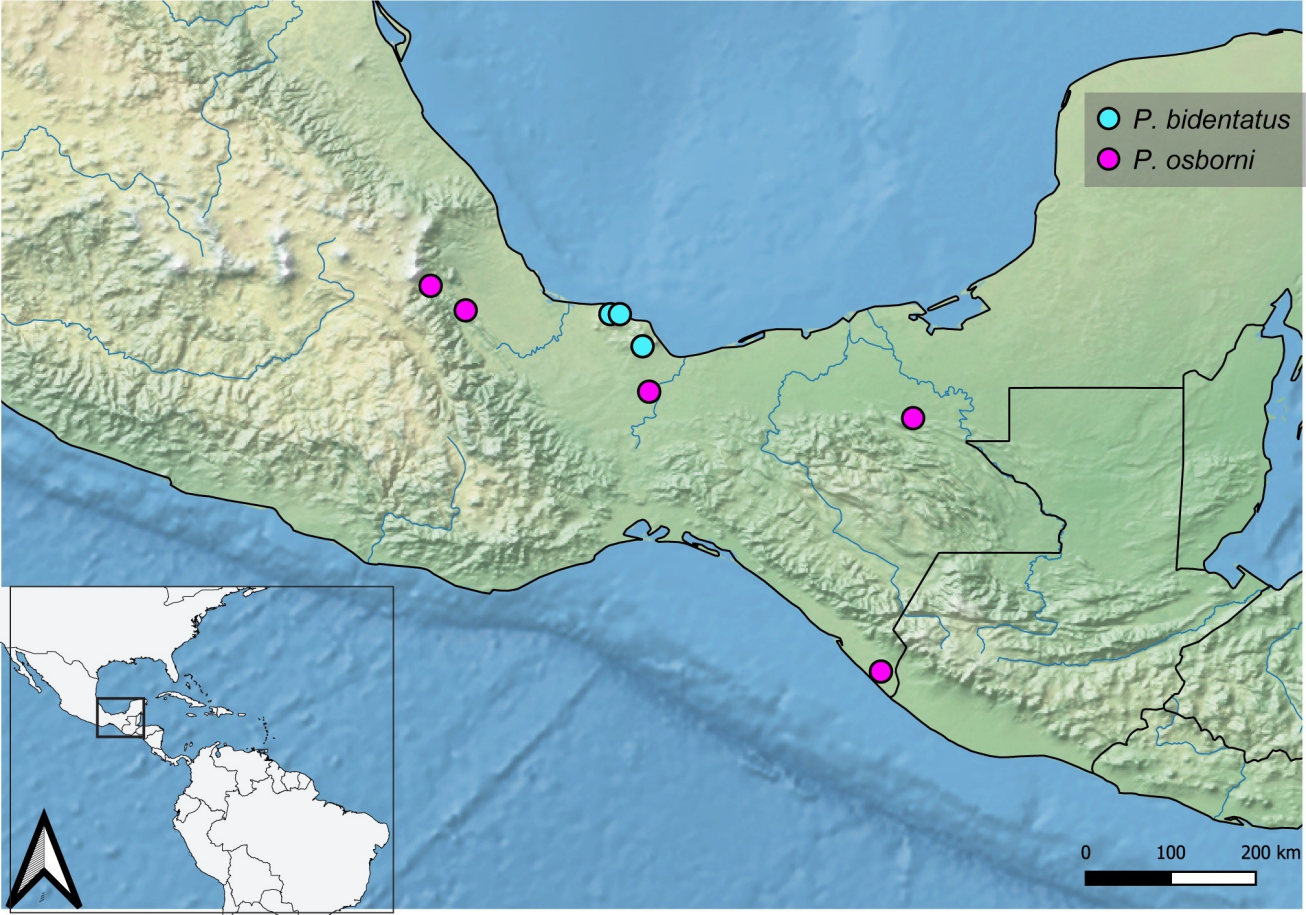
Map showing the geographical distribution of species of *P*. *bidentatus*, *P*. *osborni*. Spatial data from Natural Earth (http://www.naturalearthdata.com/).

**Geographic distribution (Fig 37).** MÉXICO [8]: **Veracruz** [6; this work]. This species is endemic from southeastern Veracruz State, Mexico [6; this work]. Records from South America [31, 42, 43] are based on misidentifications.

#### Material examined

1♀ (USNM): ‘Rio Maquinas\ Los tuxtlas\ Veracruz\ 11-V-81\ G. Ortega’ ‘J. T. Polhemus\ Collection 2014\ C.J. Drake Accession’. 1♀, 1♂ (USNM): ‘Rio Maquinas\ Los tuxtlas\ Veracruz\ 11-V-81\ P. Hernandez’ ‘O. Hemiptera\ F. Gerridae’ ‘J. T. Polhemus\ Collection 2014\ C.J. Drake Accession’. 1♀, 1♂ (USNM): ‘México\ Veracruz\ Los tuxtlas\ Rio Maquinas\ 5-V-81’ ‘Colección del Instituto\ de Biologia, UNAM.\ México, D.F.’ ‘*Potamobates*\ *bidentatus*\ Champion\ Det J. Polhemus’ ‘J. T. Polhemus\ Collection 2014\ C.J. Drake Accession’. 1♀, 2♂ (USNM): ‘Rio Maquinas\ Balzapote\ Veracruz\ 11-V-81\ G. Ortega’ ‘J. T. Polhemus\ Collection 2014\ C.J. Drake Accession’. 1♂ (USNM): ‘Rio Máquinas\ Los Tuxtlas\ Veracruz; único\ 12/V/83\ G. Ortega’ ‘Familia Gerridae\ *Limnogonus*\ Det. G. Ortega’ ‘J. T. Polhemus\ Collection 2014\ C.J. Drake Accession’. 1♀ (USNM): ‘Rio Máquinas\ Los Tuxtlas\ Veracruz; único\ 14/V/83\ G. Ortega’ ‘Familia Gerridae\ *Limnogonus*\ Stal \ Det. G. Ortega’ ‘J. T. Polhemus\ Collection 2014\ C.J. Drake Accession’. 1♀, 1♂ (USNM): ‘México\ Veracruz\ El ocotal chico\ 18-II-84\ H. Delfin’ ‘Colección Del Instituto\ de Biologia, UNAM\ México, D.F.’ ‘J. T. Polhemus\ Collection 2014\ C.J. Drake Accession’. 1♂ (USNM): ‘Rio Máquinas\ “Los tuxtlas” Ver.\ 5-V-81\ R. Novelo’ ‘Hemiptera\ Gerridae\ Det. R. Novelo’ ‘J. T. Polhemus\ Collection 2014\ C.J. Drake Accession’. 1♀ (USNM): ‘Rio Maquinas\ Los tuxtlas, ver\ 5-5-81\ M. Galicia’ ‘Hemiptera\ Gerridae\ M. Galicia det.’ ‘J. T. Polhemus\ Collection 2014\ C.J. Drake Accession’.

### *Potamobates bilobulatus* Morales, Molano and Castro, 2013

**(Fig 25B, 25G and 25O)**

*Potamobates bilobulatus* Morales, Molano and Castro, 2013 [5]: 189–195 (description, figure).

#### Diagnosis

Male pygophore and proctiger sinistrally rotated about 90° (Fig 25B and 25G); right posterolateral margin of male abdominal segment VIII with a bilobed projection, dorsalmost lobe larger, wide (length subequal to width), ventralmost lobe smaller (Fig 25G); left basolateral process of male proctiger about as long as wide, about half as long as proctiger, with apex rounded (Fig 25B); female abdominal tergum VIII 1.5 times longer than mediotergite VII, with apex curved ventrally; extension of female abdominal sternum VII long, not folded over tergum VIII, ratio between length of extension and total length of sternum VII 0.3:1.0 (Fig 25O).

#### Comments

*Potamobates bilobulatus* has the male pygophore and proctiger sinistrally rotated about 90°, similarly to *P*. *anchicaya*, *P*. *carvalhoi*, *P*. *molanoi* Floriano and Moreira, **sp. nov.**, *P*. *peruvianus*, *P*. *shuar*, *P*. *spiculus*, *P*. *sumaco*, *P*. *tridentatus*, *P*. *variabilis*, *P*. *vivatus*, *P*. *williamsi*, and *P*. *woytkowskii*. However, it can be distinguished from these species by the left basolateral process of the male proctiger about as long as wide and with about half of proctiger length, and the female with abdominal tergum VIII triangular, 1.5 times longer than mediotergite VII.

**Geographic distribution (Fig 36).** COLOMBIA: **Valle del Cauca** [5, this work].

#### Type material examined

1**♀** holotype (UPTC): ‘Colombia Valle del Cauca\ Buenaventura Bahía de Buenaventura La Bocana\ Qda. Aguaclara 13.VI.2005\ Col. F. Molano leg.’ ‘UPTC-MHN-ART 0006’ ‘*Potamobates*\ *bilobulatus*’ ‘Holotipo’. 1♂, 2♀ paratypes (UPTC): ‘Colombia Valle del Cauca\ Buenaventura, Bahia de\ Buenaventura La Bocana\ Qda. Aguaclara 13.vi.2005\ Col. F. Molano leg.’ ‘*Potamobates*\ *bilobulatus*’ ‘UPTC-MHN-ART 0007’ ‘Paratipos’.

#### Additional material examined

4♂, 3**♀** (EQ): ‘#442\ Hemiptera\ Gerridae\ *Potamobates bilobulatus\* Caño NN Est. San Antonio\ Buenaventura 8/08/01/ 15 individuos’. 7♂, 6**♀**, 20 nymphs (MUSENUV): ‘[Bahia de] Malaga\ Rio Bonquito\ (Superfície)\ 12 msnm\ Mayo 15/92\ F. Gerridae\ *Metrobates* sp.’.

### *Potamobates carvalhoi* Polhemus and Polhemus, 1995

(Figs 3 and 14)

*Potamobates carvalhoi* Polhemus and Polhemus, 1995 [6]: 351–353, 359, 364, 366–368, 372 (description, figure, key, phylogeny). Cognato (1998) [18]: 21, 22 (phylogeny). Buzzetti (2006) [19]: 55 (phylogeny). Padilla-Gil and Damgaard (2011) [7]: 45, 46 (key, phylogeny). Morales et al. (2013) [44]: 194 (figure).

*Potamobates peruvianus*; Aristizábal-García (2002) [38]: 92, 104–106 (key, figure, records); *non* Hungerford, 1936 [4] (misidentification).

#### Diagnosis

Posterolateral region of mesosternum with patch of black setae (Fig 14B); male pygophore and proctiger sinistrally rotated about 90° (Fig 3A, 3B, 3E and 3F); right posterolateral margin of male abdominal segment VIII expanded (Fig 3B, 3E and 3F); left basolateral process of male proctiger with apex bifid (Fig 3E); female abdominal tergum VIII subequal in length to mediotergite VII (Fig 3C); extension of female abdominal sternum VII subequal in length to mediotergite VII, totally folded over tergum VIII (Fig 3C and 3D).

#### Comments

This species can be separated from all congeners by the patch of black setae on the posterolateral region of the mesosternum (Fig 14B). The male of *P*. *carvalhoi* has the pygophore and proctiger sinistrally rotated about 90° (Fig 3B, 3E and 3F), like in *P*. *anchicaya*, *P*. *bilobulatus*, *P*. *molanoi* Floriano and Moreira, **sp. nov.**, *P*. *peruvianus*, *P*. *shuar*, *P*. *spiculus*, *P*. *sumaco*, *P*. *tridentatus*, *P*. *variabilis*, *P*. *vivatus*, *P*. *williamsi*, and *P*. *woytkowskii*. However, it differs from these species by the abdominal segment VIII without projections, but with an expansion posteriorly (Fig 3B, 3E and 3F). The female is characterized by the extension of abdominal sternum VII very large, completely covering the tergum VIII (Fig 3C and 3D).

**Geographic distribution (Fig 36).** COLOMBIA: **Antioquia** [6, this work], **Caldas** [this work], **Caquetá** [45], **Chocó** [45], **Córdoba** [this work], **Cudinamarca** [this work], **La Guajira** [this work], **Norte de Santander** [38, this work], **Putumayo** [38, this work], **Quindío** [44, 46–48, this work], **Risaralda** [49], **Santander** [38, this work], **Tolima** [38, this work], **Valle del Cauca** [30, this work]. VENEZUELA: **Barinas** [6, this work], **Zulia** [this work].

#### Type material examined

2♂, 2♀ paratypes (USNM): ‘COLOMBIA, Antioquia\ Rio Claro at Nature Res.\ 13 km. W. of Doradal\ 250 m., water temp. 25°C\ 21 July 1989 CL 2405\ D. A. & J. T. Polhemus’ ‘PARATYPE\ *Potamobates*\ *carvalhoi*\ J & D Polhemus’. 2♂, 2♀ paratypes (USNM): ‘Barinitas\ Dec. Venez\ P. Anduze’ ‘C J Drake\ Coll. 1956’.

#### Additional material examined

1♀, 1♂, 3 nymphs (EQ): ‘Analquin PMA\ Campo Matachines\ P5 Q. [] la mata.\ Nestor\ / *Potamobates*\ *carvalloi*. 2♀, 5♂, 12 nymphs (EQ): ‘Rio Tarra\ Concol Peq [Proyecto e-Qual]\ E13\ neuston\ *Potamobates*\ *carvalhoi*\ 12N 7A. 1♀, 1♂ (EQ): ‘Ecoforest PEQ [Proyecto e-Qual] 35\ E [Estación] 17 Q. [Quebrada] La Puna\ 27/01/2013 Neuston\ *Potamobates*\ *carvalloi*. 6♀, 6♂, 1 nymph (EQ): “Gerridae\ *Potamobates carvalloi* \ Q.seca/Concol PEQ [Proyecto e-Qual] 15\ E37 Neuston\ C12’. 4♀, 4♂, 3 nymphs (EQ): ‘Gerridae P7 Neuston\ *Potamobates carvalloi* \ Q.la Arenosa\ Analquin PMA\ Campos matachines\ (q)’. 1♀, 2♂, 8 nymphs (EQ): ‘Concol Peq 15\ 30/o7/12\ P8\ *Potamobates*\ *carvalloi*. 1♂ (EQ): ‘Corriente Pozo Dicha\ Cuenca Rio Magdalena\ Subcuenca Rio Saldaña\ Altura 450 msnm\ Município Ataco (Tolima)\ Colector HERNAN ARISTIZABAL G.’ ‘Muestra HA 336\ Hemiptera\ Heteroptera\ Gerridae\ *Potamobates peruvianus*\ Identifico: HAG’. 2♀, 2♂ (EQ): ‘Hemiptera #457\ Gerridae Q. Arrubla\ *Potamobates*. Ibagué\ 4 individuos y cuerpo J. botánico\ H. Aristizabal. 22/06/03’. 1♀, 1♂ (EQ): R. Cañaveral\ P12 CMTX)\ *Potamobates*\ *carvalloi*. 2♀, 2♂, 2 nymphs (EQ): ‘Gerridae\ *Potamobates carvalloi*\ Q. el tigre Analquim PMA\ Campo Matachines*\* P1 Neuston (6)’. 1♀, 1♂, 8 nymphs (EQ): ‘Gerridae P6 Neuston\ *Potamobates carvalloi*\ Q. Mapronal\ Analquim PMACampo\ Matachines (10)’. 4♀, 2♂, 16 nymphs (EQ): ‘Gerridae\ *Potamobates carvalohí*\ Q. El Turbaco–Neuston\ 30-Jul-12\ Pq (23)’. 5♀, 3♂, 1 nymph (EQ): ‘Q. la Palmara\ Ecoforest P EQ35\ 27-01-13\ Neuston\ *Potamobates*\ *carvalhoi* 8A 1N’. 1♂ (EQ): ‘Gerridae\ *Potamobates carvalohí*\ Caño Piñuelo Concol PEQ 15\ E23 (1)’. 3♀, 2♂, 13 nymphs (EQ): ‘Corriente Quebrada las Gallinas\ Cuenca Rio Sinú\ Subcuenta Alto Sinú\ Altura 70\ Município Tierralta (Córdoba)\ Colector HERNAN ARISTIZABAL G’ ‘Muestra HA 6\ Hemiptera Heteroptera\ Gerridae\ *Potamobates* sp 1\ Identifico HAG. 6♀ (EQ): ‘Curadica\ Concol Peq15\ E13 Neuston\ *Potamobates*\ *carvalloi* 6A’. 1♀, 1♂ (INVERTUN): Hemiptera\ Gerridae\ *Potamobates*\ Quebrada Padilla\ antes de carretera\ 20-X-99’. 6♀, 4♂, 1 nymph (INVERTUN): ‘Hemiptera\ Gerridae, *Pota*\ *mobates carvalhoi*\ R. Rancheria\ Ene– 2003\ Frente Acueducto\ Barrancas\ 400 m.s.n.m\ 10 individuos’. 1♀ (INVERTUN): ‘Corriente Quebrada Jagual\ Cuenca Rio Magdalena\ Subcuenca Rio Saldaña\ Altura 450 msnm\ Municipio Ataco (Tolima)\ Colector HERNAN ARISTIZABAL G’ ‘Muestra HA 335\ Hemiptera Heteroptera\ Gerridae\ *Potamobates* sp. 1\ Identifico HAG’. 1♀ (INVERTUN): ‘Corriente Rio Sucio\ Cuenca Rio Magdalena\ Subcuenca Rio Guali\ Altura 1300 msnm\ Municipio Fresno (Tolima)\ Colector HERNAN ARISTIZABAL’ ‘Muestra HA 292\ Hemiptera Heteroptera\ Gerridae\ *Potamobates* sp. 1\ Identificó HAG’. 2♀, 1♂, 8 nymphs (INVERTUN): ‘Hemiptera\ Gerridae\ *Potamobates carvalhoi*\ Det. H. Laython.S’ ‘Rio Miel\ 5°42’31.8”N/ 74°44’4.7”W\ 15 Junio 2002/ 170 msnm\ Luego de R. As maná\ Dorada, Caldas\ Col: M. Laython.S’. 7♀, 8♂ (INVERTUN): ‘Rio Rancheria\ 10° 57’26.30”N\ 03/12/2003\ Antes de Rio Marocaso\ Guajira, Colombia\ Col: Marco Laython’ ‘Hemiptera\ Gerridae\ *Potamobates carvalhoi\* Det: M. Laython.S’. 1♀ (INVERTUN): ‘E10 Neuston’. 13♀, 8♂, 20 nymphs (INVERTUN): ‘Ene-2003\ R. Rancheria\ E7’. 15♀, 18♂, 52 nymphs (INVERTUN): ‘Ene-2003\ R. Rancheria\ E9’. 15♀, 15♂, 8 nymphs (INVERTUN): ‘Rancheria Ene 2003\ E1’. 1♀ (INVERTUN): ‘Rancheria\ Ene-2003\ E5’. 41♀, 23♂, 19 nymphs (INVERTUN): ‘Rancheria–Ene-2003\ E2’. 2♀, 2♂ (INVERTUN): ‘Ene-2003\ R. rancheria\ E7’. 1♀ e 4♂ (INVERTUN): ‘R. Rancheria\ Ene 2003\ E3’. 4♀, 7♂ (INVERTUN): ‘6 Neuston’. 3♀, 2♂, 6 nymphs (INVERTUN): ‘Rancheria. Zef 9\ *Potamobates carvalhoi*’. 2♀, 2♂, 4 nymphs (INVERTUN): ‘R. Marocaso; A.R. Rancheria\ Caracoli/Guajira 3-XII-03\ Neuston. M. Laython’. 2♀, 1 ♂ (UPTC): ‘Venezuela–La Zulia–Toromo\ Rio Negro Dec-31-2008\ 428 msnm col. L. Garcia’ ‘P. Ca. 0066’ ‘*Potamobates*\ *carvalhoi*’. 1♀ (UPTC): ‘Colombia–Quindio quinta\ Lugar ocaso\ Altura:\ Fecha 30/07/04\ Met. Cole.\ Colector: L. Garcia’. 1♀ (MUSENUV): ‘Colombia Valle\ Buga.\ 1000\ Hosp: red\ Fecha oct. 94\ Col. J. Amaya’ ‘Fila: Gerridae\ Subfila:\ Gen: *Limnogonus*’ ‘Sp:\ Det: M. Lozano.T.’ 2♀ (MUSENUV): ‘Colombia Tolima\ Valle de san Juan\ Altura 750 msnm\ Hosp: Red Acuatica\ Fecha: 14-08-99\ Col: L.M. Romero’ ‘Fila: Gerridae\ Subfila:\ Gen:\ Sp:\ Det. L.M. Romero’.

### *Potamobates horvathi* Esaki, 1926

(Figs 7 and 16)

*Potamobates horvathi* Esaki, 1926 [10]: 254–257 (description, figure). Drake and Harris (1928) [9]: 26 (note). Drake and Harris (1934) [21]: 224, 226, 227, 240, 241 (figure, key, redescription). Kuitert (1942) [17]: 140, 141 (key, note). Matsuda (1960) [2]: 512–515 (figure). Polhemus and Polhemus (1995) [6]: 358–360, 364, 366–368, 372 (figure, key, phylogeny, redescription). Cognato (1998) [18]: 21, 22 (phylogeny). Buzzetti (2006) [19]: 52, 53, 55 (figure, note, phylogeny). Padilla-Gil and Damgaard (2011) [7]: 44, 46 (key, phylogeny). Morales et al. (2013) [5]: 194 (figure).

*Potamobates unidentatus*; Molano et al. (2005) [50]: 169 (list), (2008) [30]: 45, 55 (habitat, records); *non* Champion, 1898 (misidentifications).

*Potamobates bidentatus*; Posso and González (2008) [31]: 232 (records); *non* Champion, 1898 (misidentification).

*Potamobates tumaquensis* Padilla-Gil and Damgaard, 2011 [7]: 41–49 (description, figure, key, phylogeny) (**syn. nov.**).

#### Diagnosis

Anterior region of propleuron without patch of golden setae; male pygophore and proctiger sinistrally rotated about 30° (Fig 7A, 7B, 7E and 7F); left ventral margin of male abdominal segment VIII with a single projection, projection length subequal to width (Fig 7B and 7E); female abdominal tergum VIII subequal in length to mediotergite VII (Fig 7C); extension of female abdominal sternum VII triangular, clearly shorter than mediotergite VII (Fig 7F).

#### Redescription

Length (**♂**: 7.5–8.8; ♀: 8.0–8.4); width (**♂**: 1.8–2.0; ♀: 2.2–2.3 mm). *Head*: antennomere I subequal in length to head width, 2.2–2.4 times longer than antennomere II; II about 1.6 times longer than III; IV about 1.3 times longer than III. Eye width 1.3–1.8 times interocular width. Article III of labium 1.6–1.8 times longer than IV. Antenna black; dorsum of head yellow; frons with blackish mark; vertex without marks; antenniferous tubercle black; mandibular and maxillary plates yellowish; labium with articles I and II yellow, III and IV blackish; venter of head yellowish. *Thorax*: Pronotum with yellowish median stripe, posterior margin not covered by golden setae; anterodorsal region of propleuron without patch of golden setae; proacetabulum yellow, with dorsolateral stripe of golden setae and ventral brownish to blackish mark; fore coxa and trochanter yellowish, trochanter with brownish distal margin; fore femur with distal 1/4 of dorsum and entire venter blackish; fore tibia and tarsus blackish. Mesonotum with yellowish median stripe occupying 1/10 to 7/10 of its length; posterior margin without golden setae; dorsal 3/4 of spiracle covered by golden setae; mesopleuron with longitudinal stripe of golden setae, stripe sigmoid, slightly curved, posteriorly discontinue; mesosternum with blackish mark anteriorly below proacetabula; middle and hind coxae yellowish; middle and hind trochanters brownish, ventrally with apex yellowish; middle and hind femora, tibiae, and tarsi black. Metanotum without stripes; posterior margin with golden setae; metacetabulum with stripe of golden setae laterally. *Abdomen*: medio- and laterotergites black, lateral margins of mediotergites with longitudinal stripe of golden setae; side of abdomen black; sterna without marks. *Male*: abdominal sterna without depressions; posterior margin of abdominal mediotergite VII rounded (Fig 7A). Posterior projection of last abdominal laterotergite (= connexival spine) with apex rounded (Fig 7A). Abdominal sternum VII with oval notch on posterior margin, notch almost three times as wide as long. Abdominal segment VIII tubular, 2–3 times as long as wide, without ventral depression; lateral margins slightly tapering posteriorly, unmodified; anterior width 2–3 times posterior width; posterodorsal margin rounded; posteroventral margin with lateral triangular projection (Fig 7B, 7E and 7F). Pygophore and proctiger sinistrally rotated about 30° (Fig 7A, 7B, 7E and 7F). Pygophore 2.1–2.4 times longer than abdominal segment VII. Proctiger 1.8–2.0 times longer than abdominal segment VII; anterior margin with short notch, notch with about 1/5 of length of proctiger; right margin not folded ventrally; left margin folded only on left basolateral process; apex with many black denticles; right basolateral process not developed; left basolateral process with length 4/5 to subequal to its width, with 1/5 of length of proctiger, longitudinal axis diverging about 45° from longitudinal axis of proctiger, lateral margins converging to apex, apex not bifid (Fig 7J). Phallus length about 1/3 of smallest width (Fig 7G); dorsal sclerite slightly longer and narrower than ventral sclerite, base not notched (Fig 7H); lateral sclerite rectangular; ventral sclerite centrally notched, base bifid, apex almost twice as wide as center (Fig 7I); transverse sclerite large, folded over ventral sclerite (Fig 7G). Abdominal segment VIII dorsally black, ventrally light-yellow; pygophore brownish to black, right ventral margin brown, left ventral margin yellowish; proctiger black, left basolateral process brown. *Female*: abdominal laterotergites folded over mediotergites on segments V–VII (Fig 16C); posterior projection of last abdominal laterotergite (= connexival spine) absent (Fig 7C and 7D). Abdominal sternum VII with posterior margin rounded (Fig 7D). Extension of sternum VII triangular, about twice as wide as long, partially covering first pair of gonocoxae, not folded over tergum VIII (Fig 7D). Abdominal tergum VIII triangular, length 3/5–4/5 of width; lateral margins strongly converging posteriorly; posterior margin rounded (Fig 7C). Anal cone long, slightly shorter than abdominal tergum VIII (Fig 7C and 7D). Abdominal segment VIII dorsally black with brown lateral margins, ventrally yellowish; anal cone black dorsally. *Macropterous*: posterolateral 1/3 and posterior margin of pronotum yellowish.

#### Variation

Blackish mark on frons small in some specimens; larger and extended between eyes in others, leaving just small yellow mark on vertex. In such darker specimens, pronotal stripe narrower and mesonotal stripe can be much reduced. Venter of fore trochanter can be light-brown, dorsum of fore femur yellow with distal margin to distal half black. Posterolateral margin of mesonotum usually with tuft of setae; posterolateral 1/3 of mesosternum can have brownish mark. Middle and hind trochanters brown with apex yellowish to black. Apex of middle and hind femora, tibiae, and tarsi can be brownish. Male can have base and apex of abdominal tergum VIII yellowish. Margins of the last three female abdominal laterotergites can be more produced mesally and yellow.

#### Comments

*Potamobates horvathi* has the male pygophore and proctiger weakly rotated, never reaching 45°, like in *P*. *bidentatus*, *P*. *manzanoae*, *P*. *osborni*, and *P*. *unidentatus*. The females of *P*. *horvathi* and *P*. *unidentatus* differ from *P*. *bidentatus*, *P*. *manzanoae*, and *P*. *osborni* by the posterior margin of abdominal tergum VIII rounded, while it is acute and elongated in the other species (Figs 8C, 8D, 15C and 23C). The female of *P*. *horvathi* can be easily distinguished from *P*. *unidentatus* based on the yellow median stripe present on the pronotum, while the latter has a wedge-shaped mark. Padilla-Gil and Damgaard (2011) [34] described *P*. *tumaquensis* and argued that it would be very similar to *P*. *horvathi*, from which it would differ because the left basolateral process of the male proctiger is exposed. Aristizábal-García (2017) [1] suggested that both species would be synonyms, but did not include any rationale for this proposal. After examining hundreds of specimens of *P*. *horvathi*, we concluded that the position of the left basolateral process of the proctiger in relation to the body is variable. During copulation, the proctiger is distended posterodorsally, leaving the left basolateral process exposed, and the pygophore is dislocated posteroventrally, allowing the phallus to extend out of the genital capsule. Therefore, the difference between *P*. *horvathi* and *P*. *tumaquensis* presented by the authors of the latter species is not consistent, and we consider that both are indeed synonyms, as originally suggested by Aristizábal-García (2017) [1]. The left basolateral process of the proctiger as described for *P*. *tumaquensis* has a small posterior curve, but this is a variation that can be seen within populations of *P*. *horvathi*, in which some males have this curve, while other have the process straight. Finally, the authors of *P*. *tumaquensis* mentioned in their key that it would have a wedge-shaped mark on the pronotum, however, both the illustration presented together with the description and the holotype that we had acces to show an elongated mark, exactly like in *P*. *horvathi*.

**Fig 38:**
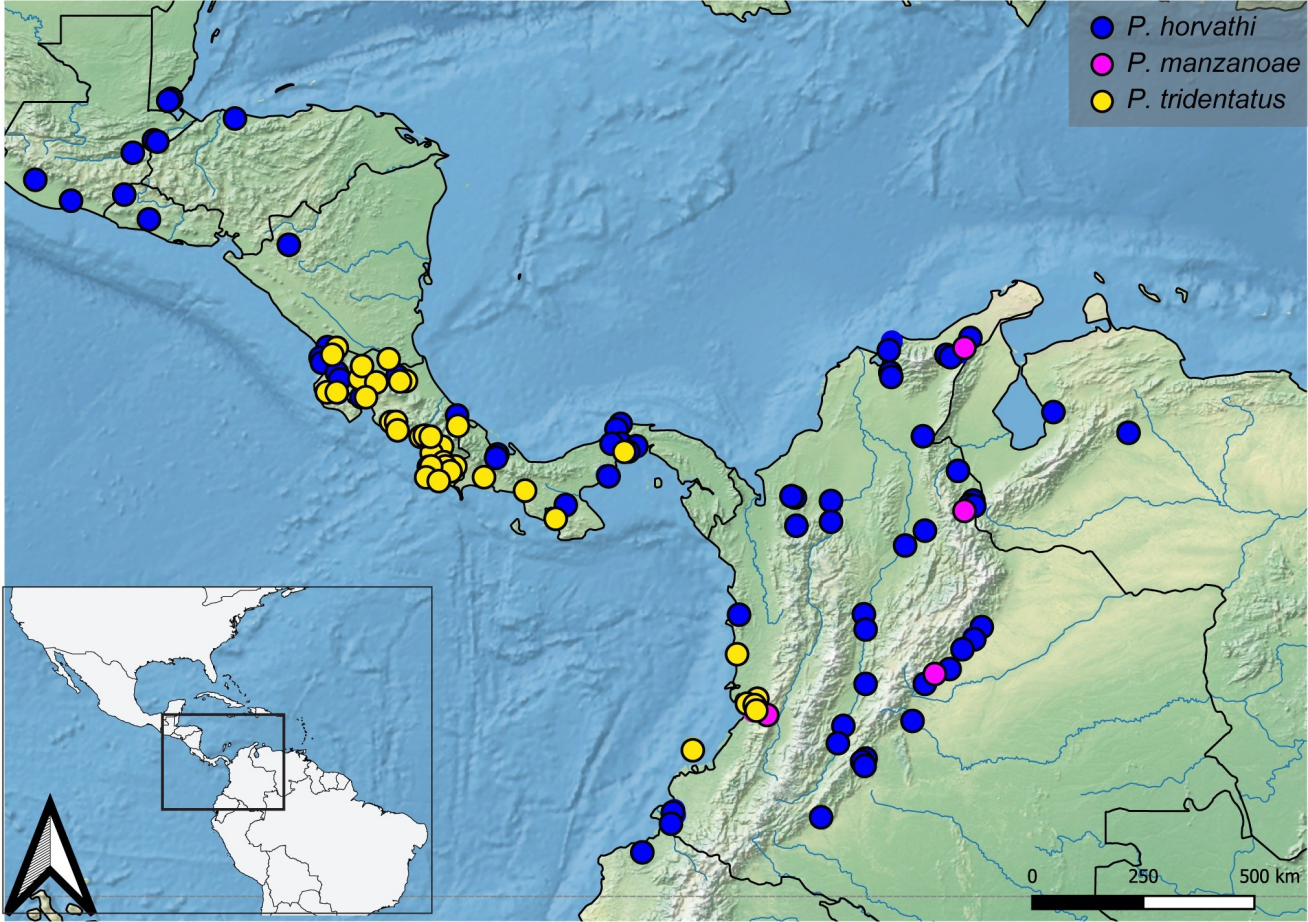
Map showing the geographical distribution of species of *P*. *horvathi*, *P*. *manzanoae*, *P*. *tridentatus*. Spatial data from Natural Earth (http://www.naturalearthdata.com/).

**Geographic distribution (Fig 38).** BELIZE: **Toledo** [6, 14, 17, this work]. COLOMBIA: **Antioquia** [45], **Caldas** [36, 37, this work], **Caquetá** [1, 38, this work], **Casanare** [38, this work], **Cesar** [45], **Chocó** [45], **Córdoba** [38, this work], **Cundinamarca** [this work], **Huila** [38, this work], **La Guajira** [38, this work], **Magdalena** [10, 38, this work], **Meta** [1, 30, 47, this work], **Nariño** [7, 34, this work], **Norte de Santander** [38, this work], **Santander** [1, 38], **Tolima** [38], **Valle del Cauca** [6, 31, this work]. COSTA RICA: **Alajuela** [33], **Guanacaste** [6, 33, this work], **Limón** [33], **Puntarenas** [6, 33, this work]. ECUADOR: **Esmeraldas** [19]. EL SALVADOR: **San Salvador** [this work]. GUATEMALA: **Escuintla** [6, 9, this work], **Izabal** [6, 17, this work], **Jutiapa** [this work], **Suchitepéquez** [this work], **Zacapa** [6, this work]. HONDURAS: **Atlántida** [6, 14, this work]. MEXICO: **Chiapas** [17]. NICARAGUA: **Estelí** [6, this work]. PANAMA: **Bocas del Toro** [6], **Chiriquí** [6], **Colón** [6, 51, this work], **Herrera** [6], **Panama** [6, 14, 17, this work], **Veraguas** [52]. VENEZUELA: **Portuguesa** [10], **Zulia** [this work].

#### Type material examined

1♂ holotype, 1♀ allotype (ICN): ‘*Potamobates*\ *tumaquensis*\ Padilla- Gil & Dam-\gaard’ ‘Tumaco Vda sta\ Rosa, Consejo\ Rio Mejicano\ 6 feb 2009\ D. N. Padilla, leg’.

#### Additional material examined

2♀, 2♂ (LACM): ‘Punta Gorda\ Br. Honduras\ Nov 1931’ ‘W240’ ‘L J Muchmore’. 1♂ (NHRS): ‘Punta gorda\ Br. Honduras\ Nov 1931’. 1 ♂ (NHRS): ‘Punta gorda\ Br. Honduras\ Nov 1931’ ‘*Potamobates*\ *horvathi*\ D & H Esaki’. 1♀ (LACM): ‘Punta gorda\ Br. Honduras\ Nov 1931’ ‘Drake\ *Potamobates*\ *horvathi*\ Esaki’ ‘W240’ ‘L J Muchmore’. 1♀ (UCMC): ‘San Carlos\ PANAMA CL\ 1300 I-4-1970\ J. T. Polhemus’ ‘*Potamobates*\ *horvathi*. 1♂ (UCMC): ‘San Carlos\ PANAMA CL\ 1300 I-4-1970\ J. T. Polhemus’, 1♀, 1♂ (UPTC): ‘Colombia Córdoba Tierralta\ PNN Paramillo Alto rio\ Manso Sector Zancon\Trampa de Luz II.VI.2009\ Col. Carvajal, J.’ ‘*Potamobates horvathi*’. 1♀, 1♂ (Bernald): ‘Costa Rica, Puntarenas\ Osa, Rio Esquinas\ Finca Limón\ 26-02-08’ ‘Gerridae\ *Potamobates*\ *horváthi*\ Pet. B. Pacheco’. 1♀ (EQ): ‘Hemiptera #437\ Gerridae\ *Potamobates horvathi*\ R. meta, Q. Medano\ Chartie cusiana, aguazul\ 30/11/00\ H Aristizabal’. 7♂, 9♀, 6 nymphs (EQ): ‘Ecoforest Peq 35\ E14 cap. Paguey\ 28/10/13 Neuston\ *Potamobates*\ *horvathi*’. 5♀, 7♂, 2 nymphs (EQ): ‘P3 (RR3) (n)\ R. Rancheria\ 12-5-10\ Hemiptera\ Gerridae\ *Potamobates* cf. *horvathi*’. 6♀, 3♂, 5 nymphs (EQ): ‘Corriente Caño La Niata\ Cuenca Rio Orinoco\ Subcuenca Rio Meta\ Altura 380 msnm\ Municipio El Yopal (Casanare)\ Colector Hernan Aristizabal’ ‘Muestra HA 127\ Hemiptera Heteroptera\ Gerridae\ *Potamobates horvathi*\ Identifico: HAG’. 5♀, 4♂, 5 nymphs (EQ): ‘Corriente Caño La Niata\ Cuenca Rio Orinoco\ Subcuenca Rio Meta\ Altura 380 msnm\ Municipio El Yopal (Casanare)\ Colector HERNAN ARITIZABAL’ ‘Muestra HA127\ Hemiptera Heteroptera\ Gerridae\ *Potamobates horvathi*\ Identifico HAG’. 5♂, 2 nymphs (EQ): ‘Gerridae\ *Potamobates horvathi*\ R. rancheria\ 21/05/10\ P4\ (7)’. 2♀, 1♀ (EQ): ‘La vialidad\ PEQ10\ R. Pamplona, EST 1.\ Neuston 25/05/12\ *Potamobates*\ *horvathi*’. 1♀, 1♂, 1 nymph (EQ): ‘Gerridae\ *Potamobates horvathi*\ Q. Tigre aguas\ 4-05-11/ P02\ (3)’. 1♀ (EQ): P–16\ Caño Macapay\ Neuston\ Hemiptera\ Gerridae\ 1 individuo’. 1♀, 2 nymphs (EQ): ‘R. Tigre abajo\ 24-09-12\ k2 PEQ 27\ Neuston\ *Potamobates horvathi*’. 1♀, 2 nymphs (EQ): ‘Q. tigre médio\ 24-04-12\ peq-27\ *Potamobates*\ *horvathi*’. 2♀ (EQ): ‘Gerridae\ *Potamobates horvathi*\ Caño San Lorenzo\ AM 30 25/04/11\ (3)’. 2♀ e 2♂ (EQ): ‘P7 (Pq2) CN\ Arroyo La Quebrada\ 22-5-10\ Hemiptera\ Gerridae\ *Potamobates* cf. *horvathi*’. 8♀, 2♂, 3 nymphs (EQ): ‘Corriente Quebrada Urrá\ Cuenca Rio Sinú\ Subcuenca Alto Rio Sinú\ Altura 70\ Municipio Tierralta (Córdoba)\ Colector HERNAN ARISTIZABAL G’ ‘Muestra HA5\ Hemiptera Heteroptera\ Gerridae\ *Potamobates horvathi*\ Identifico: HAG’. 1♀, 3♂ (EQ): ‘Caño San Antonio\ Neuston AM– 40\ Gerridae\ *Potamobates*\ *horvathi* 4A’. 15♀, 4♂, 40 nymphs (EQ): ‘Gerridae\ *Potamobates horvathi*\ Guajira, R. Rancheria\ (RR1) 21-05-10 P1 (N)\ (57)’. 2♀, 1♂, 29 nymphs (INVERTUN): ‘Curriente Rio Aracataca\ Cuenca Ciénaga Grande de S.M\ Subcuenca Rio Aracataca\ Altura 40 msnm\ Municipio Aracataca (Magdalena)\ Colector HERNAN ARISTIZABAL G’ ‘Muestra HÁ 133\ Hemíptera Heteroptera\ Gerridae\ *Potamobates horvathi*\ Identificó: HAG’. 9♀, 4♂, 1 nymph (INVERTUN): ‘Corriente Las Perlas\ Rio Amazonas\ Rio Pato–Rio Caguán\ Altura 1200 msnm\ Municipio S. Vicente del Caguán (Caq.)\ Colector Hernan Aristizabal’ ‘Muestra HA339’ ‘Hemiptera Heteroptera\ Gerridae\ *Potamobates horvathi*\ Identificó: HAG’. 1♂, 2 nymphs (INVERTUN): ‘Corriente Quebrada La Floresta\ Cuenca Lago Maracaibo\ Subcuenca Rio Zulia\ Altura 300 msnm\ Municipio Cúcuta (N. Santander)\ Colector HERNAN ARISTIZABAL G’ ‘Muestra HA98\ Hemiptera Heteroptera\ Gerridae\ *Potamobates horvathi*\ Identifico HAG’. 1♀, 1♂ (INVERTUN): ‘Corriente sin dados de colección\ Cuenca\ Subcuenca\ Altura\ Municipio\ Colector HERNAN ARISTIZABAL’ ‘Muestra HA NN\ Hemiptera Heteroptera\ Gerridae\ *Potamobates horvathi*\ Identificó HAG’. 1♂ (INVERTUN): ‘Muestra HA 07\ Hemiptera Heteroptera\ Gerridae\ *Potamobates horvathi*\ Identifico HAG’. 2♀, 2♂ (INVERTUN): ‘Corriente Caño Garagoa\ Rio Orinoco\ Subcuenca Rio Cusiana\ Altura 800 msnm\ Municipio Agua Azul (Casanare)\ Colector Hernan Aristizabal G’ ‘Muestra HA302\ Hemiptera Heteroptera\ Gerridae\ *Potamobates horvathi*\ Identifico HAG’. 5♂ (INVERTUN): ‘Corriente Rio Pato\ Cuenca Rio Amazonas\ Subcuencas Rio Pato–Rio Caguán\ Altura 750 msnm\ Municipio S. Vicente Del Caguán (Caq.)\ Colector HERNAN ARISTIZABAL G’. 2♀, 5♂, 7 damaged specimens (INVERTUN): ‘Corriente Quebrada Cruz Grande\ Cuenca Rio Sinú\ Altura 70\ Municipio Tierralta (Córdoba)\ Colector HERNAN ARISTIZABAL G’ ‘Muestra HA\ Hemiptero Heteroptera\ Gerridae\ *Potamobates horvathi*\ Identifico HAG’. 8♀, 7♂ (INVERTUN): ‘Hemiptera\ Gerridae\ *Potamobates*\ *horvathi*\ Guajira. Ranche\ ria Ene-2003\ 300 msnm\ 14 indivíduos’. 8♀, 16♂, 7 damaged specimens (INVERTUN): ‘Corriente Rio Fundación antes\ Cuenca Ciéga Grande de S. M.\ Subcuenca Rio Fundación\ Altura 26 msnm\ Municipio Fundación (Magdalena)\ Colector HERNAN ARISTIZABAL G’ ‘Muestra HA142\ Hemiptera Heteroptera\ Gerridae\ *Potamobates horvathi*\ Identificó HAG’. 1♀ (INVERTUN): ‘Curriente Rio Zulia\ Cuenca Lago Maracaibo\ Subcuenca Rio Zulia\ Altura 200 msnm\ Municipio Cúcuta (N. Santander)\ Colector HERNAN ARISTIZABAL’ ‘Muestra HA120\ Hemiptera Heteroptera\ Gerridae\ *Potamobates horvathi*\ Identificó HAG’. 13 ♀, 8♂, 3 nymphs (INVERTUN): ‘Corriente Arroyo Bruno\ Cuenca Mar Caribe\ Subcuenca Rio Rancheria\ Altura 60 msnm\ Municipio Maicai (Guajira)\ Colector Hernan Aristizabal G’ ‘Muestra HA208\ Hemiptera Heteroptera\ Gerridae\ *Potamobates horvathi*\ Identifico HAG’. 1♀ (INVERTUN): ‘Corriente Quebrada Gaitá\ Cuenca Rio Sinú\ Subcuenca Alto Rio Sinú\ Altura 70\ Municipio Tierralta (Cordiba\ Colector HERNAN ARISTIZABAL G’ ‘Muestra HA2\ Hemiptera Heteroptera\ Gerridae\ *Potamobates horvathi*\ Identifico HAG’. 2♀ (INVERTUN): ‘Corriente Rio Magdalena\ Cuenca Rio Magdalena\ Subcuenca Alto Rio Magdalena\ Altura\ Municipio Sin Datos\ Colector Hernan Aristizabal G’ ‘Muestra HA 49\ Hemiptera Heteroptera\ Gerridae\ *Potamobates horvathi*\ Identifico HAG’. 2♀, 2♂, 11 nymphs (INVERTUN): ‘Rio Miel \ 50°42’31.8”N/ 74°44’4.7 W\ 15 Junio 2002/ 160 msnm\ Luego de R. Samaná\ Dorada, Caldas\ Col. H. Laython.s’ ‘Hemiptera\ Gerridae\ *Potamobates horvathi*\ Det: H. Laython.s’. 8♀, 5♂, 5 nymphs (INVERTUN): ‘Rio Rancheria 10°57’26.30”N 73°3’53.22”O\ 03/12/2003 450 msnm\ Antes del Rio Marocaso\ Guarija, Colombia\\ Col. Marco Laython’ ‘Hemiptera\ Gerridae\ *Potamobates horvathi*\ Det: M. Laython.s’. 1♀, 2♂ (INVERTUN): ‘E15 Neuston’. 4♀, 2 nymphs (INVERTUN): ‘E13 Neuston’. 45♂, 82♀, 37 nymphs (INVERTUN): ‘R. Rancheria Ere-2003\ E8’. 21 ♀, 2♂, 6 nymphs (INVERTUN): ‘R. Rancheria Ene 2003\ E15’. 8♀, 4♂, 3 nymphs (INVERTUN): ‘Ene 2003\ Rancheria ref 10\ *Potamobates horvathi*’. 3♀, 2♂ (INVERTUN): ‘Ene-2003\ R. Rancheria\ E7’.5♀, 5♂ (INVERTUN): ‘R. rancheria\ Ene-2003\ E3’. 42♀, 34♀, 62 nymphs (INVERTUN): ‘R. Rancheria Ene 2003\ E13’. 7♀, 10♀, 72 nymphs (INVERTUN): ‘R. Rancheria Ene-2003\ E12’. 1♀ (INVERTUN): ‘E12\ Neuston’. 15♀, 14♂, 8 nymphs (INVERTUN): ‘E8\ Neuston’ ‘R. Rancheria. Ac. Distracción\ Chomem/ Gueime 2-XII-03 260msnm\ Gerridae–*Potambotes horvathi*\ M. Taylor’. 2♀, 5♂ (INVERTUN): ‘Ene 2003 Ranche\ E10’. 24♀, 11♂ (INVERTUN): ‘R. Rancheria Ene 2003\ E 11’. 4♀, 2♂ (UPTC): ‘Colombia Cordoba Tierralta PNN\ Paramillo Alto rio Manso\ Sector Zancon\ trampa de Luz 11.vi.2009\ Col. Carvajal J.’ ‘*Potamobates*\ *horvathi*’ ‘P. ho. 0055’. 1♀ (UPTC): ‘Colombia Magdalena\ Cienaga corr.\ Cordobita Desembocadura\ Rio Toribio\ 4–V–2007\ Col. Jimenea L.’ ‘P. ho. 0056’ ‘*Potamobates*\ *horvathi*\ Det. Morales I’. 1♀, 1♂ (UPTC): ‘Colombia-Cordoba-Tierralta\ Vda Tuis-Tuis\ 13-XII-2006\ Col: Bernan V. ‘P. ho. 0057’ ‘*Potamobates*\ *horvathi*’. 3♀, 2♂ (UPTC): ‘Colombia Meta Granada\ Lugar rio Ariari\ V. Coguivocao\ Altura:\ Fecha 11/01/04\ Col. C. Upogui’ ‘Gen: *Potamobates*\ Esp: *horvathi*\ Det: F. Molano’ ‘P. ho. 0059’. 3♀ (UPTC): ‘Colombia Caqueta. Floren\ Lugar: Q. El dedo\ Altura 450 msnm\ Fecha: 01/03/03’ ‘Col: C. Serrato’ ‘P. ho. 0060’ ‘Gen: *Potamobates*\ Esp: *horvathi\* Det: D. Camacho’. 3♀, 2♀, 7 nymphs (UPTC): ‘Colombia Meta Granada\ Lugar: Caño Roja\ V. Coguivocoa\ Altura:\ Fecha: 11/01/04\ Col: C. Upogui’ ‘Gen: *Potamobates*\ Esp: *horvathi*\ Det: F. Molano’ ‘P. ho. 0061’. 2♀, 2♂ (UPTC): ‘Colombia Valle Bventu.\ Lugar: San Cipriano\ Qda. San Cipriano\ Alutura:\ Fecha: 14/06/05\ Col: J. Rivera’ ‘Gen: *Potamobates*\ Esp: *horvathi*\ Det: F. Molano’ ‘P. ho. 0062’. 1♀, 1♂ (UPTC): ‘Costa Rica. Qda\ Bar budal. Palo\ Verde. 16.01.08.\ Col. B. Pacheco’ ‘P. ho. 0063’ ‘Gerridae\ *Potamobates*\ *horvathi*\ Det. B. Pacheco’. 4♀, 1♂ (UPTC): ‘Colombia-Caqueta\ Lugar: Florencia\ Altura:\ Fecha 23/03/03\ Col: C. Serrato’ ‘Gen: *Potamobates*\ Esp: *horvathi*\ Det: F. Molano’ ‘P. ho. 0064’. 1♀ e 1♂ (UPTC): ‘Colombia Bventu.\ Lugar: Rio\ Altura:\ Fecha: 10/01/92\ Col: A. Galindo’ ‘Gen: *Potamobates*\ Esp: *horvathi*\ Det: A. Galindo’ ‘P. ho. 0065’. 1♀ (MUSENUV): ‘Colombia—Meta\ Serraría La Macarena\ Paitas 600 m.s.m\ Água turbia color verdoso\ Zona abierta\ C. Murillo sept. 14 1989’ ‘Gerridae\ *Potamobates*\ *unidentatus*\ F. Molano’. 5♀, 6♂ (MUSENUV): ‘Colombia—Valle\ Ca. Buenaventura\ Cordoba 96 m\ Margendel Río\ Enero 15\ 92\ A. Galindo’ ‘Gerridae\ *Potamobates*\ *horvathi*\ Fredy Molano’ ‘F2422’. 6♀, 2♂, 5 nymphs (MUSENUV): ‘Colombia—Valle\ Buenaventura—San\ Cipriano—Rio San\ Cipriano\ Agosto 7/ 89\ M.R. Manzano’ ‘Gerridae\ *Potamobates*\ *bidentatus*\ Fredy Molano’ ‘Réplica\ Dr. Polhemus\ Nov 1989’. 9♀, 5♂ (MUSENUV): ‘Q. Acuaclara\ Superfície\ abril 15/92\ F. Gerridae\ *Limnogonus*’. 1♀ (MUSENUV): ‘Rio Acuaclara\ (Superfície)\ abril 13/ 92\ F\ Gerridae\ *Trepobates*. 1♀, 2♂ (MUSENUV): ‘Q. Bortolo\ (Fundo)\ abril/6/96\ F. Gerridae’. 1♀, 1♂ (MUSENUV): ‘P. E/ Guineo\ (Superfície)\ abril 12/ 92\ F. Gerridae\ *Trepobates* sp’. 6♀, 5♂ (USNM): ‘Costa Rica, N. of\ Esparta, CL1264\ XII-24-1969\ J.T. Polhemus’ ‘J. T. Polhemus\ Collection 2014\ C.J. Drake Accession’. 7♀, 1♂ (USNM): ‘Nicaragua\ N. of Esteli\ CL1262, XII-23-69\ J.T. Polhemus’ ‘J. T. Polhemus\ Collection 2014\ C.J. Drake Accession’. 6♀, 3♂ (USNM): ‘Nicaragua: N. of\ Esteli, CL1262\ XII.23.1969\ J.T. Polhemus’ ‘J. T. Polhemus\ Collection 2014\ C.J. Drake Accession’. 10♀, 9♂ (USNM): ‘Panama, E. of\ Panama City\ CL1295, I-2-1970\ J.T. Polhemus’ ‘J. T. Polhemus\ Collection 2014\ C.J. Drake Accession’. 3♀, 6♂ (USNM): ‘Panama\ N. of Portobello\ CL1296, I-2-70\ J.T. Polhemus’ ‘J. T. Polhemus\ Collection 2014\ C.J. Drake Accession’. 10♀, 18♂ (USNM): ‘San Carlos\ Panama, CL\ 1300 I-4-1970\J.T. Polhemus’ ‘J. T. Polhemus\ Collection 2014\ C.J. Drake Accession’. 4♀, 4♂ (USNM): ‘Colombia, Valle de Cauca\ Rio Tatabro, 7km. E. of\ Sabaletas, 100 m., 24°C.\ 30 July 1989 CL 2436\ D. A. & J. T. Polhemus’ ‘J. T. Polhemus\ Collection 2014\ C.J. Drake Accession’. 1♂ (USNM): ‘Ernesto Barrera y\ Harry Brailovsky, col.\ Cañas\ Rio Lagarto\ Prov. Puntarenas\ Costa Rica.\ 9-11-81’ ‘Colección del Instituto\ de Biologia, UNAM.\ México, D.F.’ ‘*P*. *horvathi*\ JTP Esaki’ ‘J. T. Polhemus\ Collection 2014\ C.J. Drake Accession’. 7♀, 13♂ (USNM): ‘Costa Rica\ 4 mi. N. La Cruz\ CL 1307, I-8-70\ J. T. Polhemus’ ‘J. T. Polhemus\ Collection 2014\ C.J. Drake Accession’. 7♀, 9♂ (USNM): ‘Guatemala\ S. of Esquintla\ CL1252 XII-20-69\ J. T. Polhemus’ ‘J. T. Polhemus\ Collection 2014\ C.J. Drake Accession’. 7♀, 1♂ (USNM): ‘7 mi N Zacapa\ Guatemala CL\ 1376 I-11-1970\ J.T.Polhemus’ ‘J. T. Polhemus\ Collection 2014\ C.J. Drake Accession’. 1♀ (USNM): ‘E. of Quirigua\ Guatemala, CL 1317, I-11-1970\ J.T.Polhemus’ ‘J. T. Polhemus\ Collection 2014\ C.J. Drake Accession’. 4♀ (USNM): ‘Nr.L.Atescatempa\ Guatemala, CL 1312\ I-10-1970\ J.T.Polhemus’ ‘J. T. Polhemus\ Collection 2014\ C.J. Drake Accession’. 1♀ (USNM): ‘Canal Zone\ Pan. 2-10-39\ C. J. Drake’ ‘C J Drake\ Coll. 1956’ ‘J.T. Polhemus\ Collection’ ‘*Potamobates*\ *horvathi*\ CJD Esaki’ ‘J. T. Polhemus\ Collection 2014\ C.J. Drake Accession’. 1♂ (USNM): ‘Punta Gorda\ Honduras’ ‘C J Drake\ Coll. 1956’ ‘J. T. Polhemus\ Collection 2014\ C.J. Drake Accession’. 1♀ (USNM): ‘Punta Gorda\ Br. Honduras\ Feb. 1932’ ‘C J Drake\ Coll. 1956’ ‘J.T.Polhemus\ Collection’ ‘J. T. Polhemus\ Collection 2014\ C.J. Drake Accession’. 1♀ (USNM): ‘Rio Grande\ British\ Honduras\ November, 1931’ ‘J. T. Polhemus\ Collection 2014\ C.J. Drake Accession’. 2♀ (USNM): ‘Panama City, Pan\ February 1939\ Carl J. Drake’ ‘C J Drake\ Coll. 1956’ ‘J. T. Polhemus\ Collection 2014\ C.J. Drake Accession’. 1♂ (USNM): ‘Punta Gorda\ Br. Honduras\ C.A. 1932\ J. J White’ ‘J.T. Polhemus\ Collection’ ‘*Potamobates*\ *horvathi*\ Kans. Esaki’ ‘J. T. Polhemus\ Collection 2014\ C.J. Drake Accession’. 1♀ (USNM): ‘Lan cetilha,\ Honduras\ 3-22-36 John Deal’ ‘♀’ ‘J.T. Polhemus\ Collection’ ‘J. T. Polhemus\ Collection 2014\ C.J. Drake Accession’. 1♀ (USNM): ‘Los Amates\ Guat 16-I-5’ ‘J.R.de la\ Torre-Bueno\ Collection K.U’ ‘*Potamobates*\ *bidentatus*\ KU\ Champ’ ‘J. T. Polhemus\ Collection 2014\ C.J. Drake Accession’. 2♀ (USNM): ‘J. T. Polhemus\ Collection 2014\ C.J. Drake Accession’. 2♀, 1♂ (USNM): ‘Escuintla\ Gta Ca 31-I-5’ ‘Ex coll. J.B. de la Torre bu–\eno June 1919’ ‘H.M. Parshley\ Collection’ ‘*Potamobates*\ *horvathi*\ Esaki\ det. J.T. Polhemus 1968’ ‘J. T. Polhemus\ Collection 2014\ C.J. Drake Accession’. 2♀, 1♂ (USNM): ‘Canal Zone\ Pan. 2-10-39\ C.J.Drake’ ‘J C Lutz\ Collection\ 1961’. 1♂ (USNM): ‘Alhajuelo\ Pan Apr 5.11\ August Busck’ ‘*Potamobates*\ Champ’. 1♀ (USNM): ‘Juan Diaz\ Panama\ II-2-1935’ ‘C J Drake\ Collection’ ‘*Potamobates*\ *unidentatus*\ Champ.’ ‘*Potamobates*\ *horvathi\* ♀ Esaki\ det. J. T. Polhemus’ ‘J. T. Polhemus\ Collection 2014\ C.J. Drake Accession’. 1♀, 3♂ (USNM): ‘Costa Rica, N. of\ Esparta, CL1264\ XII-24-1969’ ‘J.T.Polhemus’. 1♀ (USNM): ‘Punta Gorda\ Br. Honduras\ Feb. 1932’ ‘J C Lutz\ Collection\ 1961’. 1♀, 1♂ (USNM): ‘Punta Gorda\ Br. Honduras\ Nov. 1931’. 1♀, 3♂ (USNM): ‘Las Cruces\ CZ Pan\ II-4-11’ ‘Aug–Busck\ Collection’. 3♀ (USNM): ‘Venezuela\ Est. Zulia\ R. San Juan S.\ Mene Grande’ ‘L.P. Schultz\ Mar.20,(‘)42\ 11-42-110’. 1♀, 22 nymphs (USNM): ‘Costa Rica\ Guanacaste Prov.Liberia\ 27 March 1987\ J. M. Hill. 2♀, 1♂ (USNM): ‘Guanacaste\ Costa Rica\ 13 VII 57\ DR Lauck’ ‘C J Drake\ Coll. 1956’. 2♀, 2♂ (USNM): ‘San Salvador\ El Salvador\ 6 VII 57\ DR Lauck’ ‘C J Drake\ Coll. 1956’. 2♀, 4♂ (USNM): ‘Canal Zone\ Pan. 2-10-39\ C. J. Drake\ C J Drake\ Coll. 1959’. 6♀, 2♂ (USNM): ‘Punta Gorda\ Br. Honduras\ Nov. 1931’ ‘C J Drake\ Coll. 1956’. 1♀, 1♂ (USNM): ‘Punta Gorda\ Br. Honduras\ Feb. 1932’ ‘C J Drake\ Coll. 1956’. 1♀, 1♂ (USNM): ‘Mazatenango\ 3 Feb.05 Gta Ca’ ‘C J Drake\ Coll. 1956’. 1♂ (USNM): ‘Panama City Pan\ February 1939\ Carl J. Drake’ ‘C J Drake\ Coll. 1956’. 1♀ (USNM): ‘Venezuela\ est. Zulia\ Rio San Juan\ S. Mene Grande’ ‘L. P. Schultz\ Mar.20.1942\ 11-42-110’ ‘C J Drake\ Coll. 1956’. 1♀ (USNM): Barro Colorado\ C. Z. Panama\ Feb. 6-8-1939\ Carl J. Drake’ ‘C J Drake\ Coll. 1956’. 1♀ (USNM): ‘Columbia\ Ujhelyi’ ‘Aracataca\ 1912.II.’ Coll. Mus. Nat. Hung.’. 44♀, 37♂ (USNM): ‘Rio Grande\ British Honduras\ November. 1931’ ‘C J. Drake\ Coll. 1956’. 1♂ (AMNH): ‘L J Muchmore’ ‘Punta Gorda\ BR. Honduras\ Nov. 1931’ ‘W240’.

### *Potamobates manzanoae* Polhemus and Polhemus, 1995

**(Figs 23,** 25A, 25C, 25H and 25M**)**

*Potamobates manzanoae* Polhemus, 1995 [6]: 356, 357, 360, 364, 366–369, 372 (description, figure, key, phylogeny). Cognato (1998) [18]: 21 (phylogeny). Buzzetti (2006) [19]: 55, 56 (phylogeny). Padilla-Gil and Damgaard (2011) [7]: 44, 46–48 (key, phylogeny).

#### Diagnosis

Male pygophore and proctiger sinistrally rotated about 30° (Fig 25A, 25C and 25H); posterior lateroventral margin of male abdominal segment VIII with a single projection, projection subequal in length and width (Fig 25C); female abdominal tergum VIII twice as long as mediotergite VII, lateral margins abruptly converging on posterior 2/3 (Figs 23C, 25M and 25Q); extension of female abdominal sternum VII long, triangular, about twice as long as wide and slightly twisted, subequal in length to tergum VIII (Figs 23D, 25M and 25Q).

#### Comments

The male of *P*. *manzanoae* has the pygophore and proctiger sinistrally rotated by no more than 30°, similar to *P*. *bidentatus*, *P*. *horvathi*, *P*. *osborni*, and *P*. *unidentatus*. *Potamobates manzanoae* differs from *P*. *horvathi*, *P*. *bidentatus*, and *P*. *osborni* by the wedge-shaped mark on the pronotum (Fig 23A and 23C), while the other three species display a longitudinal stripe. The female of *P*. *manzanoae* can be distinguished from all congeners by the combination of lateral margins of abdominal tergum VIII converging abruptly on posterior 2/3 (Fig 23C) and extension of abdominal sternum VII triangular, about twice as long as wide and slightly twisted (Fig 23D).

**Geographic distribution (Fig 38).** COLOMBIA: **Cudinamarca** [36, 37], **La Guajira** [45], **Norte de Santander** [30], **Valle del Cauca** [6, this work].

#### Type material examined

4♀, 2♂ paratypes (USNM): ‘Colombia, Valle de Cauca\ swift rocky tributary\ to Rio Anchicaya, W. of Sabaletas, 100 m., 24°C.\ 30 July 1989 CL 2435\ D. A. & J. T. Polhemus’ ‘PARATYPE\ *Potamobates manzanoae*\ J.T.Polhemus\ &\ D. A. Polhemus’.

#### Additional material examined

1♀, 1♂ (MUSENUV): ‘Colombia—Valle\ Buenventura\ Quebrada después\ de R. água clara\ via a B. ventura\ M.R. Manzano\ Julio 30 de 1989’ ‘MRM Agua Clara 018’ ‘F2409’ ‘Gerridae\ *Potamobates*\ *tridentatus*\ F. Molano’.

### *Potamobates molanoi* Floriano and Moreira, sp. nov.

(Figs 18 and 29)

urn:lsid:zoobank.org:act:1A4CACD0-2546-40BD-8EB6-A9E3D1EA8A9C

*Potamobates vivatus*; Polhemus and Polhemus, 1995 [55]: 361–364, 367, 368, 372 (record); *non* Drake and Roze, 1954 [17] (specimens from Colombia; misidentification).

*Potamobates vivatus*; Aristizabal-Gárcia (2002) [38]: 92, 109–111 (key, figure, records); Aristizabal-Gárcia (2017) [1]: appendix, 73 (records); Padilla-Gil and Nieser (2003a) [36]: 61 (records), (2003b) [37]: 45 (records); Molano et al. (2008) [30]: 55 (habitat, record); Galindo-Malagón et al. (2009) [53]: 51 (records). Morales and Castro (2010) [45]: 276, 277 (records); Mondragón and Morales (2013) [32]: 15, 16 (records); *non* Drake and Roze, 1954 [10] (misidentifications).

#### Diagnosis

Male pygophore and proctiger sinistrally rotated about 90° (Fig 18A, 18D and 18E); right posterolateral margin of male abdominal tergum VIII with a ventral projection, projection subequal in length and width (Fig 18D and 18E); female abdominal tergum VIII almost 4 times as long as wide (Fig 18B and 18C); extension of female abdominal sternum VII short, about half as long as tergum VII (Fig 18C).

#### Description

Length (**♂**: 10.6; ♀: 11.5); width (**♂**: 2.7; ♀: 2.4) (Fig 29). *Head*: antennomere I 1.2 times longer than head width, 2.8–3.0 times longer than antennomere II; II 1.0–1.2 times longer than III; IV about 1.3 times longer than III. Eye width 1.3–1.5 times interocular width. Article III of labium 1.6–2.0 times longer than article IV. Antenna black, venter of antennomere I slightly lighter; dorsum of head yellowish, frons with brownish mark; antenniferous tubercle with apex black; mandibular and maxillary plates yellowish; labium with articles I–II and base of III yellowish, remainder of III and whole IV blackish; venter of head yellowish. *Thorax*: Pronotum with a yellowish wedge-shaped mark, posterior margin not covered by golden setae; propleuron with anterior mark of golden setae; proacetabulum yellowish, posterior margin brownish; fore coxa, trochanter, and femur yellowish, venter and apex of femora blackish; fore tibia and tarsus blackish. Mesonotum blackish; dorsal 3/4 of spiracle covered by golden setae; mesopleuron with longitudinal stripe of golden setae, stripe sigmoid, curved, posteriorly discontinue; mesosternum without marks; middle and hind coxae, trochanters, and femora yellow; middle and hind femora with black stripe dorsally and ventrally; middle and hind tibiae and tarsi brownish. Metanotum without stripes, posterolateral margin with golden setae; metacetabulum dorsally with longitudinal blackish stripe and stripe of golden setae laterally; metapleuron and metasternum yellowish. *Abdomen*: medio- and laterotergites I–VII black, lateral margins of mediotergites with longitudinal stripe of golden setae. *Male*: posterior margin of abdominal mediotergite VII rounded. Posterior projection of last abdominal laterotergite (= connexival spine) triangular (Fig 18A). Abdominal sterna II–VII with weak median depression, more conspicuous posteriorly. Abdominal sternum VII with a rounded notch on posterior margin, notch subequal in length and width. Abdominal segment VIII 1.6–1.8 times longer than wide, tubular, with lateral margins slightly divergent posteriorly, anterior width 4/5–9/10 of posterior width; right side with a wide, short (length subequal to basal width) projection directed posteroventrally, apex of projection rounded to acute (Fig 18D and 18E); venter with median depression anteriorly extending to about middle of segment, posteroventral margin with an indentation. Pygophore and proctiger sinistrally rotated about 90° (Fig 18A, 18D and 18E). Proctiger 1.3 times longer than abdominal mediotergite VII; anterior margin with a long notch, notch with about 1/3 of proctiger length; right and left margins not folded ventrally; apex without denticles; right basolateral process developed, rounded, with less than 1/5 of left basolateral process length; left basolateral process long (about 1.8 times longer than wide, length about 3/5 of proctiger length), longitudinal axis diverging about 100° from longitudinal axis of proctiger, lateral margins converging distally, apex with acute angle, not bifid. Phallus long, about 6.25 times longer than smallest width; dorsal sclerite anteriorly coiled, slightly narrower and much longer than ventral sclerite, base not notched; lateral sclerite triangular; ventral sclerite with base bifid, apex slightly wider; transverse slecrite not visible. Abdominal tergum VIII black, with lateral margins yellowish; sternum VIII and pygophore yellowish, apex brownish; proctiger dark-brown. *Female*: Abdominal tergum VIII triangular, almost 4 times as long as wide, posterior margin acute (Fig 18B); lateral margins strongly divergent posteriorly; anal cone short to long, when long, subequal in length to mediotergite VII. Abdominal laterotergites not folded over mediotergites (Fig 29C); last laterotergite with a posterior spine, spine tubular, long, 1.3 times longer than mediotergite VII, apex rounded (Fig 18B and 18C). Extension of female abdominal sternum VII oval, about twice as wide as long, with an oval lobule, completely covering first pair of gonocoxae, not folded over tergum VIII (Fig 18C). Abdominal tergum VIII and extension abdominal of sternum VII yellowish; base of tergum VIII with a blackish mark centrally, posterior 2/3 blackish. *Macropterous*: Posterolateral 1/2 and posterior margin of pronotum yellowish.

#### Variation

The fore trochanter can have a brown median mark ventrally. The mesonotum can have a yellowish median stripe posteriorly, two yellowish marks laterally on posterior third, and the posterior margin with golden setae.

#### Etymology

This species is dedicated to our friend, the late Dr. Fredy Molano (UPTC), who made many contributions to the understanting of the Colombian semiaquatic bugs.

#### Comments

The male of *P*. *molanoi* Floriano and Moreira, **sp. nov.** has the pygophore and proctiger sinistrally rotated about 90° (Fig 18A, 18D and 18E), similar to *P*. *anchicaya*, *P*. *bilobulatus*, *P*. *carvalhoi*, *P*. *peruvianus*, *P*. *shuar*, *P*. *spiculus*, *P*. *sumaco*, *P*. *tridentatus*, *P*. *variabilis*, *P*. *williamsi*, and *P*. *woytkowskii*. It differs from all these species by the ventral projection on the right posterolateral margin of abdominal segment VIII, which is subequal in length and width (Fig 18D and 18E). This new species is similar to *P*. *vivatus*, and specimens from Colombia have been confused with it in the literature [1, 16, 35, 36, 38, 50]. The new species can be distinguished from *P*. *vivatus* mainly by the position of the posterolateral projection of male abdominal segment VIII, which is ventral in the former (Fig 18D and 18E), but dorsal in the latter (Fig 27B). Some specimens from Colombia that we examined vary in the shape of the projection, but never in the position of insertion. In addition to this character, *P*. *molanoi* Floriano and Moreira, **sp. nov.** can be separated from *P*. *vivatus* based on the following features: (1) posterolateral projection of male abdominal segment VIII subequal in length and width (Fig 18D and 18E); (2) male abdominal segment VIII 1.6–1.8 times longer than wide; and (3) left basolateral process of male proctiger with 3/5 of proctiger length. However, in *P*. *vivatus*: (1) the projection of the posterolateral margin of male abdominal segment VIII is 2.0–3.3 times longer than wide (Fig 27B); (2) the male abdominal segment VIII is about twice as long as wide; and (3) the left basolateral process of the male proctiger is subequal in length to the proctiger.

**Fig 39:**
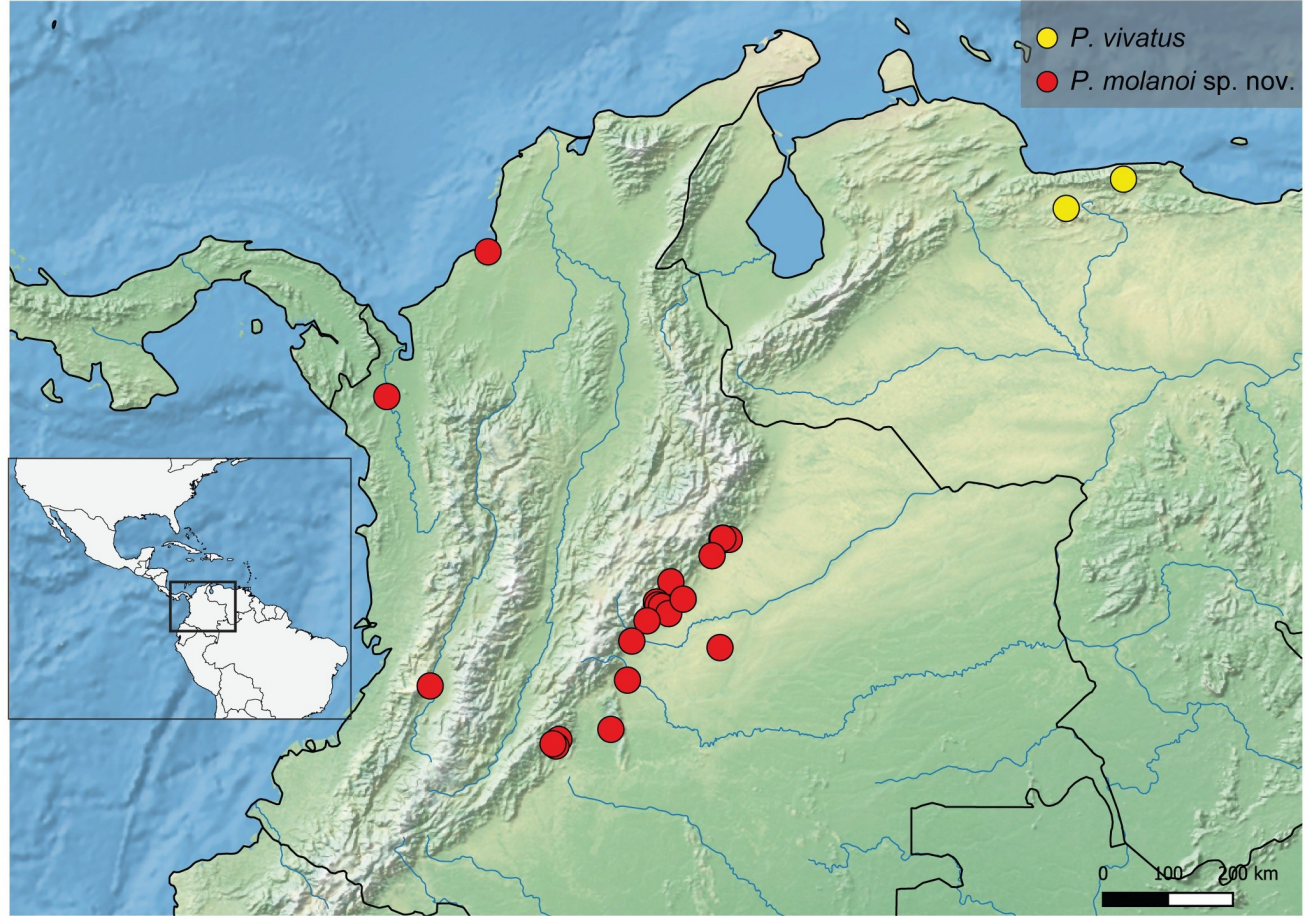
Map showing the geographical distribution of species of *P*. *vivatus*, *P*. *molanoi* sp. nov. Spatial data from Natural Earth (http://www.naturalearthdata.com/).

**Geographic distribution (Fig 39).** COLOMBIA: **Antioquia** [45], **Boyacá** [this work], **Caquetá** [38, this work], **Casanare** [1, 38 this work], **Chocó** [this work], **Cundinamarca** [36, 37, this work], **Meta** [1, 6, this work], **Sucre** [1], **Valle del Cauca** [45].

#### Type material examined

Holotype ♂ (UPTC-In-05081): ‘Colombia Meta\ Lugar V El Guape\ Caño Dormilon. F.\ Altura:\ Fecha: 12–01–04\ Col: C. Upegui’ ‘Gen: *Potamobates*\ Esp: *vivatus*\ Det: F. Molano’ ‘P. vi.0040’. Paratypes: 03♀, 3♂ (UPTC-In-05077): ‘‘Colombia Meta\ Lugar V El Guape\ Caño Dormilon. F.\ Altura:\ Fecha: 12–01–04\ Col: C. Upegui’ ‘Gen: *Potamobates*\ Esp: *vivatus*\ Det: F. Molano’ ‘P. vi.0040’. 02♀, 3♂, 1 nymph (UPTC-In-05077): ‘Ordem Hemiptera\ Subordem: Heteroptera\ Família: Gerridae\ Espécie: *Potamobates vivatus*\ Det: Galindo, X’ ‘Colombia San Luis de Gaceno Vda’ ‘La Granja. 4°50’27”N 73°10’29’W\ 463. Quebrada Jameo. 1.V. 2009\ Mondragón et al. # colecta 008’ ‘P. vi. 0035’. 5♀, 2♂ (UPTC-In-05082): ‘Colombia Meta\ Lugar V El Guape\ Caño Dormilon. F.\ Altura:\ Fecha: 12–01–04\ Col: C. Upegui’ ‘Gen: *Potamobates*\ Esp: *vivatus*\ Det: F. Molano’ ‘P. vi.004. 1♀, 1♂ (UPTC-in-05083): ‘Colombia Meta\ Lugar: S. de La Macarena\ Rio Santo Domingo\ Altura:\ Fecha 24/01/84\ Col: C. Movillas’ ‘*Potamobates*\ *vivatus*’ ‘P. vi. 0041’. 1♀, 4♂ (EQ): ‘P05\ 14.4.11\ *Potamobates*\ Q. Aracaleña (N)a Zcruce\ Hemiptera\ Gerridae 50 individuos’. 4♀, 1♂ (EQ): ‘#434\ Hemiptera\ Gerridae\ *Potamobates* sp2\ 5 individuos\ Q. La Balsa arriba R. Atrato\ R. sucio Chocó\ 16/12/96\ Ha proyecto: 0 spina’. 3♀, 3♂ (EQ): ‘Corriente Rio Pato\ Cuenca Rio Amazonas\ Subcuenca Rio Pato–Rio Caguán\ Altura 750 msnm\ Municipio S Vicente Del Caguán (Caq.)\ Colector Hernan Aristizabal G’ ‘ Muestra HA 342\ Hemiptera Heteroptera\ Gerridae\ *Potamobates* sp. 2\ Identifico HAG’. 1♀ (ICN): ‘AM-38\ Caño Carmelo\ Neuston\ Gerridae\ *Potamobates vivatus*’. 2♀, 1♂ (ICN): ‘Caño Tirriana\ AM-37 Neuston\ *Potamobates*\ *vivatus*’. 1♂ (ICN): ‘Hemiptera #453\ Gerridae\ *Potamobates vivatus*\ 1 individuo \ Q. los Laureles R. Meta\ Cusiana, Aguazul 30/11/00\ H. Aristizabal’. 2♀, 2♂ (ICN): P03 13-4-11\ Q. los vados aguas+\ HEMIPTERA\ Gerridae (4)\ *Potamobates*’. 7♀, 5♂ (ICN): ‘Corriente Caño Negro, Via\ Cuenca Rio Orinoco\ Subcuenca Rio Cusiana\ Altura 700 msnm\ Municipio Agua Azul (Casanare)\ Colector HERNAN ARISTIZABAL G.’ ‘Muestra HA 296\ Hemíptera Heteroptera\ Gerridae\ *Potamobates* sp. 2\ Identifico HAG’. 3♀, 1♂ (ICN): ‘P8 Q. La Candelilla\ Neuston\ Gerridae\ *Potamobates*\ *vivatus*’. 1♀, 1♂ (ICN): # 437\ Hemiptera\ Gerridae\ *Potamobates vivatus*\ Q. Medano, R. Meta Chartre Cusiana., Aguazul\ 30/11/00\ 3 individuos\ H. Aristizabal’. 6♂ (ICN): ‘Corriente Quebrada La Resaca\ Cuenca Rio Amazonas\ Altura 1000 msnm\ Municipio S. Vicente Del Caguán (Caq.)\ Colector HERNAN ARISTIZABAL G.’ ‘Muestra FA 340\ Hemiptera Heteroptera\ Gerridae\ *Potamobates* 2\ Identificó: HAG’. 1♀, 2♂ (ICN): ‘*Potamobates*\ *vivatus*\ P07\ Q. Honda\ Aguas arriba\ Circe\ 14-IV-II (3)’.

#### Additional material examined

1**♀**, 1**♂** (UPTC): ‘Colombia Caquetá Mun.\ San Vicente Del Caguan 1000 m\ Qda. la. Resaca 10.vii.1996\ Aristizábal, H.’ ‘*Potamobates*\ *vivatus*’. 1♀, 7♂, 18 nymphs (EQ): ‘Curriente Cortadera\ Cuenca Río Orinoco\ Subcuenca Rio Cravo Sur\ Altura 350 msnm\ Municipio El Yopal (casanare)\ Colector HERNAN ARISTIZABAL G’ ‘Muestra HÁ 309\ Hemiptera Heteroptera\ Gerridae\ *Potamobates* sp*\* Identificó: HAG’. 1♀ (EQ): ‘Q. Chaparral Neuston\ Hemiptera\ Gerridae\ *Potamobates*’. 12♀, 21♂, 27 nymphs (EQ): ‘P08\ 14/04/11 (59)\ Q. Aracaleña\ abajo ocacion (N)\ *Potamobates vivatus*’. 1♂ (INVERTUN): ‘Curriente Drenaje Caño Garagoa\ Cuenca Rio Orinoco\ Subcuenca Rio Cusiana\ Altura 800 msnm\ Municipio Agua Azul (Casanare)\ Colector Hernan Aristizabal G’ ‘Muestra HÁ 301\ Hemíptera Heteroptera\ Gerridae\ *Potamobates* sp. 2\ Identificó HAG’. 1♀, 1♂, 1 nymph (INVERTUN): ‘Corriente Quebrada Jaramá\ Cuenca Rio Orinoco\ Subcuenca Rio Cravo Sur\ Altura 350 msnm\ Municipio El Yopal (Casanare)\ Colector Hernan Aristizabal G’ ‘Muestra HA 309\ Hemiptera Heteroptera\ gerridae\ *Potamobates* sp. 2\ Idnetificó: HAG’. 2♀, 1♂ (INVERTUN): ‘Curriente Quebrada Jaramá\ Cuenca Rio Orinoco\ Subcuenca Rio Cravo Sur\ Altura 350 msnm\ Municipio El Yopal (Casanare)\ Colector Hernan Aristizabal G.’ ‘Muestra HA 303\ Hemiptera Heteroptera\ Gerridae\ *Potamobates* sp. 2\ Identificó HAG’. 2♀ (INVERTUN): ‘Cuerriente Quebrada Las Perlas\ Cuenca Rio Amazonas\ Subcuenca Rio Fato—Rio Gaguán\ Altura 1200 msnm\ Municipio S. Vicente Del Gaguán (Caq.)\ Colector Hernan Aristizabal G’ ‘Muestra HÁ 339\ Hemiptera Heteroptera\ gerridae\ *Potamobates* sp. 2\ Identifico: HAG’. 2♀, 4♂ (UPTC): ‘Colombia Cundinamarca\ Lugar: Granja. Ex\ Agropecuaria\ Altura 520 msnm\ Fecha 27/12/86\ Col: I Garcia Unal’ ‘P. vi. 0030’ ‘Gen: *Potamobates*\ Esp: *vivatus*\Det. F. Molano’. 1♀, 2♂ (UPTC): ‘Colombia Caqueta\ Mun. Sanvicente del\ Caguan 1060 m.\ Qba. La Resaca.\ 10\07\96\ Col: H. Aristizabal’ ‘*Potamobates*\ *vivatus*\ Det. F. Molano’ ‘P. vi. 0032’. 4♀ (UPTC): ‘Col. Meta. Pto López\ Corr. Altamira–Finca\\ Chaparralito 140m\ 9-I-09\ Col. Morales I.’ ‘P. Vi. 0033’ ‘*Potamobates*\ *vivatus*’. 1♂, 5 nymphs (UPTC): ‘Colombia Meta Granada\ Lugar: V. El Guape\ Caño Dormilon\ Altura:\ Fecha: 12/01/04\ Col: Upegui’ ‘P. vi. 0039’ ‘Gen: *Potamobates*\ Esp. *Vitatus*\ Det. F. Molano’. 1♀, 1♂, 1 nymph (UPTC): ‘Colombia Meta Acacías\ Lugar La Esmeralda\ Caño 7 vueltas\ Altura: 514 msnm\ Fecha: 24/04/04\ Col: Flores’ ‘Gen *Potamobates*\ Esp *vivatus*\ Det. F Molano’ ‘P. vi. 0038’. 2♀ (UPTC): ‘Colombia San Luiz de Gaceno Vda\ Argeles Farallones. 4°49’12”N\ 73°10’01”W 385. Quebrada. Jameo\ 2.VI.2009 Mondragón et al. # colecta\ 022’ ‘P. vi. 0037’ ‘Orden: Hemiptera\ Suborden: Heteroptera\ Familia: Gerridae\ Especie: *Potamobates vivatus*\ Det: Mondragón, P’. 1♀ e 1♂ (UPTC): ‘Colombia San Luis de Gaceno Vda\ Argeles Farallones. 4°49’12”N\ 73°10’01”W 385m. Charca. Jameo\ 2.vi.2009 Mondragón et al. # colecta\ 016’ ‘Orden: Hemiptera\ Subordem: Heteroptera\ Familia: Gerridae\ Especie: *Potamobates vivatus*\ Det. Hernandez, C.’ ‘P. vi. 0036’. 3♀, 2♂, 1 nymph (UPTC): ‘Colombia San Luiz de Gaceno Vda\ La granja. 4°49’46”N 73°10’20” W\ 414m. Quebrada. Jameo. 1.vi.2009\ Mondragón et al. #colecta 006’ ‘Ordem: Hemiptera\ Subordem: Heteroptera\ Familia Gerridae\ Especie *Potamobates vivatus*\ Det: Hernández, C.’ ‘P. vi. 0042’. 1♀, 3♂ (UPTC): ‘Colombia–Boyacá,\ San Luis de Gaceno\ V.da El Cairo 15-IV-10’ ‘P.vi.0004’ ‘*Potamobates*\ *vivatus*’. 1♀, 1♂ (UPTC): ‘Colombia, Boyocá\ San Luis Gaceno\ 14-04-10. JDA, El\ Cairo. Col. Rojas et al.’ ‘P. vi. 003’ ‘*Potamobates*\ *vivatus*’. 1♀, 1♂ (UPTC): ‘Col. Cudinamarca—Medina\ Quebrada 520 msnm\ 27–VII–86’ ‘P.V.0001’ *Potamobates vivatus*\ Det: Molano F.’. 2♀ e 5♂ (UPTC): ‘Colombia, Meta. Villavicencio\ Jardin Botánico 04°09’00”N\ 73°39’20”W 650 m.\ 29.III.2011 Col. M. Loaiza’ ‘*Potamobates*\ *vivatus*’ ‘P. vi. 0069’. 16♀, 15♂ (MUSENUV): ‘Colombia Meta\ Sra Macarena\ Altura\ Hosp Rio Sto Domingo\ Fecha I-24-89\ col C. Murilo’ ‘Replica\ Polhemus\ Nov– 1989’ ‘Fila Gerridae\ Subfila\ Gen:\ Sp\ Det: Polhemus’ ‘89–09’. 1♀ (MUSENUV): ‘Colombia–Meta\ Serraría La Macarena\ Paitas 600 m.s.m\ Água turbia color verdoso\ Zona abierta\ C. Murillo sept. 14 1989’ ‘Gerridae\ *Potamobates*\ *unidentatus*\ F. Molano’. 6♀, 7♂, 4 nymphs (MUSENUV): ‘Colombia–Meta\ Serraría La Macarena\ Bajo\ C. Murillo-Sept 14–1988’ ‘Gerridae\ *Potamobates*\ *unidentatus*\ F. Molano’ ‘F2411’ ‘Bajo’.

### *Potamobates osborni* Drake and Harris, 1928

(Figs 8 and 22)

*Potamobates osborni* Drake and Harris, 1928 [9]: 25, 26 (description). Drake and Harris (1934) [21]: 224–226, 238, 239 (figure, key, redescription). Kuitert (1942) [17]: 140, 141 (key, note). Polhemus and Polhemus (1995) [6]: 363, 364, 366–368, 372 (figure, key, phylogeny). Cognato (1998) [18]: 21, 22 (phylogeny). Buzzetti (2006) [19]: 55 (phylogeny). Padilla-Gil and Damgaard (2011) [7]: 44, 46 (key, phylogeny).

#### Diagnosis

Abdomen elongated, subequal in length to mesothorax; male pygophore and proctiger sinistrally rotated about 30° (Fig 8A, 8B, 8F and 8K); male abdominal segment VIII very long, about 2–3 times as long as wide, concave, with a median sulcus ventrally; female abdominal tergum VIII triangular, about 2.5 times longer than wide (Fig 22C); extension of female abdominal sternum VII short, oval, clearly shorter than mediotergite VII (Fig 8D).

#### Redescription

Length (**♂**: 11.9–12.3; ♀: 11.4–13.1); width (**♂**: 3.1–3.3; ♀: 3.3–3.7) (Fig 22). *Head*: antennomere I about 1.5 times as long as head, about three times as long as antennomere II; II about 1.3–1.6 times as long as III; IV subequal in length to III. Eye width 1.3–1.6 times interocular width. Article III of labium 1.5–1.9 times longer than IV. Antenna and dorsum of head black; vertex and base of antenniferous tubercle with yellowish mark; mandibular and maxillary plates yellowish; labium with articles I–II yellowish, III–IV black; venter of head yellowish. *Thorax*: Pronotum with yellowish median stripe, posterior margin not covered by golden setae; propleuron with anterior patch of golden setae; proacetabulum yellow, with a stripe of golden setae and a blackish triangular mark laterally; fore coxa and trochanter yellowish, trochanter with dorsum and apex blackish; fore femur yellow, with venter and apex black; fore tibia black with a brown mark laterally; fore tarsus black. Mesonotum without longitudinal stripes, posterior margin with golden setae; dorsal 3/4 of spiracle covered by golden setae; mesopleuron with longitudinal stripe of golden setae, stripe sigmoid, slightly curved, posteriorly discontinue; mesosternum with blackish mark anteriorly and lateral blackish marks on posterior third; limit between meso- and metasterna blackish; middle and hind coxae yellowish; middle and hind trochanters blackish with ventral apex brown; middle and hind femora with longitudinal black stripe dorsally and ventrally; middle and hind tibiae and tarsi yellowish to brownish. Metanotum without stripes; metacetabulum with stripe of golden setae laterally. *Abdomen*: medio- and laterotergites I–VII black; lateral margin of mediotergites with longitudinal stripe of golden setae; side of abdomen black, dorsally with longitudinal stripe of golden setae; sternum VII with brownish mark centrally on posterior margin. *Male*: posterior margin of abdominal mediotergite VII obstuse (Fig 8A). Posterior projection of last abdominal laterotergite (= connexival spine) with apex rounded (Fig 8A). Abdominal sterna II–VII with conspicuous median depression, depression shallower and smoother on sterna VI–VII (Fig 22B). Abdominal sternum VII with rounded notch on posterior margin, notch subequal in length and width. Abdominal segment VIII 2–3 times longer than wide, slightly flattened anteriorly, tubular posteriorly, concave (Fig 8K); lateral margins bowed centrally, converging anteriorly and posteriorly; posterodorsal margin obtuse; venter with weak median depression and unmodified lateral margins; posteroventral margin rounded, with inconspicuous rounded tooth on left side (Fig 8D). Pygophore and proctiger sinistrally rotated about 30° (Fig 8A, 8B, 8E and 8K). Pygophore longer than abdominal mediotergite VII. Proctiger longer than abdominal segment VII; anterior margin with short notch, notch with about 1/5 of proctiger length; right margin folded ventrally; left margin folded on basolateral process and part of proctiger; apex with many denticles; right basolateral process developed, triangular, much shorter than left basolateral process (Fig 8J); left basolateral process long, with about 1/3 of proctiger length and about 5 times length of right basolateral process, longitudinal axis diverging about 45° from longitudinal axis of proctiger, lateral margins parallel, apex truncate to rounded, not bifid (Fig 8J). Phallus short, smallest width about 3.6 times length (Fig 8G); dorsal sclerite slightly narrower and longer than ventral sclerite, with base not notched (Fig 8H); lateral sclerite triangular; ventral sclerite with base bifid, apex almost twice as wide as center and notched centrally (Fig 8I); transverse sclerite large, folded over ventral sclerite. Abdominal tergum VIII black; sternum VIII yellowish, posterior 1/4 with brownish mark; pygophore brownish; proctiger black, apex brownish. *Female*: abdominal tergum VIII triangular, about 2.5 times longer than wide; lateral margins strongly diverging posteriorly; posterior angle acute (Fig 8C and 8D). Abdominal laterotergites not folded over mediotergites (Fig 22C), without posterior projection (= connexival spine) (Fig 8C and 8D). Extension of abdominal sternum VII oval, about three times as wide as long, partially covering first pair of gonocoxae, not folded over tergum VIII (Fig 8D). Anal cone long, about as long as mediotergite VII. Dorsum of tergum VIII black, lateral margins brown. *Macropterous*: posterior margin of pronotum yellowish, remainder of body as in apterous form.

#### Variation

The color pattern is variable. On the paratypes, the dorsum of the head has a yellowish mark restricted to the vertex, while in other specimens only the frontoclypeus is black and the remainder of the head is yellowish. The median stripe of the pronotum is narrow on the paratypes, narrower than the interocular width, while in other specimens it is subequal to the interocular width. The male abdominal sternum VIII usually has denticles, but these can be absent. On the paratypes, the lateral margins of the left basolateral process of the proctiger slightly diverge distally, while in other males the margins are parallel.

#### Comments

The male of *P*. *osborni* has the pygophore and proctiger sinistrally rotated by no more than 45° (Fig 8A, 8B, 8E and 8K), similar to *P*. *bidentatus*, *P*. *horvathi*, *P*. *manzanoae*, and *P*. *unidentatus*. It can be distinguished from these species by the elongated male abdominal segment VIII (Fig 8A and 8B), which is ventrally concave and with a median sulcus (Fig 8B, 8E and 8K).

**Geographic distribution (Fig 37).** MEXICO: **Chiapas** [this work], **Veracruz** [9, this work].

#### Type material examined

1♂ paratype (USNM): ‘Motzorongo V. C.\ Mex. Feb.//’92’ ‘H. Osborn\ Collector’ ‘H.E.Summers\ Collection’ ‘PARATYPE\ *Potamobates*\ *osborni*.\ Drake & Harris’ ‘C.J. Drake\ Coll. 1965’. 1♀ paratype (USNM): ‘Motzorongo V. C.\ Mex. Feb.//’92’ ‘PARATYPE\ *Potamobates*\ *osborni*.\ Drake & Harris’ ‘C.J. Drake\ Coll. 1965’.

#### Additional material examined

1♂ (LACM): ‘MEX: Chiapas\ Palenque\ 24 Jan. 1973\ R. C. A. RICE’ ‘*Potamobates*\ *osborni* (D & H)\ det. Calabrese 1975’ ‘LACM ENT 315713’. 2♀, 5♂ (LACM): ‘MEX: Chiapas\ Palenque\ 24 Jan. 1973\ R. C. A. RICE’. 1♂, 1♀ (USNM): ‘Orizaba\ Mexico\ Jan ‘92’. 1♀, 1♂ (USNM): ‘MEX., Chiapas\ 7 Mi N of Santa Fe\ CL1102 5 May1964\ J.T. & M.S. Polhemus’ ‘Homeotype\ *Potamobates*\ *osborni* D&H\ Compared with type\ by J.T. Polhemus’ ‘J.T. Polhemus\ Collection 2014\ C.J.Drake Accession’. 1♀, 1♂ (USNM): ‘MEX., Chiapas\ 7 Mi N of Santa Fe\ CL1102 5 May1964\ J.T. & M.S. Polhemus’ ‘*Potamobates*\ *osborni*\ Det. J.T. Polhemus’ ‘J.T. Polhemus\ Collection 2014\ C.J.Drake Accession’. 3♀, 2♂ (USNM): ‘MEX., Chiapas\ 7 Mi N of Santa Fe\ CL1102 5 May1964\ J.T. & M.S. Polhemus’ ‘J.T. Polhemus\ Collection 2014\ C.J.Drake Accession’. 5♀, 9♂, 3 nymphs (USNM): ‘S. of Loma Bonita\ MEXICO, CL 1333\ Ver, I-15, 1970\ J.T.Polhemus’ ‘J.T. Polhemus\ Collection 2014\ C.J.Drake Accession’.

### *Potamobates peruvianus* Hungerford, 1936

(Figs 12 and 30)

*Potamobates peruvianus* Hungerford, 1936 [4]: 178–180 (description). Hungerford (1937b) [14]: 146, 147 (figure). Kuitert (1942) [17]: 140, 141 (key, note). Matsuda (1960) [2]: 210, 211 (figure). Polhemus and Polhemus (1995) [6]: 365, 367–369, 372 (key, phylogeny). Cognato (1998) [18]: 21, 22 (phylogeny). Padilla-Gil and Damgaard (2011) [7]: 45, 46 (key, phylogeny). Morales et al. (2013) [5]: 194 (figure).

#### Diagnosis

Male pygophore and proctiger sinistrally rotated about 90° (Fig 12A and 12D); right posterolateral margin of male abdominal segment VIII with a single projection (projection about three times as long as wide) (Fig 12D); female abdominal tergum VIII subequal in length to mediotergite VII (Fig 12B); extension of female abdominal sternum VII short, half as long as mediotergite VII, with a lobule, not folded over tergum VIII (Fig 12C).

#### Redescription

Length (**♂**: 9.3–11.0; ♀: 8.5–9.2); width (**♂**: 2.1–2.6; ♀: 2.5–2.8) (Fig 30). *Head*: antennomere I 1.0–1.2 times longer than head width, 2.6–2.9 times longer than antennomere II; II 1.0–1.2 times longer than III; IV 1.2–1.6 times longer than III. Eye width 1.4–2.0 times interocular width. Article III of labium 1.8–2.3 times longer than article IV. Antenna black, base of antennomere IV dark brown; dorsum of head yellowish; frons with blackish mark; antenniferous tubercle slightly darker than remainder of head dorsum; mandibular and maxillary plates yellowish; labium with articles I–II and basal 1/3 of III yellowish; apex of III and whole IV blackish; venter of head yellowish. *Thorax*: Pronotum with wedge-shaped light-brown mark, posterolateral angles covered by golden setae; propleuron with anterior patch of golden setae; proacetabulum with stripe of golden setae laterally, posterior margin blackish; fore coxa and trochanter yellowish, trochanter with brownish median mark ventrally; fore femur yellowish, venter and apex blackish; tibia and tarsus blackish. Mesonotum usually without longitudinal stripes, posterior margin with golden setae; dorsal 3/4 of spiracle covered by golden setae; mesopleuron with longitudinal stripe of golden setae, stripe sigmoid, slightly curved, posteriorly discontinue; mesosternum without marks; middle and hind coxae yellow; middle and hind trochanters brown, venter with brownish mark; middle and hind femora yellowish, with brownish to black stripe dorsally and ventrally; middle and hind tibiae and tarsi yellowish to brownish. Metanotum without stripes, posterior margin with golden setae; metacetabulum with stripe of golden setae laterally. *Abdomen*: medio- and laterotergites I–VII black, lateral margins of mediotergites with longitudinal stripe of golden setae; side of abdomen black, dorsally with longitudinal stripe of golden setae. *Male*: posterior margin of abdominal mediotergite VII rounded. Posterior projection of last abdominal laterotergite (= connexival spine) triangular (Fig 12A). Abdominal sterna II–VII without median depression. Sternum VII with rounded notch on posterior margin, notch about as long as wide. Abdominal segment VIII 1.4–1.6 times as long as wide, tubular, lateral margins slightly divergent posteriorly, anterior width about 9/10 of posterior width; right side with a narrow, long projection (length about 3 times basal width), projection with acute apex, directed posteroventrally (Fig 12D); segment slightly bulged ventrally to projection; venter with median depression on anterior half or whole segment, posteroventral margin with an indentation and with central tooth. Pygophore and proctiger sinistrally rotated about 90° (Fig 12A and 12D). Pygophore longer than abdominal mediotergite VII. Proctiger length 9/10 of abdominal segment VII length; anterior margin with a long notch, notch with 1/3 of proctiger length; right margin folded ventrally; left margin strongly folded on left basolateral process up to about the middle of proctiger; apex without denticles; right basolateral process developed, rounded, with less than 1/5 of left basolateral process length; left basolateral process long (about 1.3 times as long as wide, length about 7/10 of proctiger length), longitudinal axis diverging about 100° of longitudinal axis of proctiger, lateral margins converging distally, apex angled, not bifid. Phallus elongated (Fig 12E); dorsal sclerite anteriorly coiled, slightly narrower and much longer than ventral sclerite, base not notched (Fig 12E); lateral sclerite triangular; base of ventral sclerite bifid, apex slightly wider than base (Fig 12F); transversal sclerite not visible (Fig 12E). Abdominal tergum VIII black, with yellowish lateral margins, venter with blackish marks on posterolateral angles. Pygophore yellow, with brownish or blackish mark on posterior half of dorsum and venter. Proctiger blackish or brownish, with apex yellow. *Female*: abdominal tergum VIII triangular, lateral margins strongly divergent posteriorly, length 7/10 to subequal to width, posterior margin rounded (Fig 12B); anal cone short. Abdominal laterotergites not folded over mediotergites, except on last segment (Fig 30C); posterior projection of last abdominal laterotergite (= connexival spine) tubular, longer than abdominal tergum VII, apex rounded (Fig 12B). Extension abdominal of sternum VII oval, about twice as wide as long, with a lobule, completely covering first pair of gonocoxae, not folded over tergum VIII (Fig 12C). Abdominal tergum VIII black with lateral margins yellow. *Macropterous*: pronotum with central mark anteriorly, posterolateral 2/3 and posterior margin yellowish.

#### Variation

The dorsum of the head can be uniformly yellowish or display a large mark from the anterior margin to the middle of the eyes. The antenna can be brownish and, in some specimens, with the venter and distal 1/3 of antennomere I yellowish. From 1/3 to 2/3 of article III of labium can be yellowish. The proacetabulum can have a brownish mark laterally. The fore femur can have the mesal margin and the distal 2/3 blackish. The mesonotum can have a longitudinal stripe on the posterior half and/or a mark on posterolateral 2/3. The female abdominal segment VIII can be slightly elongated and curved as in *P*. *tridentatus* and *P*. *anchicaya*.

#### Comments

The male of *P*. *peruvianus* has the pygpophore and proctiger sinistrally rotated about 90° (Fig 12A and 12D), similar to *P*. *anchicaya*, *P*. *bilobulatus*, *P*. *carvalhoi*, *P*. *molanoi* Floriano and Moreira, **sp. nov.**, *P*. *shuar*, *P*. *spiculus*, *P*. *sumaco*, *P*. *tridentatus*, *P*. *variabilis*, *P*. *vivatus*, *P*. *williamsi*, and *P*. *woytkowskii*. It differs from most of these species by the long projection (three times as long as wide) on the right margin of abdominal segment VIII (Fig 12D), which is also seen in *P*. *shuar* and *P*. *variabilis* (Fig 11D). Males of *P*. *peruvianus* can be further distinguished from *P*. *variabilis* by the left basolateral process of the proctiger with the apex not bifid and by the always single projection on the right posterolateral margin of abdominal segment VIII (Fig 12D), whereas in *P*. *variabilis* the left basolateral process of the proctiger has the apex bifid (Fig 11A and 11H) and the right posterolateral margin of abdominal segment VIII can bear a single projection or a pair (Fig 11B–11D). The males of *P*. *shuar* and *P*. *peruvianus* are very similar and share a single posterolateral projection on abdominal segment VIII. However, *P*. *peruvianus* displays (1) a slight bulging ventrally to the posterolateral projection of abdominal segment VIII (Fig 11D); (2) the left basolateral process of the proctiger about 1.3 times as long as wide; and (3) the posterior region of the left basolateral process of the proctiger with the margins converging distally. On the other hand, *P*. *shuar* displays (1) no bulging ventrally to the posterolateral projection of abdominal segment VIII; (2) the left basolateral process of the proctiger about 1.5 times longer than the proctiger; and (3) the posterior region of the left basolateral process of the proctiger with the margins diverging distally.

**Fig 40:**
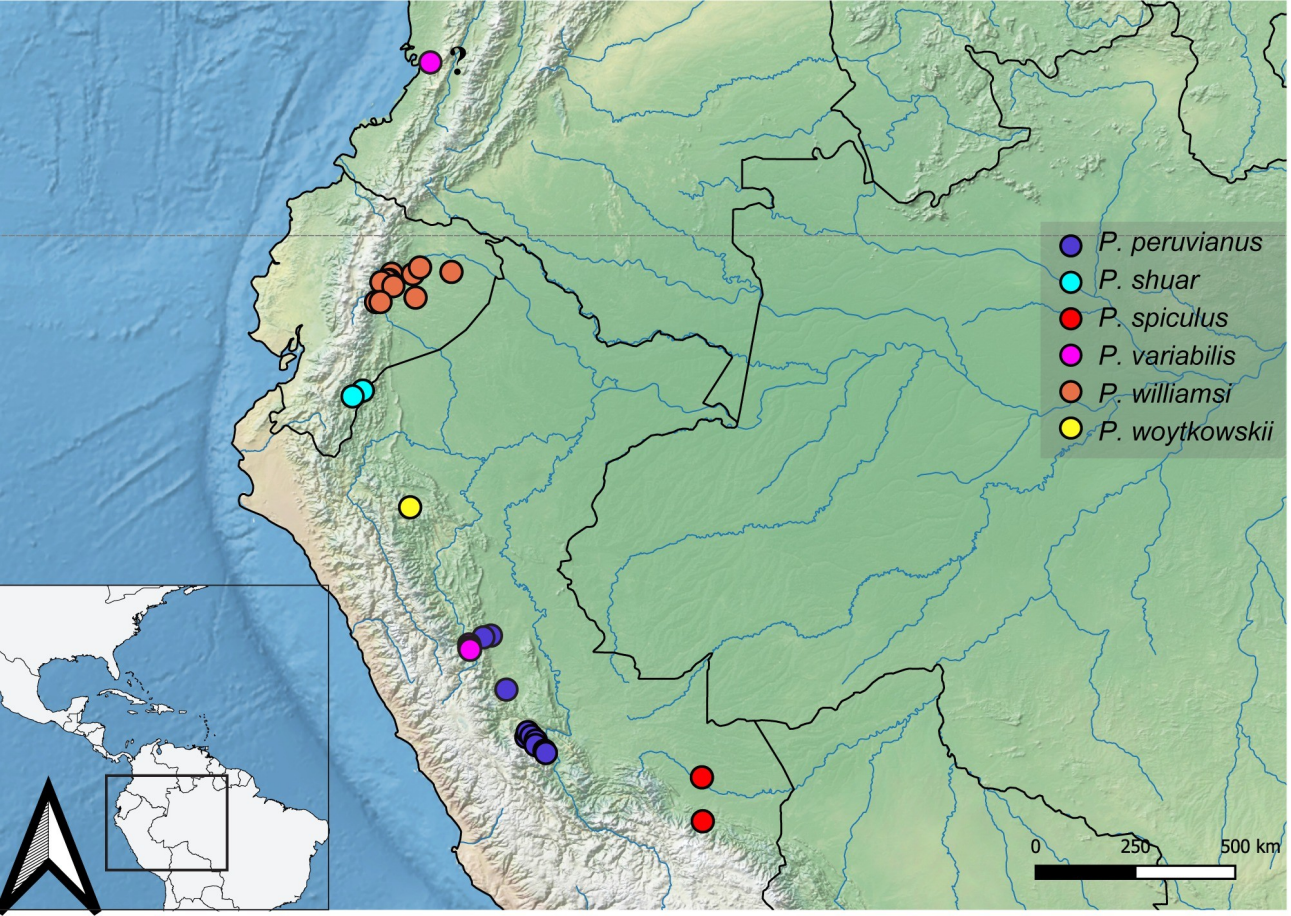
Map showing the geographical distribution of species of *P*. *peruvianus*, *P*. *shuar*, *P*. *spiculus*, *P*. *variabilis*, *P*. *williamsi*, *P*. *wotkowskii*. Spatial data from Natural Earth (http://www.naturalearthdata.com/).

**Geographic distribution (Fig 40).** PERU: **Junin** [4, 17, 54, this work], **Pasco** [this work], **Ucayali** [this work].

#### Type material examined

2♀, 4♂ paratypes (USNM): ‘PARATYPE\ *Potamobates\ peruvianus*\ H. B. Hungerford’ ‘Vic. Of San Pedro\ 900 m. a. s. l.\ jungle Pools’ ‘Peru, S. A.\ May 15–29, 1935\ F. Woytkowski’ ‘Paratype No\ 52009\ U.S.N.M’.

#### Additional material examined

2♀, 3♂ (LACM): ‘Chontilla, 22km.\ SE Iscozazin,\ Dept. Pasco, PERU\ July 9, 1961\ F.S. Truxal’. 2♀ (USNM): ‘Satipo, Peru\ July–Aug 1940\ Pedro Paprzycki’ ‘J C Lutz\ Collection\ 1961’. 1♂: (USNM): ‘Satipo, Peru\ July–Aug 1940\ Pedro Paprzycki’. 10♂, 3♀ (USNM): ‘Aguaitia Dept\ de Loreto\ IX 19. 46\ F. Woytkowskii’ ‘J. T. Polhemus\ Collection 2014\ C. J. Drake Accession’. 7♂, 4♀ (USNM): ‘Santa Elena\ Boqueron Padre\ Abad Dept.\ Loreto Peru’ ‘VIII 46\ F. Woytkowskii’ ‘J. T. Polhemus\ Collection 2014\ C. J. Drake Accession’. 1♀, 1♂ (USNM): ‘Chontilha, 22km.\ SE Iscozazin,\ Dept. Pasco, Peru\ July 9, 1961\ F.S. Truxal’ ‘*Potamobates*\ *peruvianus*\ Hung.’ ‘J. T. Polhemus\ Collection 2014\ C. J. Drake Accession’. 1♀ (USNM): ‘Satipo, Peru\ July–Aug 1940\ Pedro Paprzycki’ ‘*Potamobates*\ *peruvianus*\ 63 Hung.\ Det. J. T. Polhemus’ ‘J. T. Polhemus\ Collection 2014\ C. J. Drake Accession’. 2♀ (USNM): ‘Peru. S. A.\ Satipo Nov 1942\ Pedro Paprzycki’ ‘J. T. Polhemus\ Collection 2014\ C. J. Drake Accession’. 38♀, 94♂ (USNM): ‘Satipo, Peru\ July–Aug. 1940\ Pedro Paprzycki’ ‘C J Drake\ Coll. 1956’. 4♀, 4♂ (USNM): ‘Satipo Peru\ July–Aug 1940\ Pedro Paprzycki’ ‘J C Lutz\ Collection\ 1961’. 13♀, 13♂ (AMNH): ‘Peru: Junin: between\ San Ramon de Pangoa\ and Boca de Kiatari,\ 40–55 km SE Satipo,\ 750 m., July 27, 1972\ R.T. & J.C. Schuh’. 1♂ (AMNH): ‘Peru: Junin: San Emiliano\ sw Cachingareni,\ approx. 55 km SE Satipo\ 1000 m., May 20–21, 1972\ R.T. & J.C. Schuh’. 1♀, 1♂ (AMNH): ‘Peru: Junin: San Ramón\ de Pangoa, 40 km S\ Satipo, 750 meters,\ January 28, 1974\ R.T. Schuh’. 11♀, 12♂ (AMNH): ‘Peru: Junin Dept; 105–110 km, SE of Satipo, 800m. betw. Bajo&Alto\ Tsitsireni, August 3, 1972, RT. & J.C. Schuh’.

### *Potamobates shuar* Buzzetti, 2006

(Figs 25D, 25I and 26)

*Potamobates shuar* Buzzetti, 2006 [19]: 55 (description, figure, phylogeny). Padilla-Gil and Damgaard (2011) [7]: 45, 46 (key, phylogeny). Morales et al. 2013 [5]: 194 (figure).

#### Diagnosis

Male pygophore and proctiger sinistrally rotated about 90° (Figs 25D, 25I and 26B); right posterolateral margin of male abdominal segment VIII with a single or a pair of projections (Fig 25D); posterior margin of male abdominal sternum VIII with weak notch (Fig 25D); left basolateral process of male proctiger not bifid, lateral margins diverging posteriorly (Fig 25D); posterior projection of last female abdominal laterotergite (= connexival spine) about twice as long as mediotergite VII (Fig 26C and 26D); female abdominal tergum VIII subequal in length and width; posterior margin of extension of female abdominal sternum VII with a lobule, lobule about half as long as tergum VII.

#### Comments

**Fig 41:**
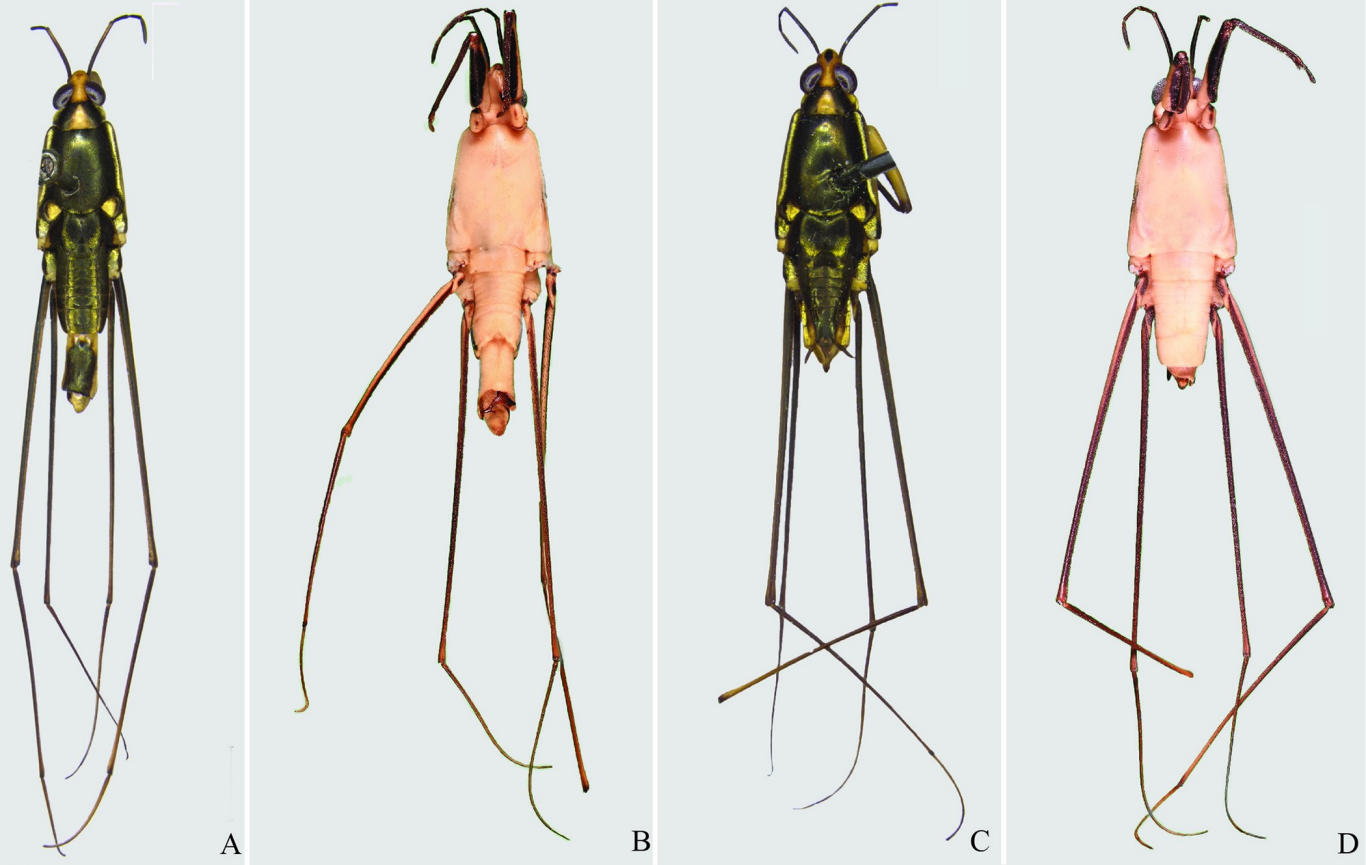
*Potamobates variabilis*. (A) Male, dorsal view; (B) male, ventral view; (C) female, dorsal view; (D) female, ventral view.

The male of *P*. *shuar* has the pygophore and proctiger sinistrally rotated about 90°, similar to *P*. *anchicaya*, *P*. *bilobulatus*, *P*. *carvalhoi*, *P*. *molanoi* Floriano and Moreira, **sp. nov.**, *P*. *peruvianus*, *P*. *spiculus*, *P*. *sumaco*, *P*. *tridentatus*, *P*. *variabilis*, *P*. *vivatus*, *P*. *williamsi*, and *P*. *woytkowskii*. It differs from most of these species, except for *P*. *variabilis*, by possessing a single or a pair of projections on the right posterolateral margin of male abdominal segment VIII. The two can be distinguished by the non-bifid left basolateral process of male proctiger and the more pronounced notch on the posterior margin of male abdominal sternum VIII in *P*. *shuar*, whereas in *P*. *variabilis* the apex of the left basolateral process of male proctiger is bifid (Fig 11A and 11H) and the notch on the posterior margin of male abdominal sternum VIII is inconspicuous (Fig 11A). The females of these species differ by the long posterior projection of the last abdominal laterotergite (= connexival spine) in *P*. *shuar* (about twice as long as mediotergite VII) (Fig 26C), which is shorter in *P*. *variabilis* (subequal to mediotergite VII) (Fig 41C). When describing *P*. *shuar*, Buzzetti (2006) [19] reported a single projection on the right posterolateral margin of male abdominal segment VIII. However, while studying material collected in Gualaquiza, Ecuador, about 10 km from the type-locality, Bomboiza, we noticed the presence of a single or a pair of projections.

**Geographic distribution (Fig 40).** ECUADOR: **Morona Santiago** [19, 55, this work].

#### Material examined

7♀, 7**♂** (USNM): ‘Ecuador, Morona-Santiago\ Gualaquiza, (17 km SW)\ 03°38’S 78°38’W\ 20 Sep 1990,\ colln #29\ P.J. Spangler & K. Bastidas’.

### *Potamobates spiculus* Polhemus and Polhemus 1983

**(Figs** 20, 25E, 25J, 25K and 25N**)**

*Potamobates spiculus* Polhemus and Polhemus, 1983 (description) [16]. Polhemus and Polhemus (1995) [6]: 365, 367–369, 372 (key, phylogeny). Cognato (1998) [18]: 21, 22 (phylogeny). Buzzetti (2006) [19]: 55 (phylogeny). Padilla-Gil and Damgaard (2011) [7]: 45, 46 (key, phylogeny).

#### Diagnosis

Male pygophore and proctiger sinistrally rotated about 90° (Fig 25E); right posterolateral margin of male abdominal segment VIII with a pair of ventral projections, dorsalmost projection long and sharp (about twice as long as wide), ventralmost projection oval to triangular (subequal in length and width) (Fig 25J and 25K) posteroventral margin of male abdominal segment VIII without large notch, with a triangular tooth centrally (Fig 25E); posterior projection of last female abdominal laterotergite (= connexival spine) long, subequal in length to mediotergite VII (Figs 20C and 25N); female abdominal tergum VIII about three times as long as wide, lateral margins strongly divergent posteriorly on anterior 1/3 (Figs 20C and 25N); extension of female abdominal sternum VII with a long lobule, lobule about three times longer than the smallest length of sternum VII and slightly twisted (Fig 20D).

#### Comments

The male of *P*. *spiculus* has the pygophore and proctiger sinistrally rotated about 90°, similar to *P*. *anchicaya*, *P*. *bilobulatus*, *P*. *carvalhoi*, *P*. *molanoi* Floriano and Moreira, **sp. nov.**, *P*. *peruvianus*, *P*. *shuar*, *P*. *sumaco*, *P*. *tridentatus*, *P*. *variabilis*, *P*. *vivatus*, *P*. *williamsi*, and *P*. *woytkowskii*. It can be distinguished from all these species by the following combination of characters: right posterolateral margin of male abdominal segment VIII with a pair of ventral projections, of which the dorsalmost projection is long and sharp, and the ventralmost projection is shorter and oval to triangular; and extension of female abdominal of sternum VII with a very long lobule, about three times longer than the smallest length of sternum VII. The latter character is also observed in females of *P*. *woytkowskii*. For further comparison between these species, see the comments under *P*. *woytkowskii*.

**Geographic distribution (Fig 40).** PERU: **Cusco** [16, 56, this work].

#### Type material examined

2♀, 3♂ paratypes (USNM): ‘Quincemil, Peru\ IX–1962\ Luis E. Pena’ ‘J. P. Polhemus\ Collection’ ‘Paratype\ *Potamobates*\ *spiculus*\ J.T. & D.A Polhemus’.

### *Potamobates sumaco* Cognato, 1998

(Figs 9 and 31)

*Potamobates sumaco* Cognato, 1998 [18]: 17–21 (description, figure, key, phylogeny). Buzzetti (2006) [19]: 55 (phylogeny). Padilla-Gil and Damgaard (2011) [7]: 45, 46 (key, phylogeny). Morales et al. (2013) [5]: 194 (figure).

#### Diagnosis

Male pygophore and proctiger sinistrally rotated about 90° (Fig 9A and 9B); male abdominal segment VIII with a pair of projections on the right posterolateral margin, projections 2–4 times as long as wide, inserted separately from each other (Fig 9B–9E); left basolateral process of male proctiger abruptly twisted by 90° at the base (Fig 9A); extension of female abdominal sternum VII folded over tergum VIII; posterior portion of macropterous pronotum yellowish.

#### Variation

The projections on the right posterolateral margin of male abdominal segment VIII are very variable, especially regarding their length. In some specimens, the ventralmost projection is very reduced and only a small protuberance is visible (Fig 9D).

#### Comments

The male of *P*. *sumaco* has the pygophore and proctiger sinistrally rotated about 90° (Fig 9A and 9B), similar to *P*. *anchicaya*, *P*. *bilobulatus*, *P*. *carvalhoi*, *P*. *molanoi* Floriano and Moreira, **sp. nov.**, *P*. *peruvianus*, *P*. *shuar*, *P*. *spiculus*, *P*. *tridentatus*, *P*. *variabilis*, *P*. *vivatus*, *P*. *williamsi*, and *P*. *woytkowskii*. It can be distinguished from most of these species, except for *P*. *anchicaya*, *P*. *tridentatus*, and *P*. *williamsi*, by the pair of long projections on the right posterolateral margin of male abdominal segment VIII (Fig 9B). Both *P*. *anchicaya* and *P*. *tridentatus* differ from *P*. *sumaco* because their projections are inserted united by the base (Fig 1F and 1G), whereas in the last species they are separated by a distance subequal to their length (Fig 9B). The male of *P*. *sumaco* can be distinguished from *P*. *williamsi* by the left basolateral process of the proctiger abruptly twisted by 90° at the base (Fig 9A), whereas in the latter species the twist occurs throughout the length of the process (Fig 28B). As for the females, they have the extension abdominal of sternum VII folded over the tergum VIII in *P*. *williamsi*, which does not happen in *P*. *sumaco*.

**Geographic distribution (Fig 36).** COLOMBIA: **Cauca** [38, this work], **Caquetá** [30, this work], **Putumayo** [38, this work]. ECUADOR: **Orellana** [18, 57]; **Napo** [18, this work], **Sucumbios** [18, 38, 57, this work]. Considering the known distribution of this species, the record from Casanare Department, northeastern Colombia [1], might be the result of mislabeling or misidentification.

#### Type material examined

7♀, 7♂ paratypes (USNM): ‘Ecuador: Napo Prov.\ stream near community of\ Guacamayo near Mondana\ 26-II-1997. K. Galacatos Coll’ ‘PARATYPE\ *Potamobates sumaco*\ Cognato’.

#### Additional material examined

4♀, 2♂ (EQ): ‘Gerridae # 223 HA\ *Potamobates* cf. *williamsi*\ *Q*. *Tufán/* R. Caquetá/ R. Amazonas\ Municipio Santa Rosa–Cauca\ HA 223 8–05–92\ CH’. 4♂, 6 nymphs (EQ): ‘Corriente Rio Playayacu escuela\ Cuenca Rio Amazonas\ Subcuenca Rio Napo\ Altura 250 msnm\ Municipio Francisco Orellana (Ecuador)\ Colector Hernan Aristizabal G’ ‘Muestra HA 243\ Hemiptera Heteroptera\ Gerridae\ *Potamobates williamsi*\ Identificó: HAG’. 8♀, 9♂, 2 nymphs (EQ): Corriente Rio Playayacu arriba\ Cuenca Rio Amazonas\ Subcuenca Rio Napo\ Altura 250 msnm\ Francisco Orellana (Ecuador)\ Colector Hernan Aristizabal G’ ‘Muestra HA 242\ Hemiptera Heteroptera\ Gerridae\ *Potamobates williamsi*\ Identificó: HAG’. 1♂ (INVERTUN): ‘Curriente Charca\ Cuenca Rio Amazonas\ Subcuenca Rio Caquetá\ Altura 700 msnm\ Municipio Mocoa (Putamayo)\ Colector HERNAN ARISTIZABAL’ ‘Muestra HA 168\ Hemipera Heteroptera\ Gerridae\ *Potamobates williamsi*\ Identifico: HAG’. 1♀ (UPTC): ‘Colombia-Caqueta, Floren\ Lugar: Q. La sardina\ Altura:\ Fecha:30/03/04\ Col: C. Serrato’ ‘Gen: *Potamobates*\ Esp: *sumaco*\ det: F. Molano’ ‘P. su. 0047’. 4♂ (UPTC): ‘Colombia Caqueta\ Lugar: Florencia\ Q. el dedo\ Altura: 450msnm\ Fecha: 01/03/03\ Col: C. Serrato’ ‘P. su. 0046’ ‘Gen: *Potamobates*\ Esp: *sumaco*\ Det: D. Camacho’. 1♀ (UPTC): ‘Colombia-Caqueta\ Lugar Q. La Perdiz\ V. Agua Negra\ Altura:\ Fecha: 13/11/03\ Col: C. Serrato’ ‘P. su. 0045’ ‘Gen: *Potamobates*\ Esp: *sumaco*\ Det: F. Molano’. 1♀, 2♂ (UPTC): ‘Colombia Caqueta.Flore\ Lugar: Q. La Vuca\ Altura:\ Fecha: 12/12/03\ Col. C. Serrato’ ‘P. su. 0044’ ‘Gen: *Potamobates*\ *sumaco*\ Det. D. camacho’. 5♀, 3♂ (UPTC): ‘Colombia-Caqueta\ Lugar: Florencia\ Q. Machilerito\ Altura: 10/07/03\ Col: C. Serrato’ ‘Gen: *Potamobates*\ Esp: *sumaco*\ Det: D. Camacho’ ‘P. su. 0043’. 3♀, 2♂, 2 nymphs (UPTC): ‘Col. Caqueta\ Los Estrechos\ Col: C. Serrato’ ‘*Potamobates*\ *sumaco*\ Det. D. Camacho’ ‘P. su. 0048’. 4♂ (UPTC): ‘Colombia-Caqueta\ Mun. Paujil\ Altura:\ Fecha: 17enero/2004\ Coleta Qda Banego\ Colector C. Serrato’ ‘P. su. 0049’ ‘F. Gerridae\ G. *Potamobates*\ Sp. *Sumaco*\ Det. F. molano’. 3♀, 1♂ (UPTC): ‘Colombia-Caqueta\ Lugar: Florencia\ Nueva Jerusalem\ Altura:\ Fecha: 16/06/02\ Col: C. Serrato’ ‘Gen: *Potamobates*\ Esp: *sumaco*\ Det: F. Molano’ ‘P. su. 0050’. 1♀, 2♂, 2 nymphs (UPTC): ‘Colombia-Caqueta-Flore\ Lugar: V. La. Florida\ Q. La. Sardina\ Altura:\ Fecha: 30/03/04\ Col: C. Serrata’ ‘P. su. 0051’ ‘Gen: *Potamobates*\ Esp: *sumaco*\ Det: D. Camacho’. 1♂ (UPTC): ‘Colombia-Caqueta\ Lugar: Florencia\ Q. La Sardina\ fecha: 15/02/04\ Col: C. Serrato’ ‘P. su. 0052’ ‘Gen: *Potamobates*\ Esp: *sumaco*\ Det: D. Camacho’. 5♀, 4♂ (UPTC): ‘Colombia Caqueta\ Qba. Yumal\ Altura:\ Fecha: 29 Agost/05\ Colecta:\ Colector: C. Serrato’ ‘F. Gerridae\ G. *Potamobates*\ Sp. *Sumaco*\ Det F. molano’ ‘P. su. 0053’. 1♂, 1♀ (USNM): ‘Ecuador: Napo Prov.\ stream nr. Community of Guacamayo. Nr. Mondaña\ I–1998, K. Galacatos’ ‘*Potamobates*\ *sumaco* Cognato\ Det. A. I. Cognato. 1998’ ‘J. T. Polhemus\ Collection 2014\ C. J. Drake Accession’.

### *Potamobates tridentatus* Esaki, 1926

**(Figs 25F,** 25L, 25P and 32**)**

*Potamobates tridentatus* Esaki, 1926 [10]: 251–254 (description, figure). Drake and Harris (1934): 225, 240, 241 (figure, key, redescription). Kuitert (1942) [17]: 141 (note). Cognato (1998) [18]: 21, 22 (phylogeny). Buzzetti (2006) [19]: 52, 53, 55 (phylogeny). Polhemus and Polhemus (1995) [6]: 365, 367, 368, 372 (key, phylogeny). Padilla-Gil and Damgaard (2011) [7]: 45, 46 (key, phylogeny). Morales et al. (2013) [5]: 194 (figure).

#### Diagnosis

Male pygophore and proctiger sinistrally rotated about 90° (Fig 25F and 25L); right posterolateral angle of male abdominal segment VIII with a pair of projections, projections inserted together by the base, about three times as long as wide (Fig 25L); posteroventral margin rounded, forming a large, sinistrally directed, rounded notch (Fig 25F); left basolateral process of male proctiger not reaching left margin of abdominal segment VIII (Fig 25P).

#### Redescription

Length (**♂**: 9.3–9.6; ♀: 8.1–9.2); width (**♂**: 2.5–2.7; ♀: 2.6–2.9) (Fig 32). *Head*: antennomere I 1.1 times longer than head width, 3.1 times longer than antennomere II; II with 0.9–1.1 times the length of III; IV 1.4–1.7 times longer than III. Eye width 1.7 times larger than interocular distance. Article III of labium 1.5 times longer than article IV. Antenna black, with the base of antennomere IV sometimes yellowish; dorsum of head yellow, frons with a blackish mark; antenniferous tubercle yellowish to blackish; mandibular and maxillary plates yellowish; labium with articles I–II and base of III yellow, remainder of III brownish to blackish, IV blackish; venter of head yellowish. *Thorax*: Pronotum with a central wedge-shaped yellowish mark, posterior margin without golden setae; propleuron with an anterior patch of golden setae; proacetabulum yellow, with a lateral stripe of golden setae and a blackish mark on posterior margin; fore coxa and trochanter yellowish, trochanter with a blackish stripe ventrally; fore femur yellowish, venter and apex blackish; fore tibia and tarsus black. Mesonotum uniformly black or with a yellowish median stripe, posterior margin covered by golden setae; dorsal 3/4 of spiracle covered by golden setae; mesopleuron with a longitudinal stripe of golden setae, stripe sigmoid, curved, posteriorly discontinue; mesosternum without marks; mesoacetabulum with a lateral stripe of golden setae; middle and hind coxae and trochanters yellowish, trochanters with a central brownish mark basally; middle femur and tibia with a blackish longitudinal stripe on dorsum and venter, apical 3/4 of hind femur blackish; remainder of hind femur, hind tibia, and middle and hind tarsi brownish, lighter toward apex. Metanotum with a yellowish median stripe, posterior margin covered by golden setae; metacetabulum with a lateral stripe of golden setae. *Abdomen*: medio- and laterotergites black; lateral margins of mediotergites with a longitudinal stripe of golden setae; side of abdomen with a longitudinal stripe of golden setae. *Male*: posterior margin of abdominal mediotergite VII rounded. Posterior projection of last abdominal laterotergite (= connexival spine) triangular. Abdominal sterna II–VII without median depression. Abdominal sternum VII with a rounded notch on posterior margin, notch subequal in length and width (Fig 25F). Abdominal segment VIII 1.7–2.2 times longer than wide, tubular; lateral margins slightly divergent posteriorly, anterior width 9/10 of posterior width; right side with a pair of long, narrow projections (about three times as long as proximal width), projections inserted together by the base, directed posteroventrally, with apex rounded to acute; venter with a median depression on anterior 2/3 to whole segment (Fig 25L); posteroventral margin rounded, forming a large, sinistrally directed, rounded notch. Pygophore and proctiger sinistrally rotated about 90°. Pygophore about 2.4 times longer than tergum VII. Proctiger 1.3 times longer than abdominal tergum VII; anterior margin with a long notch, notch with 1/3 of proctiger length; right margin folded ventrally; left margin strongly folded on left basolateral process up to about middle of proctiger; apex without denticles; anal cone inserted posteriorly; right basolateral process developed, shorter than 1/5 of left basolateral process; left basolateral process long, about twice as long as wide, 2.2 times longer than proctiger, longitudinal axis diverging about 100° from longitudinal axis of proctiger, lateral margins converging distally, apex acute, not bifid, not reaching left margin of abdominal segment VIII. Phallus long, about 6 times longer than smallest width; dorsal sclerite anteriorly coiled, slightly narrower and much longer than ventral sclerite, base not notched; lateral sclerite triangular; base of ventral sclerite bifid, apex rectangular; transverse sclerite not visible. Abdominal segment VIII black dorsally, yellowish laterally and ventrally; pygophore yellowish on anterior 1/2, brownish on posterior 1/2; proctiger black, apex yellowish. *Female*: abdominal tergum VIII triangular, lateral margins strongly diverging posteriorly, posterior angle sharp. Anal cone short. Abdominal laterotergites not folded over mediotergites; posterior projection of last laterotergite (= connexival spine) long, tubular, subequal in length to mediotergite VII, apex rounded. Expansion of female abdominal sternum VII completely covering first pair of gonocoxae, partially folded over tergum VIII, with a lobule (Fig 25P). Abdominal tergum VIII blackish, with yellowish lateral margins; expansion of sternum VII yellowish, apex slightly darker. *Macropterous*: 1/3 to 2/3 of posterolateral margins and whole posterior margin of pronotum yellowish.

#### Variation

The dorsum of the head can be from yellow with a black mark on the frons to black with a yellow mark on the vertex. The middle and hind trochanters can have the proximal 2/3 black and the apex yellowish. The apex of female abdominal tergum VIII can be posteriorly prolonged and curved.

#### Comments

The male of *P*. *tridentatus* has the pygophore and proctiger sinistrally rotated about 90°, similar to *P*. *anchicaya*, *P*. *bilobulatus*, *P*. *carvalhoi*, *P*. *molanoi* Floriano and Moreira, **sp. nov.**, *P*. *peruvianus*, *P*. *shuar*, *P*. *spiculus*, *P*. *sumaco*, *P*. *variabilis*, *P*. *vivatus*, *P*. *williamsi*, and *P*. *woytkowskii*. It differs from most of these species, except for *P*. *anchicaya*, *P*. *sumaco*, and *P*. *williamsi*, by the pair of projections on the right margin of abdominal segment VIII (Fig 25L). Both *P*. *sumaco* and *P*. *williamsi* can be distinguished from *P*. *tridentatus* because their projections are inserted on the abdominal segment separately (Fig 9B–9E), whereas in the last species they are connected by their bases. As for *P*. *anchicaya*, it differs from *P*. *tridentatus* by the combination of: (1) notch on the posterior margin of male abdominal sternum VIII shallow in the former (Fig 1B), but much more pronounced and sinistrally directed in the latter (Fig 25F); and (2) left basolateral process of male proctiger positioned over or under the projections of abdominal segment VIII in the former (Fig 1B), but not reaching the projections in the latter due to a sinistral dislocation of the proctiger (Fig 25F). The second character above must be used with caution, because the proctiger is mobile, which can alter its position in relation to the rest of the abdomen.

**Geographic distribution (Fig 38).** COLOMBIA: **Cauca** (Gorgona Island) [29, 31], **Chocó** [31], **Valle del Cauca** [29–31]. COSTA RICA: **Alajuela** [33], **Heredia** [33, this work], **Guanacaste** [6, 33, this work], **Limón** [33], **Puntarenas** [6, 10, 33, 58, this work], **San José** [17, 33]. PANAMA: **Chiriquí** [6 this work], **Panama** [14, 17, 59], **Veraguas** [52].

#### Material examined

1♀, 1♂ (MZUCR): ‘Costa Rica, Heredia,\ Sarapiqui\ Reserva la Tirimbina\ curso Singapur 2012\ Col. M. Springer’ ‘Gerridae\ *Potamobates*\ *tridentatus*\ Det. B. Pacheco’. 1♀, 1♂ (USNM): ‘Costa Rica\ Surubres’ ‘p.San Mates.\ Pacif. 250m.\ P. Biolley’ ‘G. W. Kirkaldy\ 1919\ Collection’. 2♀, 1♂ (USNM): ‘Guanacaste\ Costa Rica\ 12 VII 57\ DR Lauck’ ‘C J Drake\ Coll. 1956’. 4♀, 2♂ (USNM): ‘Costa Rica: Heredia,\ La Selva Field Sta.\ near Puerto Viejo\ 21–28 March 1988’ ‘W. E. Steiner\ J. M. Hill\ J. M. Swearingen\ J. M. Mitchell’. 3♀, 4♂ (USNM): ‘N. of Esparta\ Costa Rica, CL\ 1264 XII–24–1969\ J. T. Polhemus’ ‘J. T. Polhemus\ Collection 2014\ C. J. Drake Accession’. 1♀, 2♂ (USNM): ‘Ernesto Barrera y\ Harry Brailovsky, Col.\ Cañas\ Rio Lagarto\ Prov. Puntarenas\ Costa Rica\ 9-11-81’ ‘Colección del Instituto\ de Biologia, UNAL. México, D.F.’ ‘J. T. Polhemus\ Collection 2014\ C. J. Drake Accession’. 5♀, 1♂ (USNM): ‘Panama, Chiriqui Prov.\ rocky stream at Balnearo\ 3.8 km. N. of David, 50m\ 13 January 1993 CL 2819\ J. Polhemus & A. Gillogly’ ‘J. T. Polhemus\ Collection 2014\ C. J. Drake Accession’. 5♂, 1♀ (AMNH): ‘Costa Rica: Puntarenas Prov.\ Rincon de Osa, Osa Peninsula\ Rio Aguabuena, 14–26July1969\ Toby Schuh, Janet Crane’.

### *Potamobates unidentatus* Champion, 1898

(Figs 10 and 24)

*Potamobates unidentatus* Champion, 1898: 154, 155, 453 (description, figure). Esaki (1926) [10]: 251 (note). Drake and Harris (1934) [21] 224, 227 (key, redescription). Kuitert (1942) [17]: 140, 141 (key, note). Matsuda (1960) [2]: 512, 513 (figure). Polhemus and Polhemus (1995) [6]: 360–362, 364, 366–368, 372 (figure, key, phylogeny, redescription). Cognato (1998) [18]: 21, 22 (phylogeny). Buzzetti (2006) [19]: 55 (phylogeny). Padilla-Gil and Damgaard (2011) [7]: 44, 46 (key, phylogeny). Morales et al. (2013) [5]: 194 (figure).

#### Diagnosis

Male pygophore and proctiger sinistrally rotated about 20° (Fig 10A, 10D and 10E); left posterolateral angle of male abdominal segment VIII with a ventral projection, projection subequal in length and width (Fig 10D); female abdominal tergum VIII subequal in length to mediotergite VII, with lateral margins curved ventrally (Fig 10B); expansion of female abdominal sternum VII slightly shorter than mediotergite VII, triangular, subequal in length and width (Fig 10C).

#### Redescription

Length (**♂**: 8.4; ♀: 9.2); width (**♂**: 2.19; ♀: 2.66) (Fig 24). *Head*: Antennomere I 1.2 times longer than head width, 2.4–3.0 times longer than antennomere II; II with 1.0–1.1 times antennomere III length; IV 1.3–1.4 times longer than III. Eye width 1.4 times larger than interocular width. Article III of labium 1.8–2.1 times longer than article IV. Antenna black; dorsum of head yellow; frontoclypeus with an oval blackish mark; vertex with lateral margins blackish; antenniferous tubercle yellow; labium with articles I–II and basal 1/2 of III yellow, remainder of labium blackish; venter of head yellowish. *Thorax*: Pronotum with a triangular yellowish mark, posterior margin not covered by golden setae; propleuron with a patch of golden setae; proacetabulum with a lateral stripe of golden setae and a ventral blackish mark; fore coxa, trochanter, and femur yellowish, femur with apex and venter blackish; fore tibia and tarsus blackish. Mesonotum with a yellowish median mark and a pair of yellowish lateral marks on posterior 2/3, posterior margin with golden setae; dorsal 3/4 of spiracle covered by golden setae; mesopleuron with a longitudinal stripe of golden setae, stripe sigmoid, curved, posteriorly discontinue; mesosternum yellow; middle and hind coxae yellowish; middle and hind trochanters black dorsally, yellowish ventrally; middle and hind femora blackish, middle femur with a pair of yellow stripes on basal 2/3; middle and hind tibiae and tarsi yellowish. Metanotum with a median yellowish stripe; metacetabulum with a stripe of golden setae. *Abdomen*: most of medio- and laterotergites black, laterotergites dorsally yellowish. *Male*: posterior margin of abdominal mediotergite VII rounded. Posterior projection of last abdominal laterotergite (= connexival spine) rounded at apex (Fig 10A). Abdominal sterna II–VII without median depression. Abdominal sternum VII with an oval notch on posterior margin, notch almost three times as wide as long. Abdominal segment VIII subequal in length and width, tubular; lateral margins slightly converging posteriorly, anterior width 1.2 times larger than posterior width; posterodorsal margin rounded; venter with a depression on anterior 1/2; left posteroventral margin with a lateral triangular projection. Pygophore and proctiger sinistrally rotated about 20° (Fig 10A, 10D and 10E), each twice as long as abdominal mediotergite VII. Proctiger with a short notch on the anterior margin, notch about 1/5 of proctiger length; lateral margin not folded ventrally; left margin folded only on left basolateral process; apex with denticles; right basolateral process not developed; left basolateral process length 4/5 of its width, 1/3 of proctiger length, longitudinal axis diverging about 90° of longitudinal axis of proctiger, lateral margins converging distally, apex obtuse, not bifid (Fig 10I and 10J). Phallus short, about three times as long as its smallest width (Fig 10F); dorsal sclerite slightly narrower and longer than ventral sclerite, base not notched (Fig 10H); lateral sclerite triangular; ventral sclerite diverging posteriorly, base bifid, apex simple, not expanded (Fig 10H); transverse sclerite not visible. Dorsum of abdominal segment VIII black, venter yellowish; pygophore yellowish; proctiger black, left basolateral process brown. *Female*: abdominal tergum VIII triangular, as long as wide, lateral margins strongly diverging posteriorly and curved ventrally, posterior margin truncate (Fig 10B and 10C). Abdominal laterotergites not folded over mediotergites (Fig 24C). Posterior projection of last abdominal laterotergite (= connexival spine) triangular (Fig 10B). Extension of female abdominal sternum VII completely covering first pair of gonocoxae, not folded over tergum VIII (Fig 10B and 10C). Anal cone short. Abdominal segment VIII dorsally black, lateral margins and venter yellowish. *Macropterous*: posterior 4/5 of posterolateral margins and entire posterior margin of pronotum yellowish.

#### Variation

Some specimens from Puntarenas (Costa Rica) have the projection on the posterolateral angle of male abdominal segment VIII narrower than in most individuals.

#### Comments

The male of *P*. *unidentatus* has the pygophore and proctiger weakly rotated, similar to *P*. *bidentatus*, *P*. *horvathi*, *P*. *manzanoae*, and *P*. *osborni*. It can be distinguished from all these species by the pygophore and proctiger even less rotated (Fig 10A, 10D and 10E). It differs further from *P*. *bidentatus*, *P*. *horvathi*, and *P*. *osborni* by the triangular mark on the pronotum (Fig 24A and 24C), whereas the others have a longitudinal stripe (compare Figs 15A, 15C, 16A, 16C, 22A and 22C). The female of *P*. *unidentatus* is separated from all these other species by the abdominal tergum VIII with the lateral margins curved ventrally (Fig 10B and 10C).

**Geographic distribution (Fig 36).** COLOMBIA: **Chocó** [45 this work]. COSTA RICA: **Alajuela** [6, 33, 60, this work], **Cartago** [33], **Guanacaste** [33], **Heredia** [33], **Limón** [6, 33], **Puntarenas** [6, 33 this work], **San José** [17, 33, 51, 59, this work]. PANAMA: **Bocas del Toro** [6], **Chiriquí** [8], **Coclé** [6], **Kuna Yala** [6], **Panama** [6]. Records from Meta Department, Colombia [30, 47], are based on misidentifications. Those from Magdalena Department, Colombia [10], and Monagas State, Venezuela [43], need verification.

#### Material examined

1♂ (USNM): ‘San Antonio\ COSTA RICA, CL\ 1269 XII-25-1969\ J. T. Polhemus’ ‘*Potamobates*\ *unidentatus*\ Champion\ det. J. T. Polhemus’. 1♂ (USNM): ‘San Antonio\ COSTA RICA, CL\ 1269 XII-25-1969\ J. T. Polhemus’. 1♂, 2♂, 1 nymph (UPTC): ‘Ordem Hemiptera\ Familia Gerridae\ Genero *Potamobates*\ Especie *Potamobates unidentatus*\ Descrimino: Estupiñan A’ ‘Colombia Choco Acandi Capurgana\ 8° 37’ 37.89”N 77°20’53.16” O 15ra\ Cascada cerca de Sapzurro Colecta\ Jama Acuática. 15-I-2008. Estupiñan, Lopez & Molano’ ‘P. un. 0022’. 1♂ (UPTC): ‘Costa Rica\ José San Ramon\ de três Rios 1400 m\ Rio Puruses poca\ Corriente 23/08/98\ Co: X. Miranda’ ‘Microptera’ ‘*Potamobates*\ *unidentatus*\ Det. F. Molano’ ‘P. um. 0020’. 1♂ (UPTC): ‘Costa Rica Miranar V\ Rio Pedro 700 m\ 31/oct/90\ col: Guilhermo’ ‘P. un. 0021’ ‘*Potamobates*\ *unidentatus*\ Det: F. Molano’. 1♀, 1♂ (UPTC): ‘Costa Rica Reserva\ San Ramón Alajuela\ 700 m. 23.03.2007 Col.\ B. Pacheco y M. Zunbado’ ‘*Potamobates*\ *unidentatus*’ ‘P. un. 0071’. 2♀, 4♂ (AMNH): ‘Costa Rica: Puntarenas Prov.\ Rincon de Osa, Osa Peninsula\ Rio Aguabuena, 14–26July1969\ Toby Schuh, Janet Crane’ ‘Donation from\ J. A. Slater\ Collection’.

### *Potamobates variabilis* Hungerford, 1938

(Figs 11 and 41)

*Potamobates variabilis* Hungerford, 1938 [15] (description, figure). Polhemus and Polhemus (1995) [6]: 365, 367, 368, 372 (figure, key, phylogeny, redescription). Cognato (1998) [18]: 21, 22 (phylogeny). Buzzetti (2006) [19]: 55 (phylogeny). Padilla-Gil and Damgaard (2011) [7]: 45, 46 (key, phylogeny).

#### Diagnosis

Male pygophore and proctiger sinistrally rotated about 90° (Fig 11A and 11B); right posterolateral angle of male abdominal segment VIII with a single or a pair of projections (Fig 11B–11D); posterior margin of male abdominal sternum VIII with a weak notch; left basolateral process of male proctiger with apex bifid (Fig 11A, 11B and 11H); posterior projection of last female abdominal laterotergite (= connexival spine) long, subequal in length to mediotergite VII; extension of female abdominal sternum VII subequal in length to mediotergite VII, with a lobule shorter than 1/2 of sternum VII length.

#### Redescription

Length (**♂**: 10.5; ♀: 8.7); width (**♂**: 2.8; ♀: 2.7) (Fig 41). *Head*: antennomere I 1.0–1.2 times longer than head width, 3.0 times longer than antennomere II; II subequal in length to III; IV 1.4–1.5 times longer than III. Eye width 1.4–1.8 times larger than interocular width. Article III of labium 1.8–2.0 times longer than article IV. Antenna black; dorsum of head yellow; frons with a blackish mark; vertex unmarked; antenniferous tubercle yellowish; mandibular and maxillary plates yellowish; labium with articles I–II and proximal 2/3 of III yellow, remainder of III and entire IV blackish; venter of head yellowish. *Thorax*: Pronotum with a yellowish wedge-shaped mark, posterior margin sometimes with golden setae; propleuron with an anterior patch of golden setae; proacetabulum with a lateral stripe of golden setae, posterior margin sometimes with a blackish mark; fore coxa, trochanter and dorsum of femur yellowish; venter of femur, entire tibia and tarsus black. Mesonotum black; dorsal 3/4 of spiracle covered by golden setae; mesopleuron with a longitudinal stripe of golden setae, stripe sigmoid, curved, posteriorly discontinue; mesosternum without marks; middle and hind coxae and trochanters yellowish, trochanters with proximal 2/3 and venter with a black mark; middle and hind femora with sides yellowish; middle and hind tibiae and tarsi yellowish to brown, middle tibia sometimes with blackish stripes. Metanotum black, posterolateral margin covered by golden setae; metacetabulum with a stripe of golden setae. *Abdomen*: medio- and laterotergites black, lateral margins of mediotergites with a longitudinal stripe of golden setae; side of abdomen yellowish, with black marks and a longitudinal stripe of golden setae. *Male*: posterior margin of abdominal mediotergite VII rounded. Posterior projection of last abdominal laterotergite (= connexival spine) triangular. Abdominal sterna II–VII without median depression. Abdominal sternum VII with a rounded notch on posterior margin, notch subequal in length and width. Abdominal segment VIII 1.4–1.6 times longer than wide, tubular; lateral margins slightly divergent posteriorly, anterior width 9/10 of posterior width; right side with a variable projection, projection 2.5–4.0 times longer than wide, with lateral margins parallel and apex bifid (Fig 11B); in some specimens, an additional dorsalmost projection is present, with apex rounded to acute and no more than half of ventralmost projection length; single or pair of projections directed posteroventrally, when in pair, bases connected (Fig 11B–11D); venter with a median depression for about anterior half of segment; posteroventral margin rounded, not forming a notch. Pygophore and proctiger sinistrally rotated about 90° (Fig 11A and 11B). Pygophore about 2.3 times longer than abdominal mediotergite VII. Proctiger subequal in length to abdominal mediotergite VII; anterior margin with a long notch, notch with 1/3 of proctiger length; right margin folded ventrally; left margin strongly folded on left basolateral process up to the middle of proctiger; apex without denticles; right basolateral process developed, rounded and short; left basolateral process long, about twice as long as wide, subequal to proctiger in length, longitudinal axis diverging about 100° from longitudinal axis of proctiger, lateral margins diverging distally, apex bifid (Fig 11H). Phallus long, about 5.3 times longer than smallest width (Fig 11E); dorsal sclerite anteriorly coiled, slightly narrower and much longer than ventral sclerite, base not notched (Fig 11F); lateral sclerite triangular; base of ventral sclerite bifid, apex slightly wider than basal area (Fig 11G); transverse sclerite visible (Fig 11E). Abdominal segment VIII dorsally black, side and venter yellowish; pygophore yellowish; proctiger brown. *Female*: abdominal tergum VIII 1.2 times longer than wide, lateral margins strongly diverging posteriorly; anal cone short. Abdominal laterotergites not folded over mediotergites, except on last segment (Fig 41C); posterior projection of last abdominal laterotergite (= connexival spine) long, tubular, subequal in length to mediotergite VII, apex rounded (Fig 41C). Posterior margin of abdominal sternum VII truncate. Extension abdominal of sternum VII oval, completely covering first pair of gonocoxae, partially folded over tergum VIII, about twice as wide as long, with an oval lobule on right side (Fig 41D). Abdominal tergum VIII black with yellowish lateral margins; expansion of abdominal sternum VII yellowish.

#### Variation

The antenna can be brownish, with the antennomere I and proximal 2/3 of II yellow. The dorsum of the head can be from entirely yellow to mostly black with only the vertex yellowish. The pronotum can have a triangular mark or be yellow with a black anterolateral mark. The fore trochanter can be entirely yellow, the fore tibia can have the basal half yellowish dorsally, and the black stripes of the middle and hind femora can be narrower in some specimens than in others. The mesonotum can have a yellowish mark on posterolateral 1/3 and a median stripe on posterior 1/2. The abdominal mediotergites can have a yellow mark centrally. The abdominal laterotergites can be entirely yellow.

#### Comments

The male of *P*. *variabilis* has the pygophore and proctiger sinistrally rotated about 90° (Fig 11A and 11B), similar to *P*. *anchicaya*, *P*. *bilobulatus*, *P*. *carvalhoi*, *P*. *molanoi* Floriano and Moreira, **sp. nov.**, *P*. *peruvianus*, *P*. *shuar*, *P*. *spiculus*, *P*. *sumaco*, *P*. *tridentatus*, *P*. *vivatus*, *P*. *williamsi*, and *P*. *woytkowskii*. It can be easily distinguished from all these species based on the bifid apex of the left basolateral process of the proctiger (Fig 11H).

**Geographic distribution (Fig 40).** PERU: **Huánuco** [15, this work]. The record from Valle del Cauca Department, Colombia [45] needs to be verified.

#### Material examined

14♀, 2♂ (USNM): ‘Cucharas, R.\ Huallaga, Peru\ FL W VIII 1954’. 19♂ (USNM): ‘Cucharas R.\ Pacaya, Peru\ FL WVI-1954’ ‘CJDrake\ Coll.1956’. 1♂ (USNM): ‘*Potamobates*\ *variabilis*\ Hung. (1938)\ Det. J. C. Lutz’ ‘Peru: Puente Perez\ Riv. Trib. of Huallaga Riv. S. W’ ‘of Tingo Maria.’ ‘X. 2. 1955’ ‘S.S. Roback. (Coll)’ ‘J C Lutz\ Collection\ 1961’. 2♀, 2♂ (USNM): ‘Tingo Maria,\ Peru IX-16-44\ E.J.Hambleton’ ‘C J Drake\ Coll. 1956’. 1♀, 1♂ (USNM): ‘Shapajilla, Huanuco\ Peru June, 1939\ Felix Woytkowski’ ‘C J Drake\ Coll. 1956’.

### *Potamobates vivatus* Drake and Roze, 1954

(Figs 27 and 33)

*Potamobates vivatus* Drake and Roze, 1954 [10]: 228, 229 (description). Cognato (1998) [18]: 21, 22 (phylogeny). Buzzetti (2006) [19]: 55 (phylogeny). Padilla-Gil and Damgaard (2011) [7]: 45, 46 (key, phylogeny). Morales et al. (2013) [5]: 194 (figure).

#### Diagnosis

Male pygophore and proctiger sinistrally rotated about 90° (Fig 27); right posterolateral angle of male abdominal segment VIII with a dorsal projection, projection 2.2–3.3 times longer than wide (Fig 27B); female abdominal tergum VIII long, 4.1–4.3 times longer than wide.

#### Redescription

Length (**♂**: 11.7; ♀: 11.8); width (**♂**: 2.8; ♀: 3.1) (Fig 33). *Head*: antennomere I 1.2 times longer than head width, 2.8–3.0 times longer than antennomere II; II subequal in length to III; IV 1.3 times longer than III. Eye width 1.4 times interocular width. Article III of labium 2.3 times longer than article IV. Antenna black; dorsum of head yellowish, frons with a brownish mark, antenniferous tubercles slightly darker; mandibular and maxillary plates yellowish; labium with articles I–II and base of III yellowish, remainder of III and entire IV blackish; venter of head yellowish. *Thorax*: Pronotum with a yellowish wedge-shaped mark, posterior margin not covered by golden setae; propleuron with an anterior patch of golden setae; proacetabulum yellowish, posterior margin brownish; fore coxa, trochanter, and femur yellowish, femur with venter and apex blackish; fore tibia and tarsus blackish, tibia usually brown distally on dorsum. Mesonotum blackish with a yellow median stripe posteriorly; dorsal 3/4 of spiracle covered by golden setae; mesopleuron with a longitudinal stripe of golden setae, stripe sigmoid, curved, posteriorly discontinue; mesosternum without marks; middle and hind coxae, trochanters, and femora yellow, femora with a black stripe dorsally and ventrally; middle and hind tibiae and tarsi yellowish to brownish. Metanotum without stripes, posterolateral margin with golden setae; metapleuron and metasternum yellowish; metacetabulum with a longitudinal blackish stripe and a stripe of golden setae. *Abdomen*: medio- and laterotergites black, lateral margins of mediotergites with longitudinal stripe of golden setae. *Male*: posterior margin of abdominal mediotergite VII rounded. Posterior projection of last abdominal laterotergite (= connexival spine) triangular. Abdominal sterna II–VII slightly depressed medially, more conspicuously on posterior segments (Fig 33B). Sternum VII with a rounded notch on posterior margin, notch subequal in length and width. Abdominal segment VIII twice as long as wide, tubular; lateral margins slightly divergent posteriorly, anterior width 4/5–9/10 of posterior width; right side with a long, narrow projection, projection 2.0–3.3 times longer than wide, directed posteroventrally, with acute apex (Fig 27B); venter with an anterior median depression reaching middle of segment, posteroventral margin rounded. Pygophore and proctiger sinistrally rotated about 90° (Fig 27A and 27B). Proctiger 1.4 times longer than abdominal mediotergite VII; anterior margin with a long notch, notch with 1/3 of proctiger length; lateral margins not folded ventrally; apex without denticles; right basolateral process developed, rounded, length about 1/5 of left basolateral process; left basolateral process long, about twice as long as wide, with 4/5 of proctiger length, longitudinal axis diverging about 90° from longitudinal axis of proctiger, lateral margins convergent distally, apex acute, not bifid (Fig 27A). Abdominal segment VIII black dorsally, yellowish ventrally; pygophore yellowish, apex brownish; proctiger dark brown, base of left basolateral process yellowish, apex black. *Female*: abdominal laterotergites not folded over mediotergites; posterior projection of last abdominal laterotergite (= connexival spine) long, tubular, 1.5–1.6 times longer than mediotergite VII, apex rounded (Fig 33C). Abdominal tergum VIII triangular, 4.1–4.3 times longer than wide, lateral margins strongly converging posteriorly, posterior angle acute, anal cone short. Extension of abdominal sternum VII oval, about twice as wide as long, completely covering first pair of gonocoxae, not folded over tergum VIII, with an oval lobule. Abdominal tergum VIII and extension of abdominal sternum VII yellowish, tergum blackish on a central mark anteriorly and on posterior 2/3.

#### Variation

The base and the apex of antennomere IV can be slightly lighter. Male abdominal tergum VIII can have a central yellow stripe or mark posteriorly.

#### Comments

The male of *P*. *vivatus* has the pygophore and proctiger sinistrally rotated about 90° (Fig 27A and 27B), similar to *P*. *anchicaya*, *P*. *bilobulatus*, *P*. *carvalhoi*, *P*. *molanoi* Floriano and Moreira, **sp. nov.**, *P*. *peruvianus*, *P*. *shuar*, *P*. *spiculus*, *P*. *sumaco*, *P*. *tridentatus*, *P*. *variabilis*, *P*. *williamsi*, and *P*. *woytkowskii*. It can be distinguished from all these species by the dorsal, 2.0–3.3 times longer than wide, projection on the right posterolateral angle of male abdominal segment VIII, and the female abdominal tergum VIII long, 4.1–4.3 times longer than wide (Fig 33D).

**Geographic distribution (Fig 39).** VENEZUELA: **Carabobo** [6, 10, this work], **Distrito Federal** (17; 55]. Records of this species from Colombia [1, 6, 30, 32, 45, 36–38, 53] are based on misidentifications of *P*. *molanoi* Floriano and Moreira, **sp. nov.**

#### Type material examined

2♀, 9♂ paratypes (USNM): ‘Belén, Venez.\ Carabobo\ VI–27–1953’ ‘Paratype\ *Potamobates*\ *vivatus*\ D & R.’.

### *Potamobates williamsi* Hungerford, 1932

(Figs 28 and 34)

*Potamobates williamsi* Hungerford, 1932 [12]: 228–230 (description). Drake and Harris (1934) [21]: 224, 228, 229 (key, redescription). Hungerford (1937b) [14]: 146, 147 (figure). Kuitert (1942) [61]: 140, 141 (key, note). Matsuda (1960) [2]: 512, 513 (figure). Polhemus and Polhemus (1995) [6]: 363, 365, 367–369, 372 (figure, key, phylogeny). Cognato (1998) [18]: 19–22 (figure, key, phylogeny, redescription). Buzzetti 2006 [19]: 55 (phylogeny). Padilla-Gil and Damgaard (2011) [7]: 45, 46 (key, phylogeny).

#### Diagnosis

Male pygophore and proctiger sinistrally rotated about 90° (Fig 28A–28D); male abdominal segment VIII with a pair of projections on right side, projections about three times as long as wide, inserted separately (Fig 28C); left basolatoral process of male proctiger with a 90° twist throughout length (Fig 28B); expansion of female abdominal sternum VII not folded over tergum VIII (Fig 34D).

#### Redescription

Length (**♂**: 10.4–11.1; ♀: 8.6–10.0); width (**♂**: 2.5–2.9; ♀: 2.7–3.1) (Fig 34). *Head*: antennomere I 1.1 times longer than head width, 2.5–2.8 times longer than antennomere II; II 1.1 times longer than III; IV 1.4–1.7 times longer than III. Eye width 1.7 times larger than interocular width. Article III of labium 1.8–2.0 times longer than article IV. Antenna black; dorsum of head yellow, frons with a blackish mark; antenniferous tubercle blackish; mandibular and maxillary plates yellowish; labium with articles I–II and anterior 2/3 of III yellow, remainder of III and entire IV blackish; venter of head yellowish. *Thorax*: Pronotum with a brown wedge-shaped mark, posterior margin covered by golden setae; propleuron with an anterior patch of golden setae; proacetabulum yellow, with a lateral stripe of golden setae and a blackish mark on posterior margin; fore coxa and trochanter yellowish, trochanter sometimes with a blackish stripe ventrally; fore femur yellowish, venter and apex blackish; tibia and tarsus black. Mesonotum usually with a yellowish median stripe, a pair of triangular marks on posterior 1/3, posterior margin covered by golden setae; dorsal 3/4 of spiracle covered by golden setae; mesopleuron with a longitudinal stripe of golden setae, stripe sigmoid, curved, posteriorly discontinue; mesosternum without marks; mesoacetabulum with a lateral stripe of golden setae; middle and hind coxae and trochanters yellowish, trochanters with proximal 2/3 blackish; middle and hind femora yellowish with a blackish stripe on dorsum and venter; middle and hind tibiae and tarsi brownish, lighter towards apex. Metanotum with a median yellow stripe, posterior angle covered by golden setae; metacetabulum with a lateral stripe of golden setae; metasternum without marks. *Abdomen*: medio- and laterotergites I–VII black, lateral margins of mediotergites with a longitudinal stripe of golden setae, mediotergites II–VII sometimes with a yellowish mark centrally; side of abdomen with a longitudinal stripe of golden setae. *Male*: posterior margin of abdominal mediotergite VII rounded. Posterior projection of last abdominal laterotergite (= connexival spine) triangular (Fig 28A). Abdominal sterna II–VII without median depression. Sternum VII with a rounded notch on posterior margin, notch subequal in length and width. Abdominal segment VIII 1.5–1.7 times longer than wide, tubular; lateral margins slightly diverging posteriorly, anterior width 9/10 of posterior width; right side with a pair of long, narrow projections, projections about three times as long as wide at base, inserted separately, directed posteroventrally, with rounded to acute apex (Fig 28C); venter with an anterior median depression reaching middle of segment, posteroventral margin rounded. Pygophore and proctiger sinistrally rotated about 90° (Fig 28A–28D). Pygophore about 2.5 times longer than mediotergite VII. Proctiger 1.2 times longer than mediotergite VII; anterior margin with a long notch, notch with 1/3 of proctiger length; right margin folded ventrally; left margin strongly folded on left basolateral process up to about the middle of proctiger; apex without denticles; right basolateral process developed, rounded, length about 1/5 of left basolateral process; left basolateral process long, about twice as long as wide, smaller than proctiger length, longitudinal axis diverging about 100° from longitudinal axis of proctiger, lateral margins converging distally; apex acute, not bifid (Fig 28B). Phallus long, about 6 times as long as smallest width; dorsal sclerite anteriorly coiled, slightly narrower and much longer than ventral sclerite, base not notched; lateral sclerite triangular; base of ventral sclerite bifid, apex slightly wider; transverse sclerite not visible. Abdominal segment VIII black dorsally, yellowish laterally and ventrally; pygophore with anterior 1/2 yellowish and posterior 1/2 brownish; proctiger black, apex yellowish. *Female*: abdominal tergum VIII triangular, lateral margins strongly diverging posteriorly, posterior margin rounded (Fig 34C). Abdominal laterotergites not folded over mediotergites or only over mediotergite VII (Fig 34C); posterior projection of last abdominal laterotergite (= connexival spine) long, tubular, longer than mediotergite VII, apex rounded (Fig 34C). Expansion of abdominal sternum VII completely covering first pair of gonocoxae, subequal in width and folded over tergum VIII, with a lobule (Fig 34D). Anal cone short. Abdominal tergum VIII yellowish, blackish anteriorly; expansion of sternum VII yellowish, slightly darker posteriorly.

#### Variation

The antenna can be brownish. The dorsum of the head can vary from blackish with a yellowish mark on the vertex to entirely yellowish. The article III of labium can have the proximal 1/3 or 2/3 yellowish and the remainder blackish. The fore trochanter can be entirely yellowish. The mesonotum can be entirely black or bear a yellowish median stripe and marks on the posterolateral 1/3, the stripe and marks can vary in length and width. The middle and hind trochanters can be uniformly yellow or have a weak brownish mark ventrally. In darker specimens, the middle and hind femora are black. The metanotum can be completely black or have a large yellowish central stripe. The abdominal tergum VIII of the male can display a central yellowish mark.

#### Comments

The male of *P*. *williamsi* has the pygophore and proctiger sinistrally rotated about 90° (Fig 28A–28D), similar to *P*. *anchicaya*, *P*. *bilobulatus*, *P*. *carvalhoi*, *P*. *molanoi* Floriano and Moreira, **sp. nov.**, *P*. *peruvianus*, *P*. *shuar*, *P*. *spiculus*, *P*. *sumaco*, *P*. *tridentatus*, *P*. *variabilis*, *P*. *vivatus*, and *P*. *woytkowskii*. It can be distinguished from all these species, except for *P*. *anchicaya*, *P*. *sumaco*, and *P*. *tridentatus*, by the right posterolateral margin of male abdominal segment VIII with a pair of long projections (Fig 28C). However, both *P*. *anchicaya* and *P*. *tridentatus* have these projections inserted on the abdominal segment together, whereas they are inserted separately in *P*. *sumaco* and *P*. *williamsi*. *Potamobates williamsi* is very similar to *P*. *sumaco*, and the characters separating these two were commented under *P*. *sumaco*.

**Geographic distribution (Fig 40).** ECUADOR: **Napo** [12, 14, 17, 18, 57, this work], **Orellana** [18, 57], **Pastaza** [12, 17, 18].

#### Type material examined

3♀, 3♂ paratypes (USNM): ‘PARATYPE\ *Potamobates*/*\ williamsi*\ H. B. hungerford’ ‘F. X. Williams\ Tena, Ecuador/ Feb.23, 1923’ ‘Paratype No.\ 52008\ U.S.N.M.’ ‘♂’.

#### Additional material examined

1♂, 1♀ (USNM): ‘Ecuador S. A\ Mar. 1937\ Clarke MacIntyre’ ‘Oriente, E\ Rio Napo water\ shed Jatun\ Yacu 700 mtrs’ ‘C J Drake\ Coll. 1956’. 1♂, 1♀ (USNM): ‘Ecuador\ Napo Prov. Shell\ X-7-79\ J.J Anderson’ ‘J. T. Polhemus\ Collection 2014\ C. J. Drake Accession’. 6♂, 2♀ (USNM): ‘Ecuador S. A\ Mar. 29 1937\ Clarke MacIntyre’ ‘Oriente, E\ Rio Napo water\ shed Jatun\ Yacu 700 mtrs’ ‘J. T. Polhemus\ Collection 2014\ C. J. Drake Accession’. 1♂, 1♀ (AMNH): ‘Ecuador S. A.\ Mar. 29 1937\ Clarke MacIntyre’ ‘Oriente E\ Rio Napo water\ shed Jatun\ Yacu 700 mtrs’.

### *Potamobates woytkowskii* Hungerford, 1937

(Figs 19 and 35)

*Potamobates woytkowskii* Hungerford, 1937b [14]: 144, 145, plate II (description, figure). Polhemus and Polhemus (1983) [16]: 287 (figure, note). Polhemus and Polhemus (1995) [6]: 365, 367–369, 372 (key, phylogeny). Cognato (1998) [18]: 21, 22 (phylogeny). Buzzetti (2006) [19]: 55 (phylogeny). Padilla-Gil and Damgaard (2011) [7]: 45, 46 (key, phylogeny).

#### Diagnosis

Male pygophore and proctiger sinistrally rotated about 90° (Fig 19A and 19C); right posterolateral angle of abdominal segment VIII with a pair of projections ventrally, dorsalmost projection small and about as long as wide, ventralmost projection oval and about twice as long as wide (Fig 19C–19F); posterior margin of male abdominal sternum VIII with a large notch; female abdominal tergum VIII subequal in length and width; posterior projection of last female abdominal laterotergite (= connexival spine) long, three times longer than mediotergite VII (Fig 19C); expansion of female abdominal sternum VII with a long lobule, lobule subequal in length to sternum VII (Fig 19B).

#### Redescription

Length (**♂**: 10.8–11.5; ♀: 10.8); width (**♂**: 2.7–2.8; ♀: 3.1) (Fig 35). *Head*: antennomere I about 1.2 times longer than head width, 3 times longer than antennomere II; II subequal in length to III; IV 1.3–1.4 times longer than III. Eye width 1.3–1.5 times larger than interocular width. Article III of labium 1.6 times longer than article IV. Antenna brownish to blackish, antennomere I slightly lighter; dorsum of head yellow, frons with a blackish mark, vertex unmarked; antenniferous tubercle, and mandibular and maxillary plates yellowish; labium with articles I–II and proximal 2/3 of III yellow, remainder of III and entire IV blackish; venter of head yellowish. *Thorax*: Pronotum with a yellow wedge-shaped mark, posterolateral angle with some golden setae; propleuron with an anterior patch of golden setae; proacetabulum yellow, with a lateral stripe of golden setae and posterior margin brownish; fore coxa and trochanter yellowish, venter of trochanter usually with a median brown stripe; fore femur dorsally yellowish, ventrally black; fore tibia and tarsus brownish to blackish, dorsum of tibia usually yellowish. Mesonotum with three yellow longitudinal stripes; dorsal 3/4 of spiracle covered by golden setae; mesopleuron with a longitudinal stripe of golden setae, stripe sigmoid, curved, posteriorly discontinue; mesosternum without marks; middle and hind coxae and trochanters yellowish, trochanters ventrally with central brownish mark; middle and hind femora with sides yellowish, other areas blackish; middle and hind tibiae and tarsi yellowish to brown, middle tibia sometimes with blackish stripes. Metanotum black, anteriorly with a central pair of yellowish marks, posterolateral margin covered by golden setae; metacetabulum with a lateral stripe of golden setae; metapleuron and metasternum without marks. *Abdomen*: abdominal medio- and laterotergites I–VII black, lateral margins of mediotergites with a longitudinal stripe of golden setae; side of abdomen yellowish, with blackish marks and a stripe of golden setae. *Male*: posterior margin of abdominal mediotergite VII rounded. Posterior projection of last abdominal laterotergite (= connexival spine) triangular. Abdominal sterna II–VII without median depression. Sternum VII with a rounded notch on posterior margin, notch subequal in length and width. Abdominal segment VIII 1.9–2.1 times longer than wide, tubular; lateral margins diverging posteriorly, anterior width 9/10 of posterior width; right side with a pair of projections inserted ventrally, dorsalmost projection small and about as long as wide, ventralmost projection oval and about twice as long as wide, projections directed posteroventrally, inserted on the segment together, with apex rounded (Fig 19C–19F); venter anteriorly with a median depression that extends through about 2/3 of segment, posterior margin forming a large notch. Pygophore and proctiger sinistrally rotated about 90° (Fig 19A and 19C). Proctiger 1.1 times longer than abdominal mediotergite VII; right basolateral process developed, rounded, about 1/5 of left basolateral process; left basolateral process long, 1.7 times longer than wide, with 3/5 of proctiger length, longitudinal axis diverging about 90° of longitudinal axis of proctiger, lateral margins converging distally, apex acute, not bifid (Fig 19A). Abdominal segment VIII black dorsally, yellowish laterally and ventrally; pygophore yellowish; proctiger brown. *Female*: abdominal tergum VIII triangular, 1.1 times longer than wide, lateral margins strongly diverging posteriorly, posterior margin rounded (Fig 35C). Abdominal laterotergites not folded over mediotergites, except for last segment; posterior projection of last laterotergite (= connexival spine) long, tubular, about three times as long as mediotergite VII, apex rounded (Fig 35C). Expansion abdominal of sternum VII oval, completely covering first pair of gonocoxae, not folded over tergum VIII, about as wide as long, with an oval lobule, lobule with 1/3 of sternum VII length (Fig 19B); anal cone short. Abdominal tergum VIII black with yellowish lateral margins; extension of sternum VII yellowish.

#### Comments

The male of *P*. *woytkowskii* has the pygophore and proctiger sinistrally rotated about 90° (Fig 19A and 19C), similar to *P*. *anchicaya*, *P*. *carvalhoi*, *P*. *molanoi* Floriano and Moreira, **sp. nov.**, *P*. *peruvianus*, *P*. *shuar*, *P*. *spiculus*, *P*. *sumaco*, *P*. *tridentatus*, *P*. *variabilis*, *P*. *vivatus*, and *P*. *williamsi*. It differs from all these species by the right posterolateral angle of male abdominal segment VIII with a pair of projections inserted ventrally, with the dorsalmost projection small and as long as wide, and the ventralmost projection larger, oval, and twice as long as wide (Fig 19C–19F). Furthermore, the female of *P*. *woytkowskii* has a long, about as wide as long, lobule on abdominal sternum VIII (Fig 19B).

**Geographic distribution (Fig 40).** PERU: **San Martín** [14, 17, this work].

#### Type material examined

1♀, 1♂ paratypes (USNM): ‘Peru S.A.\ Sept9–Oct.3’36\ F.Woytkowski\ No.3682’ ‘Vic Rioja\ Depto. San Martin\ Jungle 900 m.a.s.l’ ‘Paratype\ *Potamobates*\ *woytkowskii*\ H. B. Hungerford’ ‘J. T. Polhemus\ Collection 2011\ C.J.Drake Accession’.

#### Additional material examined

1♂ (USNM): ‘Peru S. A\ Oct. 11–22, 1936\ F. Woytkowski\ No.3722’ ‘Dept. San Martin\ Vic. Rioja\ Jungle 900 m.a.s.l’ ‘♂’ ‘Woytkowskii ♂’ ‘J. T. Polhemus\ Collection 2011\ C.J.Drake Accession’.

### *Brailovskybates* Floriano and Moreira, gen. nov.

(Figs 4 and 6)

urn:lsid:zoobank.org:act:C2FA5850-0932-4123-B1D8-81800D04E8E3

*Potamobates*; Hungerford 1937a [13]: 63–65 (new species). Drake and Harris (1938): 74, 75 (record). Kuitert (1942) [17]: 140, 142 (key, records). Matsuda (1960) [2]: 64, 100, 111, 120, 125, 131, 142, 219, 221–224, 228–230, 510–515 (figures, morphology). Polhemus and Polhemus (1995) [6]: 364, 366–368 (key, phylogeny). Cognato (1998) [18]: 21, 22 (phylogeny). Padilla-Gil and Damgaard (2011) [7]: 44, 47, 48 (key, phylogeny); *non* Champion (1898) (in part; *P*. *thomasi*).

#### Diagnosis

Mandibular and maxillary plates fused; epistomal suture absent; labium short, reaching at most anterior margin of mesosternum; middle and hind pretarsal claws absent; abdomen elongated, longer than mesothorax; abdominal spiracles located at the center of segments. *Male*: posterior projection of last abdominal laterotergite (= connexival spine) short, apex acute; abdominal segment VIII without projections; pygophore and proctiger not rotated in relation to longitudinal axis of body (Fig 17A). *Female*: posterior margin of abdominal sternum VII not produced medially, with a pair of lateral projections (Fig 17C); abdominal tergum VIII subequal in length and width (Fig 17B).

#### Description

Length (♂: 11.8–13.6 mm; ♀: 12.2–14.0); width (♂: 2.8–3.7; ♀: 3.4–3.9). *Head*: width through eyes 1.2–1.3 times larger than head length. Eye reniform, greatest width 1.2 times larger than minimum interocular distance. Mandibular and maxillary plates fused. Clypeus rounded anteriorly; epistomal suture absent. Antenniferous tubercle protuberant. Antennomere I 2.7 times longer than II, 0.7 times of length of II + III; II 1.3 times longer than III; IV subequal to 1.1 times longer than III. Labium short, reaching at most anterior portion of mesosternum (Fig 4D); articles I and II short; III 1.5–1.8 times longer than IV. *Thorax*: pronotum with a yellowish median stripe (Fig 4A and 4C); propleuron with a longitudinal stripe of golden setae; proacetabulum lacking stripe of golden setae. Fore femur about 1.5 times longer than fore tibia, proximal region without denticles; fore tarsomere II 2.4–2.7 times longer than I. Mesonotum with median length subequal to width through mesoacetabula, with a median and two lateral yellowish longitudinal stripes; mesopleuron with a continuous, straight, longitudinal stripe of golden setae. Middle femur subequal in length to hind femur, 1.6 times longer than middle tibia; middle tarsomere I about 3.4 times longer than II; middle pretarsal claws absent. Metanotum with a median yellowish stripe, width through acetabula 2.0–2.5 times median length; metapleuron with a longitudinal stripe of golden setae. Hind femur about 1.6 times longer than hind tibia; hind tarsomere I about 2.0 times longer than II; hind pretarsal claws absent. Metathoracic scent apparatus well developed. *Abdomen*: elongated, longer than mesothorax, dorsum with yellowish median stripe; lateral margins of mediotergites with inconspicuous stripe of golden setae; spiracles located at the center of segments. Mediotergite I 1.0–1.2 longer than II; II with 7/10 to 9/10 of length of III; III–V progressively longer. *Male*: posterior projection of last abdominal laterotergite (= connexival spine) short, apex rounded to triangular (Fig 6A). Posterior margin of abdominal sternum VII with a rectangular notch, notch almost three times as wide as long (Fig 4B). Abdominal segment VIII dorsally almost as long as wide, without projections (Fig 6A). Pygophore and proctiger not rotated in relation to longitudinal axis of body. Proctiger asymmetrical; right basolateral process developed, oval, slightly shorter than left process (Fig 6G); left basolateral process short (Fig 6G). Parameres reduced. Phallus with dorsal sclerite short, very narrow (Fig 6D); base of ventral sclerite slightly notched (Fig 6F). *Female*: posterior projection of last abdominal laterotergite (= connexival spine) present, triangular, short (Fig 6B); posterior margin of abdominal sternum VII not produced medially, with a pair of lateral projections (Fig 6C); abdominal tergum VIII subequal in length and width (Fig 6B).

#### Etymology

The new genus is dedicated to Dr. Harry Urad Brailovsky Alperowitz (Universidad Nacional Autónoma de México), in recognition of his contributions to the knowledge of Mexican Heteroptera.

#### Comments

*Potamobates thomasi* does not fit morphologically among its current congeners or within the other genera of Cylindrostethinae. Furthermore, in a previous phylogenetic analysis based on morphological data [20], it was recovered as sister to (*Potamobates* + *Platygerris*), with one autapomorphy: apex of ventral sclerite of male phallus strongly widened. Therefore, we erect the genus *Brailovskybates* Floriano and Moreira, **gen. nov.** to hold *Po*. *thomasi*.

*Brailovskybates* Floriano and Moreira, **gen. nov.** can be distinguished from *Potamobates* based on the following differences: 1) mesonotum with three longitudinal yellow stripes, abdominal mediotergites with a median yellow stripe (Fig 4A and 4C) vs. mesonotum entirely black or black with yellow marks (not longitudinal stripes), abdominal mediotergites without median yellow stripe (Figs 13A–16A); 2) abdomen elongated, length 5.5–6.6 times width at base vs. abdomen shorter, length 2.0–5.0 times width at base; 3) male abdominal segment VIII subequal in length and width (Fig 6A) vs. longer than wide (Figs 1A, 2A, 3A and 18A); 4) abdominal spiracles located at the center of segments vs. closer to the posterior margins than to the anterior margins of the segments; 5) male pygophore and proctiger not rotated in relation to the longitudinal axis of the body (Fig 6A) vs. rotated 20°–90° in relation to the longitudinal axis of the body (Figs 1A, 2A, 3A and 18A); 6) male abdominal segment VIII without projections (Fig 6A) vs. with projections (Figs 1G, 2A, 3F, 7B, 7E and 9B); 7) posterior margin of female abdominal sternum VII not produced medially, with a pair of lateral projections (Fig 6C) vs. produced medially, without lateral projections (Figs 1D, 2D, 7D, 8D, 10C and 12C); and 8) first pair of gonocoxae completely exposed (Fig 6C) vs. partially or completely covered by sternum VII (Figs 1D, 3D, 7D, 8D, 10C and 12C).

*Brailovskybates* Floriano and Moreira, **gen. nov.** is also different from *Cylindrostethus*, based mainly on the following features: 1) posterior projection of male last abdominal laterotergite (= connexival spine) short, with apex rounded (Fig 6A) vs. short or long, with acute apex; 2) phallus elongated, with dorsal and ventral sclerites, base of ventral sclerite slightly notched, transversal sclerite present (Fig 6D) vs. phallus oval or rounded (except in *C*. *quadrivittatus* Bergroth, 1916), either dorsal or ventral sclerite lost, base of ventral sclerite (when present) not notched, transversal sclerite absent; and 3) posterior margin of female abdominal sternum VII with a pair of lateral projections (Fig 6C) vs. without projections.

Finally, the following characteristics separate the new genus from *Platygerris*: 1) body long, 3.8 times larger than width through mesoacetabula vs body shorter, 1.5 times larger than width through mesoacetabula (except for male *Pl*. *caeruleus* Champion, 1898, where abdominal segment VIII is extremely long); 2) mesonotum without a transversal C-shaped stripe formed by silvery setae vs. mesonotum with such stripe; 3) metathoracic scent apparatus well developed (Fig 5F) vs. not developed; 4) abdomen as long as thorax (Fig 4A) vs clearly shorter than thorax (also except for male *Pl*. *caeruleus*); 5) male pygophore and proctiger not rotated in relation to the longitudinal axis of the body (Fig 6A) vs. rotated; and 6) female abdominal sternum VII without a lateral plate or expansion, but with a pair of lateral projections (Fig 6C) vs. with a lateral plate or expansion, but without pair of lateral projections.

#### Geographic distribution

Endemic to southwestern Mexico.

#### Type species

*Potamobates thomasi* Hungerford, 1937, by present designation and monotypy.

### *Brailovskybates thomasi* (Hungerford, 1937) (comb. nov.)

*Potamobates thomasi* Hungerford 1937a [13]: 63–65 (description). Drake & Harris (1938) [15]: 74, 75 (record). Kuitert (1942) [17]: 140, 142 (key, records). Matsuda (1960) [2]: 64, 100, 111, 120, 125, 131, 142, 219, 221–224, 228–230, 510–515 (figures, morphology). Polhemus and Polhemus (1995) [6]: 364, 366–368 (key, phylogeny). Cognato (1998) [18]: 21, 22 (phylogeny). Padilla-Gil and Damgaard (2011) [7]: 44, 47, 48 (key, phylogeny).

#### Diagnosis

As for the genus.

#### Redescription

Length (**♂**: 11.8–13.6 mm; ♀: 12.2–14.0); width (**♂**: 2.8–3.7; ♀: 3.4–3.9) (Fig 4). *Head*: antennomere I about 1.3 times longer than head width, about 2.7 times longer than antennomere II; II about 1.3 times longer than III; IV subequal to 1.1 times longer than III. Eye width 1.2 times interocular width. Article III of labium 1.5–1.8 times longer than IV. Antenna black; dorsum of head black, vertex with a yellowish mark; base of antenniferous tubercle with a yellowish stripe, apex blackish; mandibular and maxillary plates yellowish; labium with articles I and II yellowish, III and IV black; venter of head yellowish. *Thorax*: pronotum with a yellowish median stripe, posterior margin not covered by golden setae; propleuron with a longitudinal stripe of golden setae; proacetabulum yellow with apical margin blackish, without patch of golden setae; fore coxa yellowish; fore trochanter with mesal region, basolateral 1/3 and ventrodistal 2/3 yellowish, rest of segment blackish; proximal region of fore remur without denticles; fore femur with longitudinal blackish stripe dorsally and ventrally; fore tibia and tarsus blackish. Mesonotum with a median and two lateral yellowish longitudinal stripes, posterior margin with golden setae; spiracle with a yellowish mark, posterior margin covered by golden setae; mesopleuron with a continuous, straight, longitudinal stripe of golden setae; mesosternum with an anterior blackish mark near proacetabula; meso- and metacetabula black, with a stripe of golden setae laterally; middle and hind coxae yellowish, with a lateral black mark each; dorsum of middle and hind trochanters black, venter brown; middle and hind femora light-brown basally on venter, remaining of femora, and middle and hind tibiae and tarsi black. Metanotum with a yellowish median stripe, posterior margin covered by golden setae; metapleuron with a longitudinal stripe of golden setae; metasternum yellowish. *Abdomen*: mediotergites I–VII black, lateral margins with an inconspicuous stripe of golden setae, median region with a yellowish stripe; posterior margin of mediotergite VII sometimes yellowish; laterotergites with dorsalmost 1/3 yellowish; side of abdomen black; limit between sterna with a transverse, narrow, lateral stripe nearly reaching the central region; sterna VII–VII light-yellow. *Male*: abdominal sterna V and VI slightly depressed medially, remaining sterna without depressions. Posterior margin of abdominal mediotergite VII truncate. Posterior projection of last abdominal laterotergite (= connexival spine) short, apex rounded to triangular. Abdominal sternum VII with a rectangular notch, notch almost three times as wide as long. Abdominal segment VIII almost as long as wide, tubular; lateral margins slightly divergent and with a slight bulging on anterior 1/3; posterior margin dorsally rounded, unmodified. Pygophore and proctiger not rotated in relation to the longitudinal axis of body (Fig 6A). Pygophore 1.8 times longer than abdominal mediotergite VII; posteroventral region with denticles. Proctiger 1.3 times longer than abdominal mediotergite VII; on anterior margin, a notch with about 1/5 of proctiger length; right margin not folded ventrally; left margin folded only on basolateral process; apex with many black denticles; right basolateral process developed, oval, slightly shorter than left process; left basolateral process short, forming only a slight bulging, longitudinal axis diverging 90° from the longitudinal axis of proctiger, lateral margins converging distally, apex round to subacute (not bifid) (Fig 6G). Phallus relatively short, length about 4.3 times smallest width (Fig 6D); dorsal sclerite very narrow, short, subequal in length to ventral sclerite, apex notched centrally (Fig 6D); lateral sclerite triangular; ventral sclerite very wide (apex almost three times as wide as center), lateral margins dorsally curved, base with slight central notch (Fig 6F); transverse sclerite involving ventral sclerite centrally. Abdominal tergum VIII black, with a longitudinal basal stripe and lateral margins brown; side and venter of segment VIII yellowish, posterolateral margin covered by black setae; pygophore with a central yellowish mark at base, posterior region brownish to blackish; proctiger black. *Female*: abdominal laterotergites folded over mediotergites on segments IV–VII. Projection of last abdominal laterotergite (= connexival spine) present, triangular, short, with 1/4 of mediotergite VII length; apex acute. Posterior margin of abdominal sternum VII not produced medially, with a pair of lateral projections. First pair of gonocoxae completely exposed (Fig 6C). Abdominal tergum VIII with length 0.8–1.3 times its width; lateral margins slightly divergent posteriorly; posterior margin truncate (Fig 6B). Anal cone short, half as long as abdominal mediotergite VII. Abdominal tergum VIII black, with a triangular longitudinal mark slightly lighter; lateral margins yellowish. First pair of gonocoxae yellowish, with black margins. Anal cone black. *Macropterous*: posterolateral margin of pronotum yellowish.

#### Variation

The fore femur can be black with about the basal half posteriorly yellowish and a longitudinal yellow stripe reaching the dorsum at the apex.

**Fig 42:**
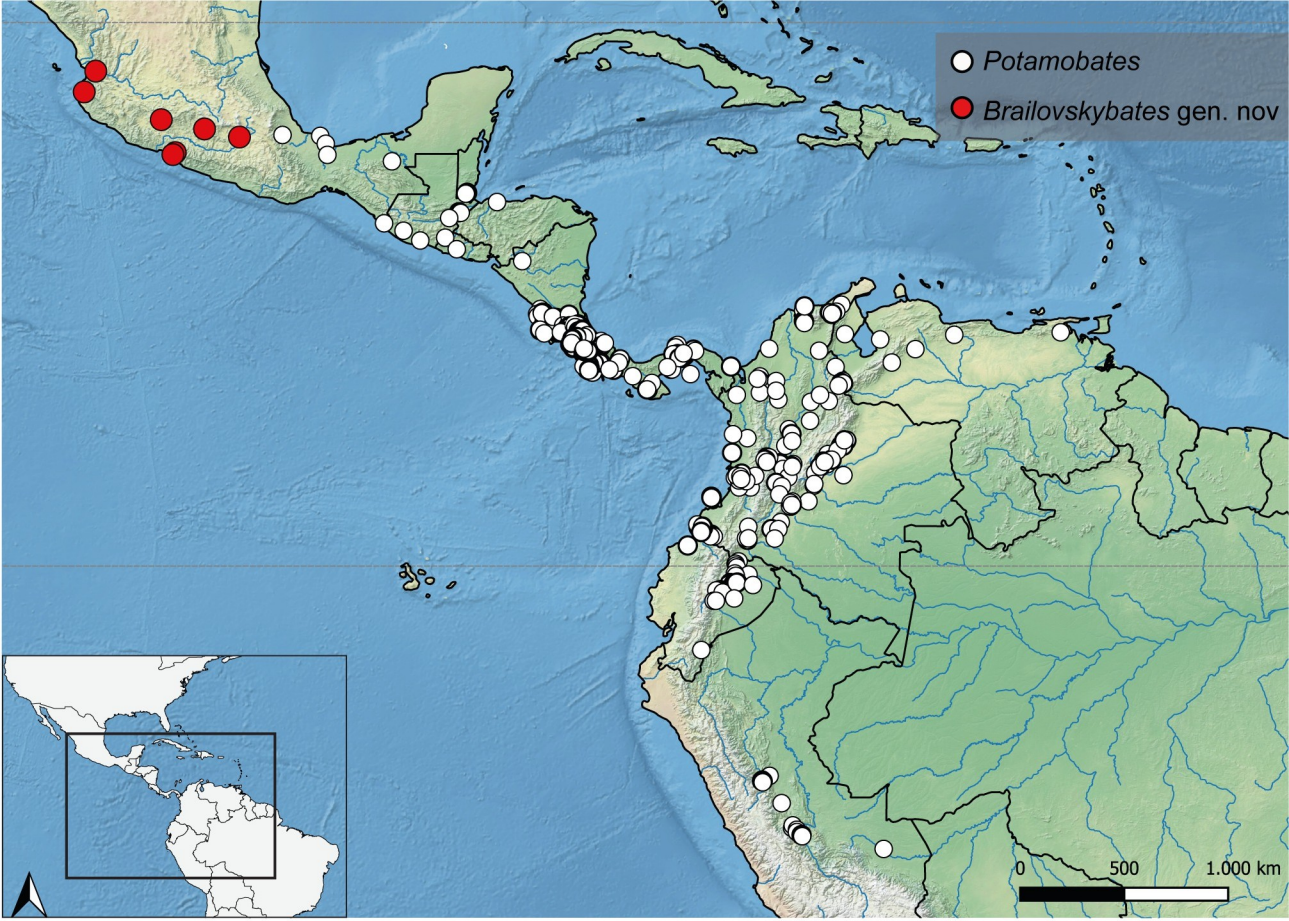
Map showing the geographical distribution of species of *Brailovskybates* new. genus and *Potamobates*. Spatial data from Natural Earth (http://www.naturalearthdata.com/).

**Geographic distribution** (Fig 42). MEXICO: **Guerrero** [this work], **Jalisco** [this work], **Mexico** [17, this work], **Michoacán** [13, 17, this work], **Nayarit** [this work], **Puebla** [this work].

#### Type material examined

1♀ paratype (USNM): ‘El Sabino Uruap\ Mich. Mex. 7 25 36\ H. D. Thomas’ ‘PARATYPE\ *Potamobates*\ *thomasi*\ H. B. Hungerford’ ‘J. T. Polhemus\ Collection 2014\ C. J.Drake Accession’.

#### Additional material examined

1♀, 1♂ (UCMC): ‘MEXICO, Guerrero\ Aguacotillo, km 40 on\ Mex. 134, NE Ixtapa\ CL 1893 I–27–85\ J. T. Polhemus’ ‘*Potamobates*\ *thomasi*\ Hungerford’. 1♀ (UCMC): ‘MEXICO, Guerrero\ Aguacotillo, km 40 on\ Mex. 134, NE of Ixtapa\ CL 1893 I–27–85\ J. T. Polhemus’. 1♀ (USNM): ‘Tejupilco, Mex.\ Temascaltepec\ VI–16–33’ ‘H. E. Hinton,\ R. L. Usinger\ Collectors’ ‘C J. Drake\ Coll. 1956’ ‘*Potamobates*\ *thomasi*\ Drake, Hung.’. 4♀, 11♂ (USNM): ‘‘MEXICO, Guerrero\ Aguacotillo, km 40 on\ Mex. 134, NE Ixtapa\ CL 1893 I–27–85\ J. T. Polhemus’ ‘J. T. Polhemus\ Collection 2014\ C.J. Drake Accession’. 1♀, 3♂ (USNM): ‘MEX., Nayarit\ San Blas (Riv.)\ CL1233 XI–28–68\ J.T. Polhemus’ ‘J. T. Polhemus\ Collection 2014\ C.J. Drake Accession’. 4♀, 2♂ (USNM): ‘MEX., Jal., 1000’ S. of Mismaloya\ CL733, VI–9–1975\ J. T. Polhemus’ ‘J. T. Polhemus\ Collection 2014\ C.J. Drake Accession’. 6♀, 2♂ (USNM): ‘MEX., Nayarit\ 5 de Mayo\ CL1027 21April1964\ J.T.& M.S.Polhemus’ ‘J. T. Polhemus\ Collection 2014\ C.J. Drake Accession’. 2♀, 4♂ (USNM): ‘MEX., Puebl: 8Mi w of\ Izucar de Matamoros\ CL1051 27Apr1964\ J. T.&M.S.Polhemus’ ‘J. T. Polhemus\ Collection 2014\ C.J. Drake Accession’. 1♀ (USNM): ‘MEX., Nay., 2800\ El Refugio, km. 29\ CL729, VI–8–1975\ J. T. Polhemus’ ‘J. T. Polhemus\ Collection 2014\ C.J. Drake Accession’. 2♀, 1♂ (USNM): ‘MEXICO, Guerrero\ Terrenos, km. 21 on Mex.\ 134, NE of Ixtapa Cl 1896\ I–29–85 J.T.Polhemus’ ‘J. T. Polhemus\ Collection 2014\ C.J. Drake Accession’.
